# Nonlinear and Emissive {[M^III^(CN)_6_]^3–^···Polyresorcinol} (M = Fe, Co, Cr) Cocrystals Exhibiting an Ultralow Frequency Raman Response

**DOI:** 10.1021/acs.inorgchem.3c03153

**Published:** 2023-12-18

**Authors:** Katarzyna Jędrzejowska, Jedrzej Kobylarczyk, Dominika Tabor, Monika Srebro-Hooper, Kunal Kumar, Guanping Li, Olaf Stefanczyk, Tadeusz M. Muzioł, Katarzyna Dziedzic-Kocurek, Shin-ichi Ohkoshi, Robert Podgajny

**Affiliations:** 1 Faculty of Chemistry, Jagiellonian University in Krakow, Gronostajowa 2, 30-387 Kraków, Poland; 2 Doctoral School of Exact and Natural Sciences, Jagiellonian University in Kraków, Prof. St. Łojasiewicza 11, 30-348 Kraków, Poland; 3 Institute of Nuclear Physics PAN, Radzikowskiego 152, 31-342 Kraków, Poland; 4 Department of Chemistry, School of Science, The University of Tokyo, 7-3-1 Hongo, Bunkyo-ku, Tokyo 113-0033, Japan; 5 Faculty of Chemistry, Nicolaus Copernicus University in Toruń, Gagarina 7, 87-100 Toruń, Poland; 6 Marian Smoluchowski Institute of Physics, Jagiellonian University, Łojasiewicza 11, 30-348 Krakow, Poland

## Abstract

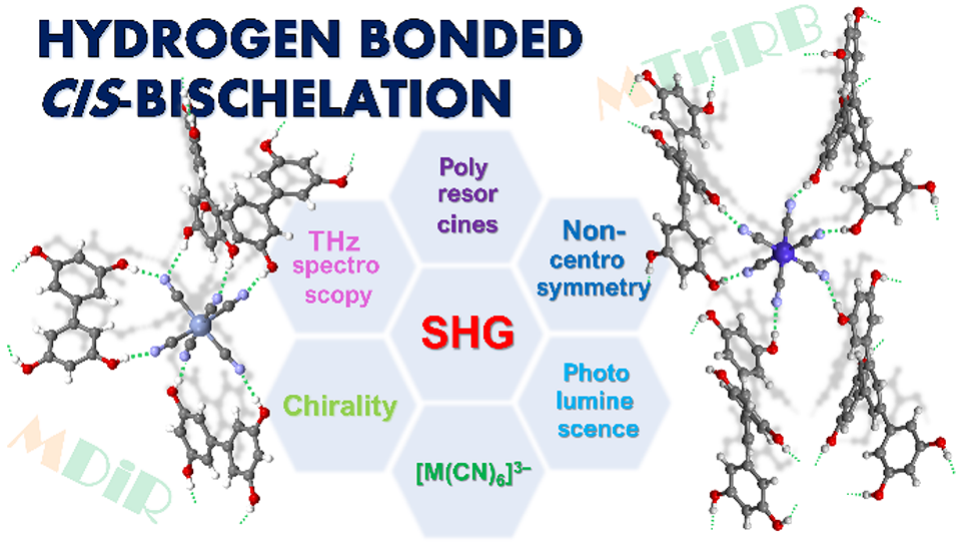

Optically active functional noncentrosymmetric architectures might be achieved through the combination of molecules with inscribed optical responses and species of dedicated tectonic character. Herein, we present the new series of noncentrosymmetric cocrystal salt solvates (PPh_4_)_3_[M(CN)_6_](L)_*n*_·*m*solv (M = Cr(III), Fe(III), Co(III); L = polyresorcinol coformers, multiple hydrogen bond donors: 3,3′,5,5′-tetrahydroxy-1,19-biphenyl, DiR, *n* = 2, or 5′-(3,5-dihydroxyphenyl)-3,3″,5,5″-tetrahydroxy-1,19:3′,1″-terphenyl, TriRB, *n* = 1) denoted as **MDiR** and **MTriRB**, respectively. The hydrogen-bonded subnetworks {[M(CN)_6_]^3–^;L_*n*_}_∞_ of *dmp*, *neb*, or *dia* topology are formed through structural matching between building blocks within supramolecular *cis*-bis(chelate)-like {[M(CN)_6_]^3–^;(**H**_**2**_L)_2_(**H**L)_2_} or tris(chelate)-like {[M(CN)_6_]^3–^;(**H**_**2**_L)_3_} fragments. The quantum-chemical analysis demonstrates the mixed electrostatic and covalent character of these interactions, with their strength clearly enhanced due to the negative charge of the hydrogen bond acceptor metal complex. The corresponding interaction energy is also dependent on the geometry of the contact and size matching of its components, rotational degree of freedom and extent of the π-electron system of the coformer, and overall fit to the molecular surroundings. Symmetry of the crystal lattices is correlated with the local symmetry of coformers and {complex;(coformer)_*n*_} hydrogen-bonded motifs characterized by the absence of the inversion center and mirror plane. All compounds reveal second-harmonic generation activity and photoluminescence diversified by individual UV–vis spectral characteristics of the components, and interesting low-frequency Raman scattering spectra within the subterahertz spectroscopic domain. Vibrational (infrared/Raman), UV–vis electronic absorption (experimental and calculated), and ^57^Fe Mössbauer spectra together with electrospray ionization mass spectrometry (ESI-MS) data are provided for the complete description of our systems.

## Abstract 2

The series of non-centrosymmetric cocrystal salt solvates reveal hydrogen-bonded architectures {[M(CN)_6_]^3–^;L_*n*_}_∞_ involving supramolecular *cis*-bis(chelate) like {[M(CN)_6_]^3–^;(**H_2_**L)_2_(**H**L)_2_} or tris(chelate) like {[M(CN)_6_]^3–^;(**H_2_**L)_3_} motifs, L standing for linear bis-resorcinol DiR or triangular tris-resorcinol TriRB multiple hydrogen bond donors. All networks show second harmonic generation (SHG) and photoluminescence responses conditioned by the individual optical UV-Vis absorption cut-off thresholds.

## Introduction

Optical properties of matter based on nonlinear response and/or photoluminescence have attracted strong interest in fields such as bioimaging,^1−5^ anticancer therapy,^6^ or multifunctional molecular materials for photonic application, *e.g*. toward optical modulation of light^7^ and for luminescent thermometry.^8,9^ The phenomenon known as second-harmonic generation (SHG) has been extensively researched and utilized due to its ability to convert two incident photons into a single emitted photon with a doubled frequency. This process relies on the interaction between matter and the incident beam, specifically, its polarization and orientation. The advantage of SHG lies in its narrow operating band, which allows for the precise control and manipulation of the generated signal.^10,11^ The SHG function in the solid state requires a noncentrosymmetric space group and proper light absorption cutoff, which stimulated extended studies on the relevant inorganic,^12,13^ organic,^14−17^ as well as hybrid inorganic–organic^18−24^ phases. In particular, the two latter composition strategies have recently been employed within the cocrystallization approach^25,26^ considering the tunable intrinsic features of organic counterparts, *e.g*. hyperpolarizability, and tectonic character that allows for the desired supramolecular organization through noncovalent interactions.^14,16,17,27^ For example, hydrogen-bonded synthons are exploited in *directional* and *cooperative* extension toward supramolecular architectures,^27−31^ especially if one combines multisite complementary H-bond donor and acceptor or/and slightly modifies the already established contacts via judicious molecular replacement.^14,29^ The effective self-assembly process is usually supported by other ubiquitous weak interactions (π···π, ion···π, C–H···π, etc.). The potential of such a strategy was illustrated by the acquisition of materials that fulfill the technological demands for SHG performance competitive with the traditional ones (*see above*) as well as by efficient enantiospecific molecular recognition toward enantiopure crystal growth.^15,32−35^ Moreover, a hybrid approach involving 3d and 4f metal ion coordination complexes and their salts allows for the introduction of other functions, such as anion binding,^4,23^ specific luminescence for ratiometric or thermometric performance,^8,9^ single-ion magnetic properties for ultrahigh density data recording, storage, and processing,^8,9^ or switchable structural and dielectric loss properties owing to the phase transitions inscribed in the structure of molecular fragments and their positioning in the crystal, as commonly observed in molecular hybrid perovskites.^36−39^ In parallel, phonon properties of molecular materials have been studied by terahertz (THz) spectroscopy^9,40−42^ in the context of the optimization of their luminescent response (“management” of nonradiative vibrational loss)^43,44^ and slow magnetic relaxation (“management” of the effective energy barrier)^45^ or the generation of the THz signal through optical stimulation.^41^

**Scheme 1:**
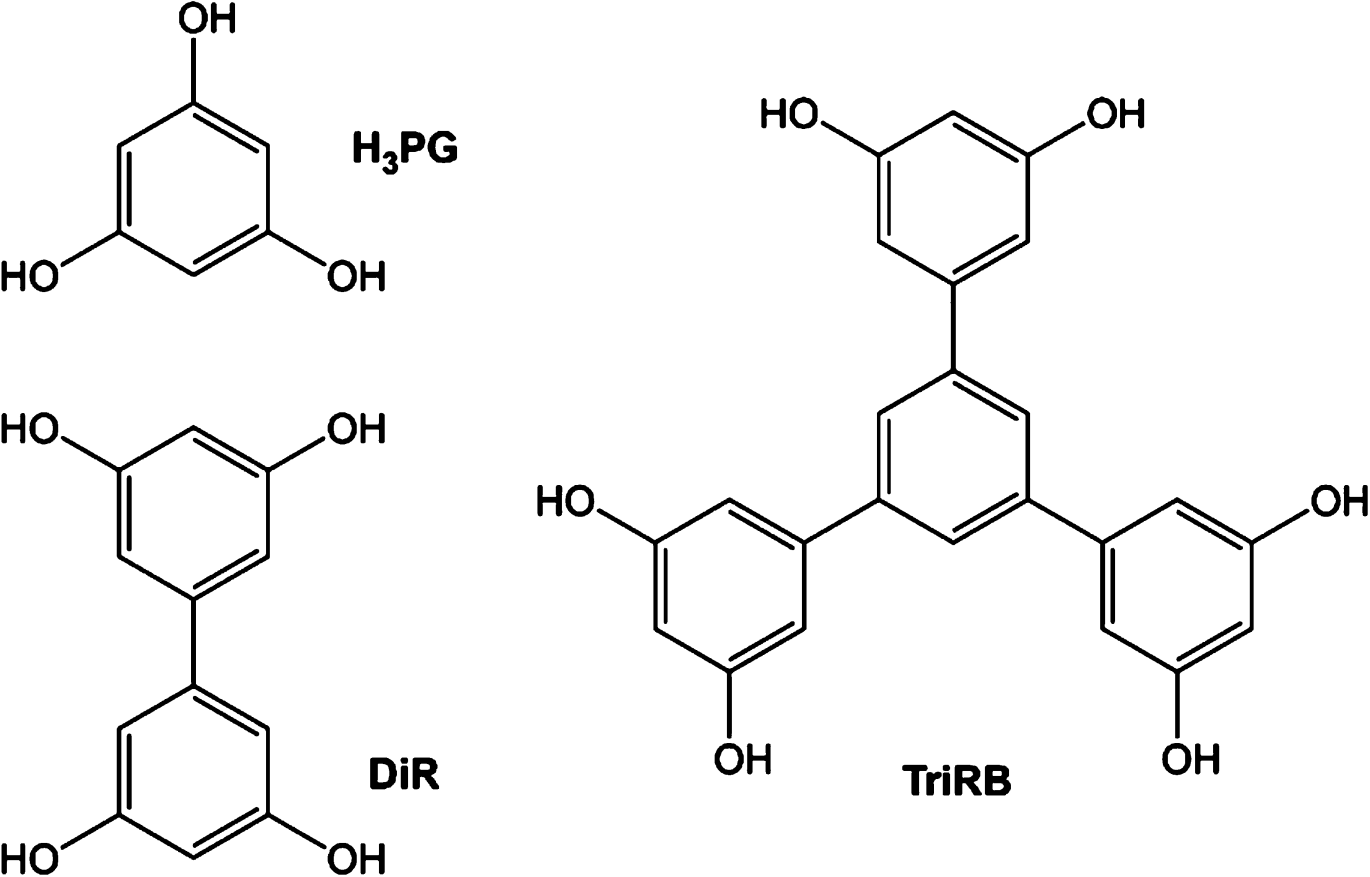
Molecular Structure of Hydrogen Bond Donor Coformers DiR and TriRB were not exploited previously in the syntheses of cocrystals.

**Figure 1:**
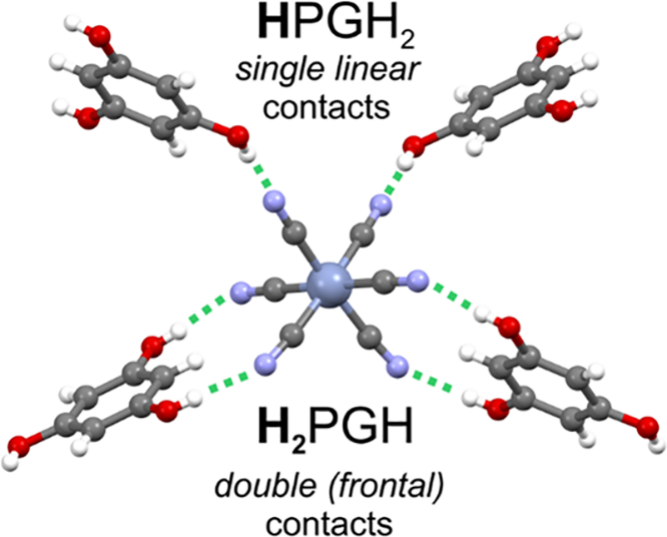
Supramolecular *cis*-bis(chelate) hydrogen-bonded motifs in **MH**_**3**_**PG**(52) comprising two double cyclic ring-type synthons {[M(CN)_6_]^3–^;**H**_**2**_PGH}, each formed between the *cis*-oriented cyanido ligands of [M(CN)_6_]^3–^ and resorcinol-like face of H_3_PG, and two single linear synthons {[M(CN)_6_]^3–^;**H**PGH_2_}. The above cyclic synthons represent the *frontal* mode of hydrogen bond donation; for the depiction of all cyclic H-bond synthons appearing in the whole family, see Figures 2 and 3.

In our research, we focus on the development of new noncovalent synthons for the construction of molecular architectures offering tunable optical properties. Over the past few years, we have been exploring the realm of cocrystal salt solvates that involve polycyanidometallate [M(CN)_*x*_]^*n–*^ and organic coformers with the objective to design and generate modular patterns of noncovalent interactions within these structures. In this context, a family of structurally related charge-transfer (CT) systems comprising 1,4,5,8,9,12-hexaazatriphenylene-hexacarbonitrile (HAT(CN)_6_) and tetracyanopyrazine (TCP) π-acids was investigated both in the solid state and solution. These assemblies exhibited anion-π interactions, the nature and strength of which were influenced by the size and shape of the contact components.^46−50^ Notably, our studies introduced the first instances of binary core–shell crystals based on anion-π interactions.^50^ Then, following the works of Desiraju and Paul on multicomponent topological CT-colored resorcinol (1,3-dihydroxybenzene) based systems^29^ and the pioneering works of Oshio and co-workers on phloroglucinol-conditioned (H_3_PG, 1,3,5-trihydroxybenzene) spin-crossover cyanido-bridged square Co_2_Fe_2_ complexes,^51^ we explored further possible schemes of noncovalent interactions between multiple hydrogen bond donor H_3_PG (Scheme 1) and mononuclear^52^ and polynuclear^53^ cyanido-complexes. As a result, we have discovered the unique supramolecular *cis*-bis(chelate) {[M(CN)_6_]^3–^;(H_3_PG)_4_} (M = Cr(III), Fe(III), Co(III)) motifs within the **MH**_**3**_**PG** architectures involving the following: (*i*) two double cyclic hydrogen bond synthons M(−CN···HO−)_2_Ar, {[M(CN)_6_]^3–^;**H**_**2**_PGH}, between *cis*-oriented cyanido ligands of [M(CN)_6_]^3–^ and the resorcinol-like face of H_3_PG, and (*ii*) two single hydrogen bonds M-CN···HO-Ar, {[M(CN)_6_]^3–^;**H**PGH_2_}, involving the remaining two cyanide ligands (Figure 1).^52^ Spectroscopic and computational descriptions revealed notable strength of the underlying interactions. While the local symmetry of the {[M(CN)_6_]^3–^;(H_3_PG)_4_} motif might be approached with the chiral *C*_2_ point group, interesting from the standpoint of the noncentrosymmetric and enantiopure resolution, regrettably, the centrosymmetric C2/*c* space group was observed for these crystals. Thus, we have expanded the boundaries for the synthesis of noncentrosymmetric architectures by introducing cocrystal salt solvates of (PPh_4_)_3_[M(CN)_6_](L)_*n*_·*m*solv (M = Cr(III), Fe(III), Co(III); L = coformers (Scheme 1): 3,3′,5,5′-tetrahydroxy-1,19-biphenyl, DiR,^54^*n* = 2, or 5′-(3,5-dihydroxyphenyl)-3,3″,5,5″-tetrahydroxy-1,19:3′,1″-terphenyl, TriRB,^54^*n* = 1) hereafter denoted as **CrDiR**, **FeDiR**, **CoDiR**, **CrTriRB**, **FeTriRB**, and **CoTriRB**, with the lattice symmetry dictated by the effective symmetry of coformers and the {complex;coformer} arrangement guided by structurally matched hydrogen bond synthons. The presented systems were thoroughly characterized via both experimental and computational (density functional theory, DFT) studies. These compounds are SHG active according to the nonzero second-order nonlinear optical (NLO) susceptibility tensor (χ_*ijk*_). Additionally, they exhibit diverse photoluminescence properties influenced by the distinct UV–vis spectral characteristics of the individual components. Finally, they display intriguing low-frequency (LF) Raman scattering spectra within the sub-THz spectroscopic range, which provides further insights into their structural and vibrational properties.

## Results and Discussion

### Structural Studies

All **MDiR** compounds, comprising the coformer DiR, crystallize in the orthorhombic system with a noncentrosymmetric space group of *Pna*2_1_. This results in the formation of a series of isomorphous analogs. On the other hand, the **MTriRB** networks, involving the coformer TriRB, display noticeable diversity as far as the space groups are concerned. Specifically, **CrTriRB** and **CoTriRB** crystallize in the monoclinic system, with **CrTriRB** having a noncentrosymmetric *Cc* space group and **CoTriRB** having a *P*2_1_ Sohncke space group. In contrast, **FeTriRB** crystallizes in the orthorhombic system, with a noncentrosymmetric *P*2_1_2_1_2 Sohncke space group. Nevertheless, the local structural arrangement is similar across the whole **MTriRB** series. For detailed crystal data and structure refinement parameters, see the Supporting Information (SI) and Tables S1 and S2. The uniformity of the powder samples and the identity of the crystals examined with single-crystal X-ray diffraction (SC XRD) experiments were confirmed by room-temperature (RT) powder X-ray diffraction (PXRD) measurements (Figure S1). The symmetrically independent parts are presented in Figures S2 and S3 in the SI. All crystal structures consist of PPh_4_^+^ cations, [M(CN)_6_]^3–^ anions, polyresorcinol DiR or TriRB coformer molecules (denoted also as L), and crystallization solvent MeCN and/or MeOH molecules.

**Figure 2:**
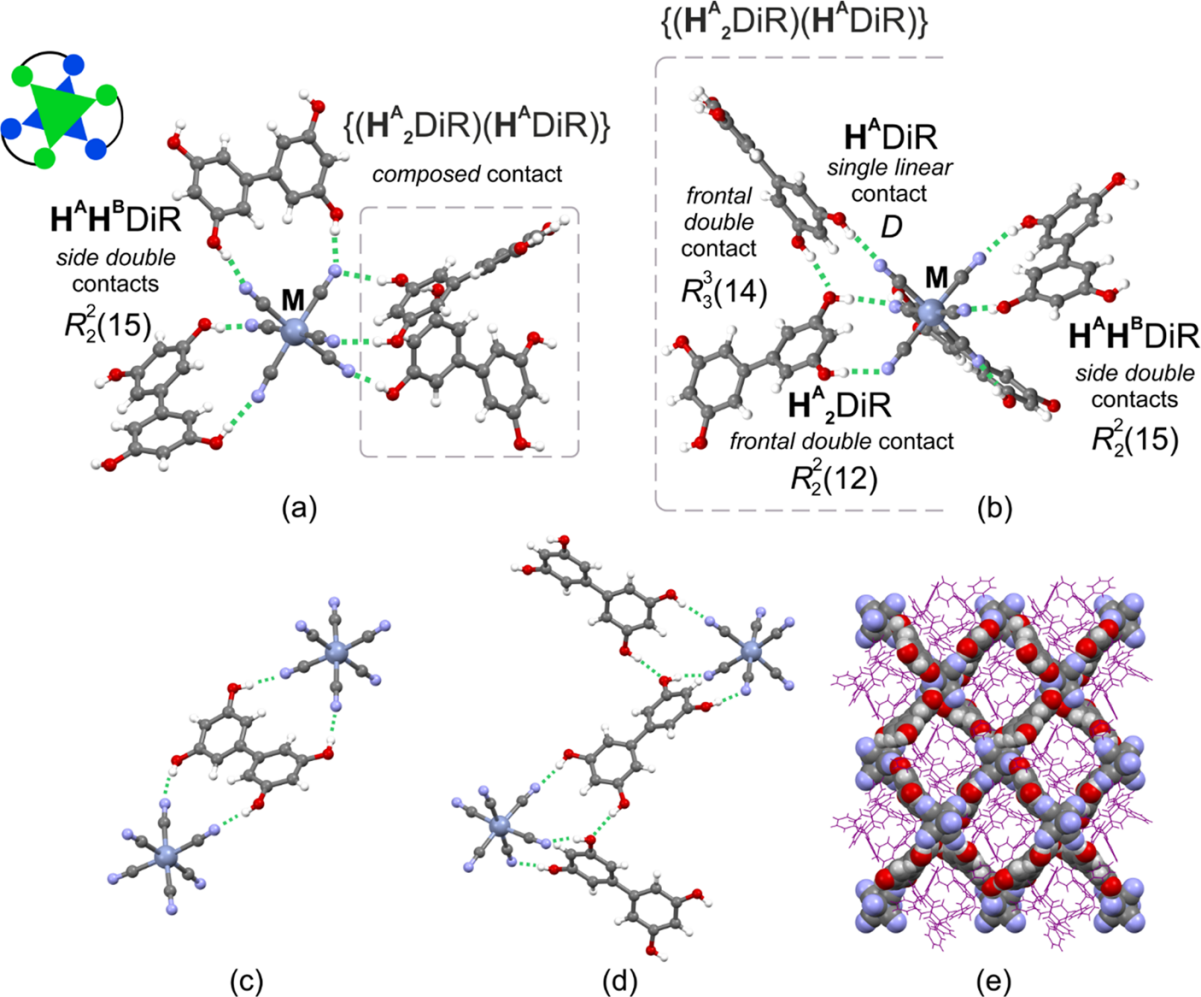
Crystal structure of **MDiR**: (a) and (b) two projections of the supramolecular hydrogen-bonded mixed *cis*-bis(chelate)/tris(chelate) {[M(CN)_6_]^3–^;(**H**^**A**^**H**^**B**^DiR)_2_(**H**^**A**^_**2**_DiR)(**H**^**A**^DiR)} fragment highlighting the *side double* cyclic  synthon and *composed* contact including one *single linear D* and two *frontal double* and  synthons (for metric parameters, see Table S5) together with the pictorial illustration of the canonical *cis*-chelated [ML_3_] coordination complex; (c) the *side* mode of coformer DiR with two neighboring [M(CN)_6_]^3–^ anions; (d) the *frontal* mode of coformer DiR with two neighboring [M(CN)_6_]^3–^ anions; (e) projection of the hydrogen-bonded {[M(CN)_6_]^3–^;(**H**^**A**^**H**^**B**^DiR)_2_ (**H**_**2**_DiR)(**H**DiR)} layer along the [010] crystallographic direction. Legend: gray-blue – Cr, Fe, or Co; gray – C; blue – N; red – O; white – H; PPh_4_^+^ cations in (e) – purple. MeCN and MeOH solvent molecules were omitted for clarity.

**Figure 3:**
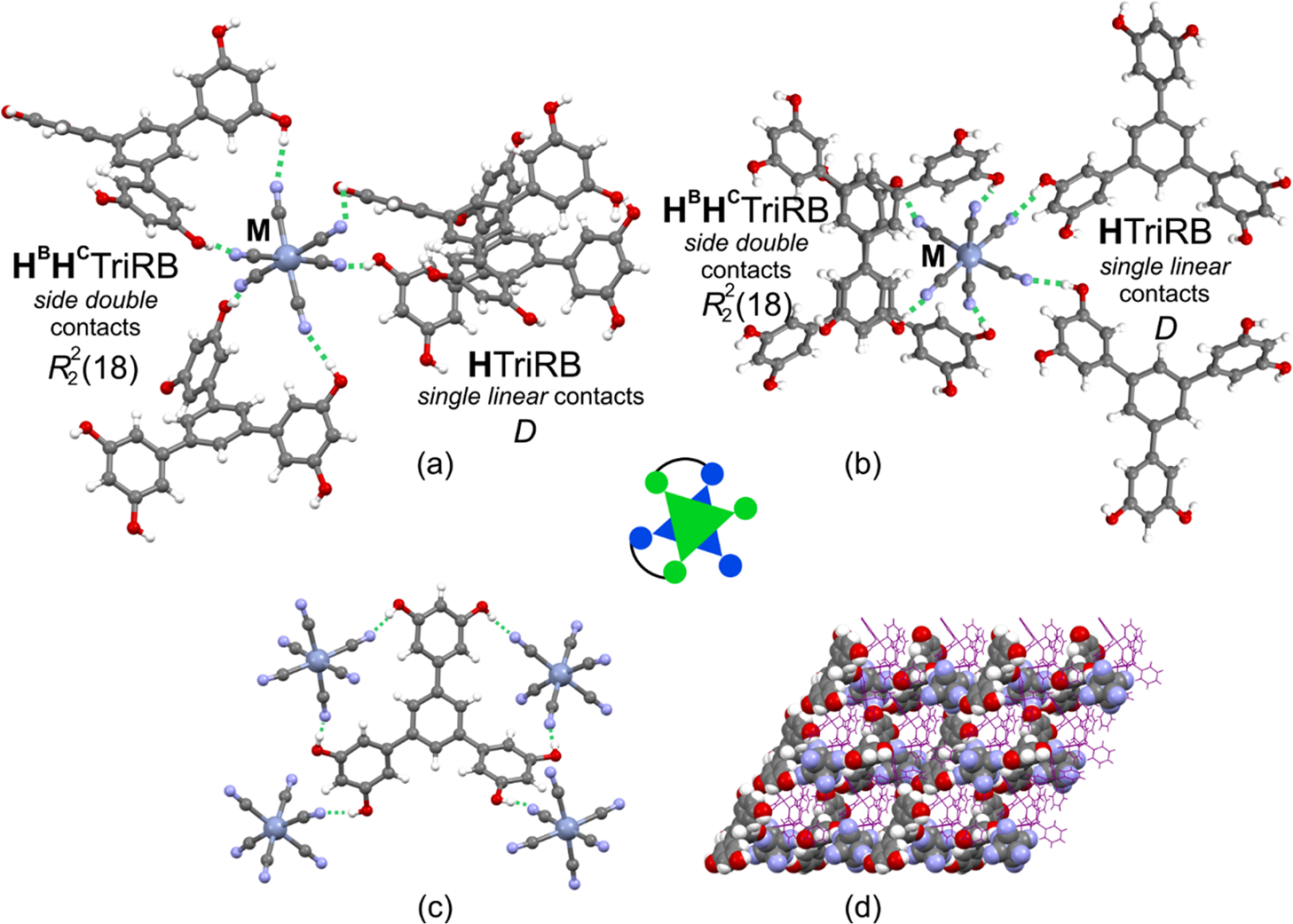
Crystal structure of **MTriRB**: (a) and (b) two projections of the supramolecular hydrogen-bonded *cis*-bis(chelate) {[M(CN)_6_]^3–^;(**H**^**B**^**H**^**C**^TriRB)_2_(**H**TiRB)_2_} fragment highlighting the *side double* cyclic  and *single linear D* synthons (for metric parameters, see Tables S6 and S7) together with the pictorial illustration of the canonical *cis*-chelated [ML_2_A_2_] coordination complex; (c) the coformer TriRB with four neighboring [M(CN)_6_]^3–^ anions; (d) projection of the hydrogen-bonded {[M(CN)_6_]^3–^;(**H**^**B**^**H**^**C**^TriRB)_2_(**H**TriRB)_2_} layer along the [010] crystallographic direction. Legend: gray-blue – Cr, Fe, or Co; gray – C; blue – N; red – O; white – H; PPh_4_^+^ cations in (d) – purple. MeCN and MeOH solvent molecules were omitted for clarity.

Detailed information on the most important bond lengths and angles is presented in Tables S3 and S4 in the SI. As shown in Figures 2 and 3, **MDiR** and **MTriRB** uniformly feature the 3D hydrogen-bonded {[M(CN)_6_]^3–^;L_*n*_}_∞_subnetworks exploiting the _L_O–H···N_M–C≡N_ contacts, which coexist with the multiple phenyl embraces (MPE) based subnetwork composed of PPh_4_^+^ cations assisted by solvent molecules (Figure S4). The observed N···O and N···H distances and O–H···N angles allow classification of them as medium-strength hydrogen bonding interactions (Table 1, Tables S5, S6, and S7).^55^ The lone pairs of O atoms additionally contribute to the stabilization of the network acting as the hydrogen bond acceptors, mainly from C–H groups (**MDiR** and **MTriRB**) and from some of the O–H groups (**MDiR**), Tables S8 and S9.

**Table 1:** Average Distances and Angles for Hydrogen Bond Contacts in the {[M(CN)_6_]^3–^;L_*n*_} Synthons of **MDiR** and **MTriRB**^a^^,^^b^

Contact	D···A/Å	H···A/Å	D–H···A/°	D···A/Å	H···A/Å	D–H···A/°	D···A/Å	H···A/Å	D–H···A/°
	**CrDiR**			**FeDiR**			**CoDiR**		
*Side*	2.72	1.89	169.1	2.75	1.93	166.1	2.75	1.93	170.3
*Frontal*	2.82	1.99	172.0	2.79	1.95	175.7	2.81	1.97	175.4
	**CrTriRB**			**FeTriRB**			**CoTriRB**		
*Side*	2.77	1.95	169.2	2.80	1.96	174.9	2.70	1.85	173.5
*Single linear*	2.69	1.87	166.2	2.71	1.87	174.4	2.72	1.89	169.0

a D – hydrogen bond donor L = DiR or TriRB, A – hydrogen bond acceptor [M(C)N_6_]^3–^.

b Compare with Figures 2 and 3 and Tables S5, S6, and S7 in the SI.

Within the {[M(CN)_6_]^3–^;DiR_2_}_∞_ subnetwork of **MDiR**, each [M(CN)_6_]^3–^ anion is surrounded by four DiR molecules through seven hydrogen bond contacts (Figure 2a,b). Among them, two DiR molecules are involved in the formation of double cyclic {[M(CN)_6_]^3–^;(**H**^**A**^**H**^**B**^DiR)} synthons of the  pattern^56^ to establish the {[M(CN)_6_]^3–^;(**H**^**A**^**H**^**B**^DiR)_2_} fragments (Figure 2a,b; see the motifs outside the dashed gray frames). Such contacts engage two O–H groups of different rings of one DiR molecule and two *cis*-oriented cyanido ligands, hereafter referred to as *side* synthons. Two other DiR molecules form a composed synthon {[M(CN)_6_]^3–^;(**H**^**A**^_**2**_DiR)(**H**^**A**^DiR)} through engaging the remaining pair of *cis*-oriented cyanido-ligands and one of the cyanido ligands involved in the {[M(CN)_6_]^3–^;(**H**^**A**^**H**^**B**^DiR)} interaction. Within this motif, both DiR molecules exploit two groups of the same ring to realize double *frontal* proton donation. Two cyclic synthons coexist in this fragment, featuring the *frontal* {[M(CN)_6_]^3–^;**H**^**A**^_**2**_DiR} pattern and *frontal* pattern, with the latter one involving an additional O–H···O hydrogen bond between two DiR molecules that accompanies the simple linear *D* pattern {[M(CN)_6_]^3–^;**H**^**A**^DiR} (Figure 2a,b; see the motifs within the dashed gray frames). Such an arrangement is the signature of strong competition between the resorcine-like fragments of different DiR molecules in the formation of the *frontal* cyclic  synthons with [M(CN)_6_]^3–^. Accordingly, two types of DiR linkers were found to interconnect M(CN)_6_]^3–^ anions, exploiting exclusively either the *side* mode or the *frontal* mode of hydrogen bond donation (Figure 2c,d). The resulting five-component {[M(CN)_6_]^3–^;(**H**^**A**^**H**^**B**^DiR)_2_(**H**^**A**^_**2**_DiR)(**H**^**A**^DiR)} fragment provides a unique low-symmetry supramolecular aggregate representing a hybrid of noncovalently bonded tris(chelate) and *cis*-bis(chelate) motifs, by analogy to the canonical six-coordinative [ML^1^_2_L^2^] and *cis*-[ML^1^_2_A_2_] complexes, respectively.

Within the {[M(CN)_6_]^3–^;TriRB}_∞_ subnetworks of **MTriRB**, each [M(CN)_6_]^3–^ anion is surrounded by four TriRB molecules through six hydrogen bond contacts (Figure 3a,b). Two TriRB molecules are involved in the formation of double cyclic ring-type {[M(CN)_6_]^3–^;(**H**^**B**^**H**^**C**^TriRB)} synthons of the  pattern to give the {[M(CN)_6_]^3–^;(**H**^**B**^**H**^**C**^TriRB)_2_} fragment (Figure 3a,b). These synthons exploit exclusively the *side* mode of hydrogen bonding interactions engaging two O–H groups of different rings of one TriRB molecule and two pairs of *cis*-oriented cyanido ligands. Two other TriRB molecules are connected with the remaining pair of *cis-*oriented cyanido groups via the single contacts of the linear *D* pattern, to produce two {[M(CN)_6_]^3–^;(**H**TriRB)} synthons. Accordingly, each TriRB linker interconnects four [M(CN)_6_]^3–^ anions, two of them through the double cyclic ring-type  synthons and two via the single linear *D* synthons, acting effectively as the 4-fold node (Figure 3c,d). The resulting five-component {[M(CN)_6_]^3–^;(**H**^**B**^**H**^**C**^TriRB)_2_(**H**TriRB)_2_} fragment realizes a unique low-symmetry supramolecular bis(chelate) aggregate, by analogy to the six-coordinative [ML^1^_2_A_2_] complexes.

A strong preference for the formation of the presented above hydrogen-bonded synthons in the gas phase was confirmed by the ESI-MS spectra exhibiting the representative progressive peak-sets attributable to the {(PPh_4_)_2_[Fe(CN)_6_]}^−^, {(PPh_4_)_2_[Fe(CN)_6_]L}^−^, and {(PPh_4_)_2_[Fe(CN)_6_]L_2_}^−^ aggregates in the negative ionization mode and to the {(PPh_4_)_4_[Fe(CN)_6_]}^+^ and {(PPh_4_)_4_[Fe(CN)_6_]L}^+^ aggregates in the positive ionization mode (Figures S5 and S6), which was properly reproduced by EnviPat software.^57^ The relative stability of the selected aggregates (in terms of DFT-computed interaction energy values) is discussed in the following.

The presented hydrogen-bonded subnetworks were achieved thanks to structural matching between the pairs of *cis*-oriented cyanido ligands in the metal complex acting as Brønsted bases and various pairs of O–H groups in polyresorcines, supported by an appropriate adaptive twist of their rings due to the natural degree of intramolecular rotation freedom. The occurrence of noncentrosymmetric or even enantiopure architectures might be related to the local symmetry of supramolecular bis(chelate) or tris(chelate) motifs and the local symmetry of coformers, all lacking both the inversion center and the mirror plane. Considering the intramolecular twist expressed by the interplanar angles between the rings A and B of DiR and between the central A ring and external B, C, and D rings of TriRB (Table S10 and Figures S7 and S8), the *D*_2_ point group might be assigned for DiR, whereas the *C*_2_ or *C*_1_ point group might be accessible in the case of TriRB (disregarding the positions of the phenolic protons and the resulting orientations of the O–H bonds with respect to the ring). It is important to note that the native crystals of DiR·2H_2_O and TriRB·2Me_2_CO·H_2_O grow in the centrosymmetric space groups *P*-1 and *P*2_1_/*c*, respectively; for the exact conformations within these crystals, see Table S10.^54^ One should also consider the role of the acentric PPh_4_^+^ cation. Screening of CSD database showed that out of 4523 crystal structures containing XPh_4_^+^ cations (with R_int_ not exceeding 10), 543 (12%) structures were noncentrosymmetric, the percentage being 2-fold or even 3-fold smaller compared to other structures involving the selected tetraalkylammonium or trialkylmethylammonium cations (see the SI). Such statistical results indicate that XPh_4_^+^ cations cannot provide a simple key to achieve a noncentrosymmetric solution. It is also plausible that a strong tendency of PPh_4_^+^ to form MPE interactions might impose additional limiting conditions on the local symmetry and space group that might be achieved. Interestingly, a considerable number of 251 structures containing XPh_4_^+^ (46.2% of the noncentrosymmetric structures found) belong to Sohncke space groups, whereas 193 structures crystallize in the space groups *P*2_1_ (87, 16.0% of 543), *Cc* (55, 10.2%) *Pna*2_1_ (39, 7.18%), and *P*2_1_2_1_2 (12, 2.21%) achieved in our studies (for details see the SI). This suggests that the results obtained within our series tend somehow to follow the trends of noncentrosymmetric space groups observed in the database within the regime of the search. To conclude, the occurrence of noncentrosymmetric space groups in our series is the result of the concentration of low-symmetry species lacking inversion centers or improper axes, with a possible decisive role of coformers and motifs they form with [M(CN)_6_]^3–^ complexes. We believe that exploration of the crystal structures and properties of similar cocrystal salts involving other organic cations (both having and lacking inversion centers) might shed more light on the above complex problem.

The hydrogen-bonded subnetworks were then described by topological analysis using TOPOS Pro software.^58^ The underlying building blocks were simplified to single points in space: [M(CN)_6_]^3–^ complexes were represented by metal ion sites, DiR molecules were represented by the centroids of the C–C bond between the rings, whereas TriRB molecules were represented by the centroids of their central ring (compare Figures 2 and 3). In line with the previous description, [M(CN)_6_]^3–^ and TriRB were treated as 4-connected nodes, and DiR was consistently considered the linker, disregarding the qualitative differences of particular intermolecular hydrogen-bonded connections. The hydrogen-bonded **MDiR** architecture revealed the *dmp* topology (Figure S9), whereas among the hydrogen-bonded **MTriRB** architectures two separate topologies are distinguished: **CrTriRB** (*Cc* space group) revealed rather the rare *neb* topology, while **FeTriRB** (*P*2_1_2_1_2 space group) and **CoTriRB** (*P*2_1_ space group) showed the frequently encountered *dia* topology (Figure S10). Consistently, **CrTriRB** includes 6^6^ topological motifs, which differ from the 6^4^ motifs noted for **FeTriRB** and **CoTriRB** (Figure S10c).^59^

### DFT Study on Hydrogen Bonding Interactions

The extended transition state-natural orbitals for chemical valence (ETS-NOCV)^60^ charge and bonding-energy decomposition analyses performed for the closed-shell motifs [Co(CN)_6_]^3–^/DiR and [Co(CN)_6_]^3–^/TriRB (see the SI for a description of the computational details and additional comments, and also Tables S12 and S14, Figures S14–S17 and S19–S20 for the calculated results) indicate that, similarly to what was previously found for **MH**_**3**_**PG**, hydrogen bonding {[M(CN)_6_]^3–^;DiR} and {[M(CN)_6_]^3–^;TriRB} interactions in **MDiR** and **MTriRB** are dominated by the electrostatic and orbital energy components, both enhanced by the negative charge of the hydrogen bond acceptor [M(CN)_6_]^3–^. In particular, the latter contribution stems not only from the σ-CT interaction between the occupied lone pair of nitrogen and the unoccupied σ* orbital of the O–H bond but also from the strong polarization (intra-CT) of the π-electron system within the hydrogen bond donor DiR or TriRB molecules, facilitated by the ion-dipole interaction.

Table 2 shows the DFT-calculated (B3LYP+D4//TZP) interaction energy values between the [M(CN)_6_]^3–^ and DiR, TriRB, and H_3_PG^52^ (as reference) building blocks in the molecular clusters extracted from the respective crystal structures (see also Tables S11 and S13). The interaction energies for *side double* synthons in **MDiR** ( and **MTriRB** () are between −50 and −54.5 kcal mol^–1^ and between −54.5 and −60 kcal mol^–1^, respectively. Notably smaller stabilization is observed for the *frontal double* interactions of the  pattern for **MDiR** and **MH**_**3**_**PG**, represented by the interaction energy values ranging from *ca*. −46 to −49 kcal mol^–1^ and from −44.5 to −48 kcal mol^–1^, respectively. A distinct trend in the absolute values of these energies can be thus established: **MTriRB** (*side double* – the strongest interaction) > **MDiR** (*side double*) > **MDiR** (*frontal double*) ≥ **MH**_**3**_**PG** (*frontal double* – the weakest interaction), which might be related to the size of π-electron system and degree of intramolecular rotation freedom of L. The *frontal double* synthons are rather rigid as the interacting −OH groups are attached to the same ring; this is reflected, for example, in the diversified metric parameters of the single _L_O–H···N_M–C≡N_ component in **MH**_**3**_**PG** congeners and the relevant energy of interactions.^52^ The flexibility of L increases in the order H_3_PG (rigid) < DiR < TriRB, which results in the increasing adaptability of the pair of phenolic groups attached to different rings to the steric demands of rigid *cis*-oriented pairs of cyanido ligands in [M(CN)_6_]^3–^. In the same order, an increase in the extent of the π-electron system across the molecule can be noted, which facilitates the possibility of the aforementioned intra-CT interactions within L leading to overall stronger orbital interactions and finally stronger total interactions between L and [M(CN)_6_]^3–^. Moreover, in the **MTriRB** and **MH**_**3**_**PG** molecular clusters, topologically identical *single linear D* contacts can be noted, characterized by interaction energies ranging from −27.5 to −31.5 kcal mol^–1^ and from −22 to −24.5 kcal mol^–1^, respectively, again reflecting the potentially larger adjustment freedom of TriRB in the overall scheme of intermolecular contacts and its larger size of the π-electron system. Finally, the interaction energy values for the whole molecular clusters {[M(CN)_6_]^3–^;L_4_} (all in kcal mol^–1^) vary from −163 to −171.5 for **MDiR**, from −148.5 to −158.5 for **MTriRB**, and from −124.5 to −133.5 for **MH**_**3**_**PG**. Accordingly, in this case, we observe a different increasing stabilization trend of **MH**_**3**_**PG** < **MTriRB** < **MDiR**, resulting from the coformer structural and electronic adaptability order H_3_PG < DiR < TriRB discussed above and also from the presence of an additional _L_O–H···N_M–C≡N_ hydrogen bond in **MDiR** (showing in total seven hydrogen bond contacts vs. six in **MTriRB**) that naturally produces an excess of interaction energy compared to **MTriRB**. The variations in the absolute interaction energy values of all motifs in question observed across the series of compounds with DiR and TriRB coformers correlate with the increasing ionic radius of the metal and [M(CN)_6_]^3–^ anion size according to a general trend of Cr(III) < Fe(III) < Co(III). However, the energetic stabilization does not change in a perfectly linear manner, which might be attributed to the fine collective adjustment of all intermolecular contacts.

**Table 2:** DFT-Computed (B3LYP+D4//TZP) Interaction Energy Values (in kcal mol^–1^) between Hexacyanometallate Anion [M(CN)_6_]^3–^ (M = Cr, Fe, Co) and (A) Polyresorcinol DiR Molecule(s), (B) Polyresorcinol TriRB Molecule(s), and (C) Phloroglucinol H_3_PG Molecule(s) in the Molecular Clusters Extracted from the Respective Crystal Structures of **MDiR**, **MTriRB**, and **MH**_**3**_**PG**(52)^,^^d^

**A**			
	[Cr(CN)_6_]^3–^	[Fe(CN)_6_]^3–^	[Co(CN)_6_]^3–^
(**H**^**A**^**H**^**B**^DiR)_2_(**H**^**A**^_**2**_DiR)(**H**^**A**^DiR)	–163.34	–168.43	–171.23
(**H^A^H^B^**DiR) *side 2HB*	–51.07, –50.23^a^	–53.64, –50.77^a^	–54.50, –52.13^a^
**H**^**A**^_**2**_DiR *frontal 2HB*	–45.97	–47.94	–49.06

**B**				
	[Cr(CN)_6_]^3–^	[Fe(CN)_6_]^3–^	[Co^01^(CN)_6_]^3–^	[Co^02^(CN)_6_]^3–^
(**H**^**B**^**H**^**C**^TriRB)_2_(**H**TriRB)_2_	–148.82	–156.65	–158.27	–157.17
(**H**^**B**^**H**^**C**^TriRB) *side 2HB*	–54.54, –54.90^a^	–58.86, –55.72^a^	–60.00, –57.14^a^	–58.63, –57.84^a^
(**H**TriRB) *frontal 1HB*	–27.79, –29.46^a^	–b	–29.87, –30.42^a^	–31.32, –28.92^a^

**C**^c^			
	[Cr(CN)_6_]^3–^	[Fe(CN)_6_]^3–^	[Co(CN)_6_]^3–^
(**H**_**2**_PGH)_2_(**H**PGH_2_)_2_	–124.87	–133.38	–130.88
**H**_**2**_PGH *2HB*	–44.65	–47.98	–46.40
**H**PGH_2_*1HB*	–22.30	–24.29	–23.99

a Two numbers correspond to two (slightly different) motifs of the same type found in the crystal structure.

b Obtained results were considered unreliable due to significant spin-contamination in the wave function of the molecular cluster.

c Taken from ref (52).

d For cluster visualization, see Figures S11–S13 and ref (52). 2HB/1HB stands for the double/single hydrogen bonding interaction.

### IR, Raman, Mössbauer, and UV–Vis Spectra

IR and Raman spectra contain bands corresponding to molecular components and intermolecular synthons indicated by SC XRD analysis (Figures S21–23). The shape and spectral position of some absorption peaks are notably modified with respect to the reference solids, which is attributed to the enhancement of noncovalent interactions within the charge-assisted hydrogen-bond network. This holds for the bathochromic shift of *ca*. 200 cm^–1^ in the 3600–2500 cm^–1^ range of ν(O–H) vibrations of DiR and TriRB (IR spectra), the hypsochromic shift of *ca*. 20 cm^–1^ in the 2200–2000 cm^–1^ range of ν(C≡N) vibrations of [M(CN)_6_]^3–^ (IR and Raman spectra), and some shifts for the specific skeletal coformers vibrations (IR and Raman spectra).^52,61,62^

**Figure 4:**
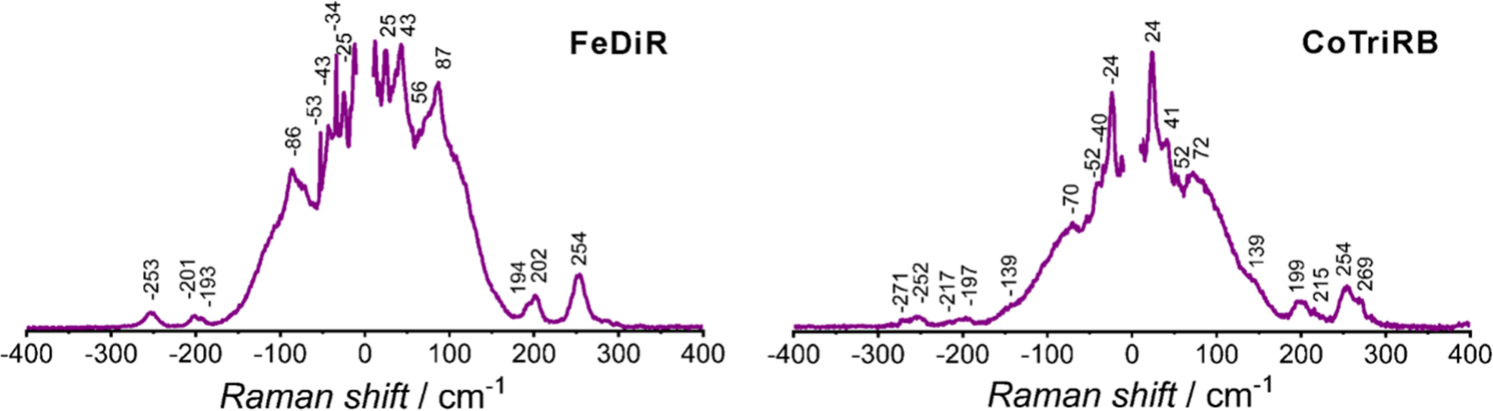
Representative low-frequency Raman scattering spectra for **FeDiR** and **CoTriRB**.

LF-Raman scattering spectra for all the presented materials exhibit numerous symmetrical LF-Raman peaks (Figures 4 and S24) with a few peaks in the sub-THz region: 25 cm^–1^ (0.75 THz) for **FeDiR**, 31 cm^–1^ (0.93 THz) for **CoDiR**, 22 cm^–1^ (0.66 THz) for **CrDiR**, 18 cm^–1^ (0.54 THz) for **CrTriRB** as well as **FeTriRB**, and 24 cm^–1^ (0.72 THz) for **CoTriRB**. A noteworthy observation is that the lowest Raman peak for the Co(III) based compounds is shifted to higher energy by 6 cm^–1^ compared to the other analogs. The low-frequency Raman peaks are associated with weak van der Waals and hydrogen bonding networks. Therefore, the observed differences in the Raman peak between the Co(III) compounds and the Fe(III) or Cr(III) analogs can be attributed to the stronger hydrogen bonding present in the former, as supported by the results of the DFT calculations (Table 2). A similar Raman shift toward higher energy was observed for [Yb^III^(TPPO)_3_(NCX)_3_] (X = S and Se) compounds but related rather to the substitution of the heavier Se atom with the lighter S one.^42^

^57^Fe Mössbauer parameters are perfectly in line with the structural data (Figures S25) and previous findings for the **MH**_**3**_**PG** series.^52^

**Figure 5:**
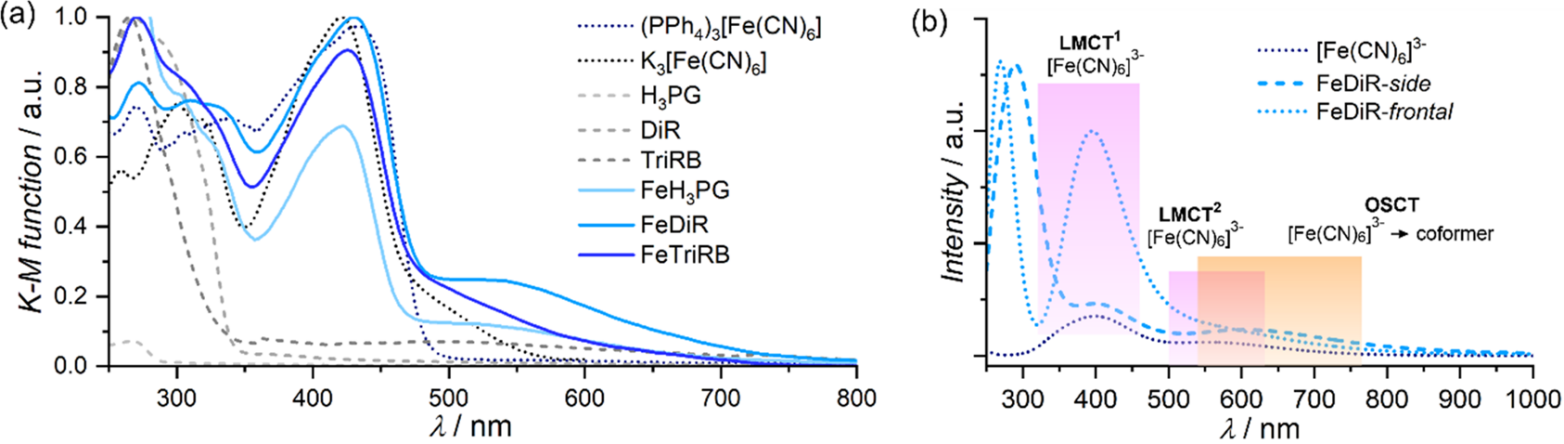
(a) Experimental UV–vis electronic absorption spectra for **FeDiR**, **FeTriRB**, and **FeH**_**3**_**PG** along with spectra for the reference salts and coformers in the solid state, in the Kubelka–Munk (K-M) form. (b) Simulated (TD-DFT PBE//TZVP) UV–vis electronic absorption spectra of the FeDiR-*side* and FeDiR-*frontal* models, representative of the fundamental hydrogen-bonded motifs observed in the crystal structures of **FeDiR**, and of the [Fe(CN)_6_]^3–^ anion as a reference. The absorption assigned to LMCT transitions within the [Fe(CN)_6_]^3–^ anion (schematically highlighted by the pink areas) is observed in all the considered cases, whereas the additional lowest-energy OSCT [Fe(CN)_6_]^3–^ → DiR transitions (the orange area) are shown for both models of **FeDiR** (for details, see the SI, Figures S27–S31).

UV–vis spectra in the solid state in the form of the Kubelka–Munk function show the spectral features of all involved components and indicate diverse absorption threshold points depending on the [M(CN)_6_]^3–^ used, in line with the results presented previously for the **MH**_**3**_**PG** networks (Figure S26).^52^ Interestingly, unlike the case of [Cr(CN)_6_]^3–^ and [Co(CN)_6_]^3–^ congeners, the spectra of all [Fe(CN)_6_]^3–^ containing phases systematically show some additional spectral features fairly distinguished within the wavelength ranges above ligand-to-metal charge-transfer (LMCT) σ(CN^–^) → π(t_2g_) transition bands (maxima between 400 and 430 nm), spread up to *ca*. 750 nm for **FeDiR**, *ca*. 700 nm for **FeTriRB**, *ca*. 700 nm for **FeH**_**3**_**PG**,^52^ and only *ca*. 580 nm for K_3_[Fe(CN)_6_], and not present for PPh_4_[Fe(CN)_6_]·7H_2_O salt (Figure 5). The TD-DFT (PBE//TZVP) UV–vis calculations and the subsequent MO-pair analysis of the computed excitations correctly reproduced the LMCT assignment of the absorption centered around 410 nm (see LMCT^1^ in Figure 5b) and more importantly indicated that the additional lowest-energy absorption for **FeDiR** (Figure 5b), **FeTriRB**, and **FeH**_**3**_**PG** might be attributed to the intermolecular optical outer-sphere charge-transfer (OSCT) [Fe(CN)_6_]^3–^ → coformer transitions through the O–H···N_complex_ hydrogen bonding orbital pathway (for details, see the section on UV–vis electronic absorption spectra in the SI, Figures S27–S31). In particular, two different **FeDiR** models, FeDiR-*side* and FeDiR-*frontal*, representing two structurally and energetically different hydrogen bonding interactions (*vide supra*), demonstrated differences in the basal discrete OSCT absorption position and in the intensity ratio *I*_LMCT_^1^/*I*_OSCT_ (Figures S29 and S30), which is in qualitative agreement with the measured spectra. In the case of the pristine [Fe(CN)_6_]^3–^ salts, the feature attributed to OSCT was not reproduced in the calculations, which remains in line with the lack of the proper orbital pathway. However, one important issue, namely, the absorption designated as LMCT^2^ appearing systematically for all the examined models in this study, requires a comment. In the relevant spectral range just above 500 nm, a very weak absorption might be observed experimentally for the [Fe(CN)_6_]^3–^ anion in the aqueous solution and in the solid state, to which the spin-forbidden ligand-field (LF) character was assigned by using the ligand-field parameter approach^63^ and, very recently, by combining CASSCF+NEVPT2 calculations with resonant inelastic X-ray scattering (RIXS).^64^ While the expected spin-forbidden LF transitions could not be directly modeled using the adopted computational protocol, their contribution might be indirectly reflected in the observed LMCT^2^ transitions (Figures S28–S31), considering a strong mixing of LF transitions with LMCT transitions found in the case of [Fe(CN)_6_]^3−^ ^64^ as well as in the case of other Fe(III) complexes.^65^ Thus, while the computational data presented herein (due to the methodological limitations and the specific character of the adopted molecular models) give a rather tentative description of UV–vis electronic spectra for FeL compounds in the solid state, it is clear that they confirm a strong impact of hydrogen bonding interactions on the valence electronic structure of **FeL**. Finally, it is worth highlighting that the described optical absorption cutoff thresholds (*ca*. 450–470 nm for the Cr(III), *ca*. 700 nm for the Fe(III), and *ca*. 375 nm for the Co(III) DiR and TriRB compounds) vividly shape the quality of the 520 nm → 1040 nm second-harmonic generation and luminescent properties of the tested samples (see below).

### SHG Studies

**Figure 6:**
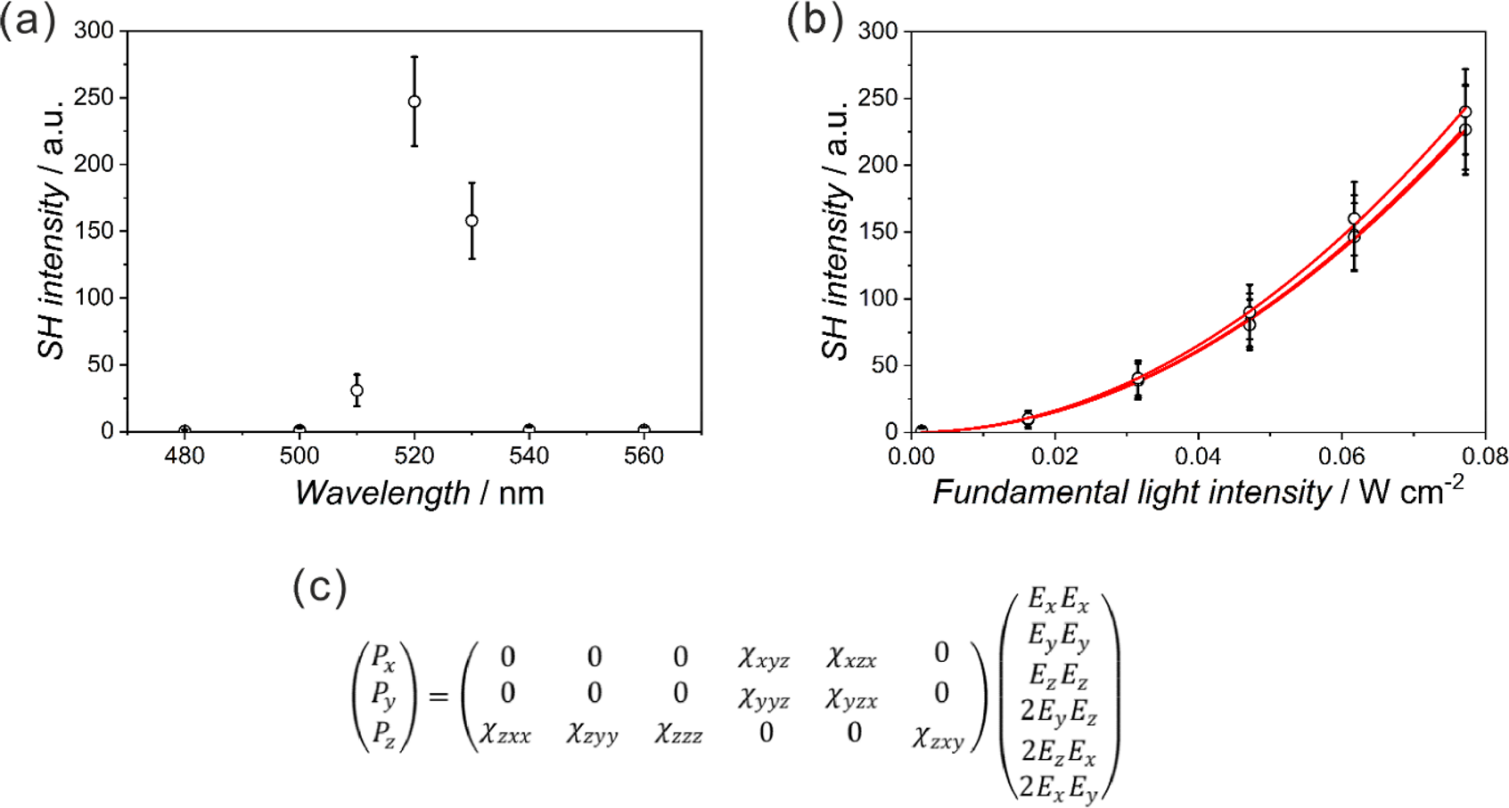
Second-harmonic signals for **CoTriRB** plotted against the wavelength to confirm the chromaticity aberration of the SH signal (a). Average SHG signals of **CoTriRB** (b); red solid lines correspond to the results of fitting using a quadratic function (*y* = *Ax*^2^). Relation between tensor elements of the SH susceptibility corresponding to space group *P*2_1_ and SH polarization (c).

Considering the noncentrosymmetric space groups found for **CrDiR**, **FeDiR**, **CoDiR** (*Pna*2_1_), **CrTriRB** (*Cc*), **FeTriRB** (*P*2_1_2_1_2), and **CoTriRB** (*P*2_1_) (Figure S1, Tables S1 and S2), their SHG response was examined for the powdered samples (Figures 6, S32, and S33). The room-temperature SHG studies were conducted in the reflectance mode using an in-house SHG setup^42^ and aimed at the observation of the 520 nm SH light (maximum of the SH light intensity vs wavelength plot) upon the 1040 nm fundamental light illumination in the reflection mode. Determined SH vs fundamental light intensity plots were fitted with the quadratic function (*y = A* × *x*^2^) and matched to the potassium dihydrogen phosphate (KDP) standard.^66^ Consequently, the SH susceptibilities (χ_SH_) of 6.0 × 10^–12^ (0.5% KDP) for **CrDiR**, 7.2 × 10^–12^ (0.6% KDP) for **FeDiR**, 2.2 × 10^–11^ (1.8% KDP) for **CoDiR**, 6.2 × 10^–11^ (5.2% KDP) for **CrTriRB**, 3.2 × 10^–11^ (2.7% KDP) for **FeTriRB**, and 1.4 × 10^–10^ (11.7% KDP) for **CoTriRB** were determined.

**Figure 7:**
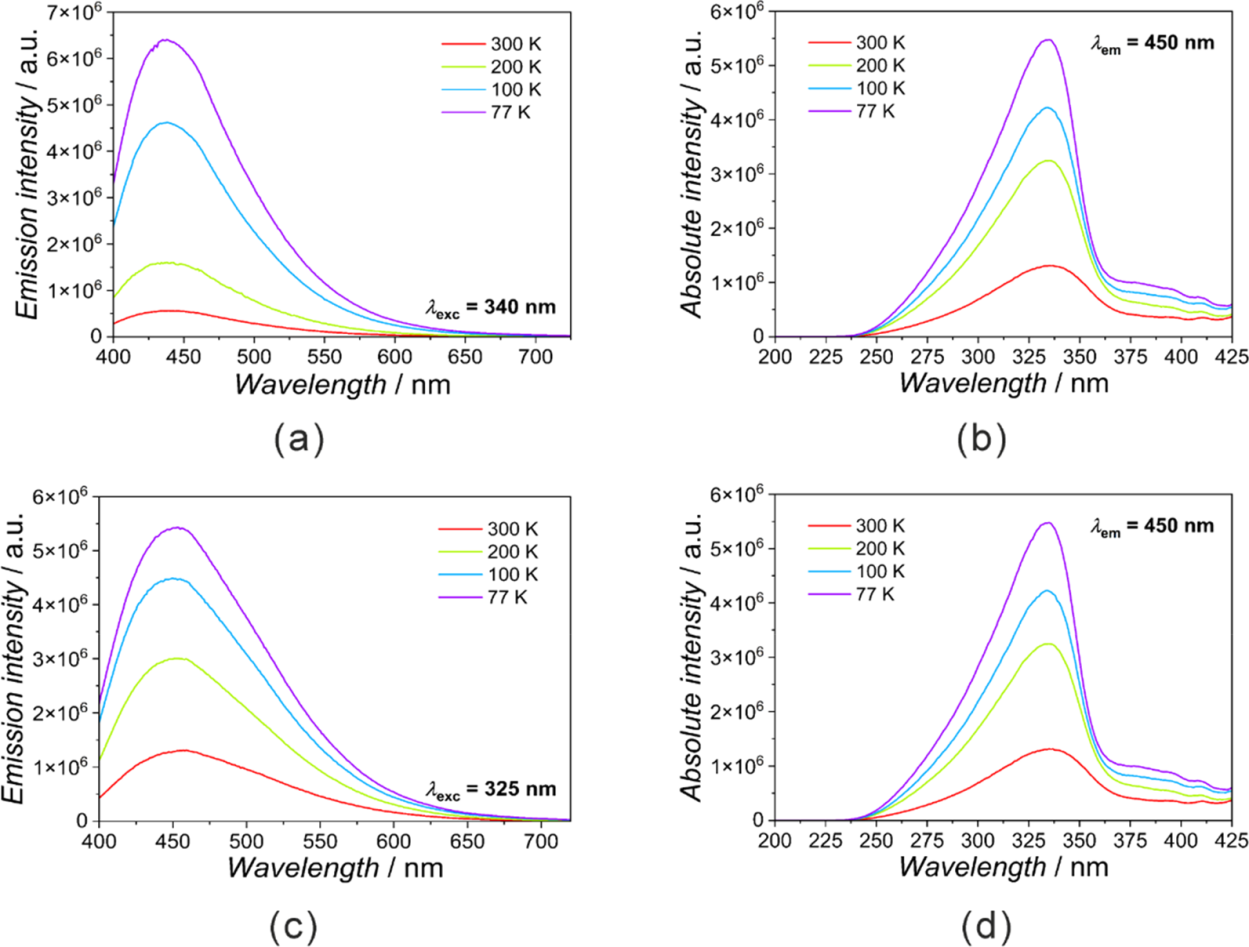
Photoluminescence spectra of **CoDiR** and **CoTriRB**. Emission spectra for the corresponding excitation wavelengths of 340 nm for **CoDiR** (a) and 325 nm for **CoTriRB** (c). Excitation spectra followed the emission wavelength of 450 nm for **CoDiR** (b) and **CoTriRB** (d).

It is worth noting that for both DiR- and TriRB-based series of samples, a tendency to increase the SHG signal value for the compounds containing Co(III) can be observed with respect to the other metal ions, which might be due to the optical transparency of **CoDiR** and **CoTriRB** in the visible region. Moreover, the values are comparable with previous reports for polycrystalline samples of cyanido-bridged assemblies (Table S15),^67−76^ and they are an outcome of nonzero elements of the second-order NLO susceptibility tensor (χ_*ijk*_) for the *P*2_1_ space group (symmetry class: 2 with polar tensor: *B*_3_) (Figure 7), for the *Cc* space group (symmetry class: m with polar tensor: *C*_3_) (Figure S33), for the *P*2_1_2_1_2 space group (symmetry class: 222 with polar tensor: *D*_3_) (Figure S33), and for the *Pna*2_1_ space group (symmetry class: mm2 with polar tensor: *E*_3_) (Figure S32). The DiR-containing materials have lower SHG efficiency than the TriRB-based cocrystals, possibly due to smaller electron polarization, which directly affects the SHG signal.

### Photoluminescent Properties

Both DiR and TriRB ligands exhibit intense blue luminescence with a maximum at around 400 nm upon excitation with light of *ca*. 325 nm (Figures S34 and S35). Furthermore, pristine DiR and TriRB showed respectively a 5- and 1.5-fold increase in the excitation and emission intensities upon cooling to 77 K (the boiling point of liquid nitrogen), accompanied by a slight fluorescence color shift from dark blue to bright blue. Spectral analysis for **CrDiR** and **CrTriRB** (Figures S36 and S37), **FeDiR** and **FeTriRB** (Figures S38 and S39), and **CoDiR** and **CoTriRB** (Figures 7, S40, and S41) revealed that the compounds based on Cr(III) and Fe(III) emit relatively weak visible light, predominantly originating from their corresponding organic coformers. On the other hand, the compounds based on Co(III) display an intense cyan luminescence due to the synergistic combination of red visible emission from Co(III) ions and bluish emission from the organic ligands. The corresponding CIE1931 uniform chromaticity scale diagrams are shown in Figure S42. It is worth emphasizing that the emission intensities for all the compounds increased upon cooling from room to cryogenic temperatures, often attributable to the suppression of nonradiative vibrational loss. Interestingly, for the **CrDiR** and **CrTriRB** samples, there are additional emission bands with two distinctive maxima at *ca*. 810 and 825 nm from the ^2^*E*_g_ → ^4^*A*_2g_ emission centered at the Cr(III) ion.^52^

Looking at the diverse features in the radiative emission of all of these compounds, we also performed quantum yield (QY) measurements for the powder samples at room temperature (Table S16). The previous findings were substantiated, confirming that pristine organic ligands exhibit very low emission quantum yields of less than 1%. The QY values for **CrDiR** and **CrTriRB** were determined to be 8.9% and 2.4%, respectively, when they were excited with 375 nm light. Similarly, the QY values for **CoDiR** and **CoTriRB** were found to be 1.7% and 1.3%, respectively, under 325 nm light irradiation. Significantly, a higher QY for the Cr(III) systems is due to the presence of near-infrared emission around 810 and 825 nm.

## Conclusion and Perspectives

Realizing the cocrystallization approach, we combined linear bis-resorcinol DiR or triangular tris-resorcinol TriRB multiple hydrogen bond donors with [M(CN)_6_]^3–^ (M = Cr, Fe, Co) hydrogen bond acceptors provided in the form of the PPh_4_^+^ salts. As expected, the resulting crystal phases showed the extended 3D hydrogen-bonded subnetworks {[M(CN)_6_]^3–^;L_*n*_}_∞_ accompanied by the subnetwork of PPh_4_^+^ stabilized by MPE interactions assisted by solvent molecules. Within the {[M(CN)_6_]^3–^;L_*n*_}_∞_ subnetworks, we recognized a range of interesting hydrogen-bonded motifs. The most elegant ones are “intuitive” (*e.g.*, expected based on the results of our previous studies involving phloroglucinol H_3_PG coformer)^52^ supramolecular *cis*-bis(chelate)-like {[M(CN)_6_]^3–^;(**H**_**2**_L)_2_(**H**L)_2_} or tris(chelate)-like {[M(CN)_6_]^3–^;(**H**_**2**_L)_3_} fragments. In the current work, they are composed of the cyclic, either *frontal* or *side* {[M(CN)_6_]^3–^;(**H**_**2**_L)}, synthons, involving phenolic groups attached to the same resorcinol-like ring of an L molecule or the different rings of one L, respectively. The image is completed with single {[M(CN)_6_]^3–^;(**H**L)} synthons as well as more complex aggregates involving more than two molecules. The quantum-chemical analysis demonstrates the mixed electrostatic and covalent character of the underlying hydrogen bonding interactions, the strength of which is clearly enhanced due to the negative charge of the hydrogen bond acceptor metal complex. The DFT-computed energy of interactions between [M(CN)_6_]^3–^ and L per representative {[M(CN)_6_]^3–^;(**H**_**2**_L)} motifs ranges from −46 to −60 kcal mol^–1^ and depends on the geometry of the contact and size matching of its components, rotational degree of freedom and extent of π-electron system of the coformer, and finally, the overall fit to the molecular surroundings. The symmetry of the crystal lattices of the obtained compounds is correlated with the effective local symmetry of coformers and {complex;(coformer)_*n*_} hydrogen-bonded motifs characterized by the absence of the inversion center and mirror plane, which is reflected by their *D*_2_, *C*_2_, or *C*_1_ point groups. It is important to note that the obtained acentric structural resolution remains in contrast with the centrosymmetric space groups observed for the native solvate crystals of DiR and TriRB. As our studies are the first to show the use of these molecules as building blocks, in the future, it would be interesting to examine the structural self-assembly in the solid state involving other molecular or ionic counterparts bearing prerequisites toward molecular functionality.

The SHG activity of our compounds is comparable to that of other polycyanidometallate-based molecular solids. Although the SHG performance is weaker than that of the KDP standard, the presented systems pave the way for a new strategy toward achieving noncentrosymmetric molecular architectures. Furthermore, as some of our crystals possess enantiopure Sohncke space groups, it would be interesting to examine crystallization of the enantiopure phases and circularly polarized photoluminescent response according to this still dynamically developing field in natural science. The interesting LF-Raman scattering spectral characteristics within the sub-THz spectroscopic domain might be further considered in the fabrication of the sub-THz response by optical stimulation. Finally, the obtained phases might be used as modular precursors toward the assembly of advanced multicomponent constructs, such as solid solutions, crystal-of-crystal arrays, and core–shell crystals, to test their optical performance. Also, the new multisite anion receptors might be designed and synthesized based on the arrangements achieved in this study. The work is in progress in our groups along the above lines.

## Materials and Methods

A description of synthetic procedures,^54^ X-ray diffraction analysis,^77−79^ structural data presentation,^80^ physicochemical techniques, and computational methods and protocols^60,81−97^ used in this study can be found in the SI.

## Supporting Information Available

Detailed description of the used materials, synthetic procedures, physical techniques, crystal structure determination, structural, computational, and physicochemical data presentation, and additional figures and tables (PDF)
