# Supplementary material for: Nonlinear and Emissive {[MIII(CN)6]3–···Polyresorcinol} (M = Fe, Co, Cr) Cocrystals Exhibiting an Ultralow Frequency Raman Response
Source: Inorg Chem. 2023 Dec 18;63(4):1803–15. doi: 10.1021/acs.inorgchem.3c03153 (PMC10828991; doi:10.1021/acs.inorgchem.3c03153)
Supplement: Supplementary file 1 — ic3c03153_si_001.pdf [file ic3c03153_si_001.pdf]

Supplementary Information

Nonlinear and Emissive {[$M^{III}(CN)_6$]$^{3-}$···Polyresorcinol} (M = Fe, Co, Cr) Cocrystals Exhibiting an Ultralow Frequency Raman Response

Katarzyna Jędrzejowska[1,2], Jedrzej Kobylarczyk[3], Dominika Tabor[1], Monika Srebro-Hooper[1], Kunal Kumar[4], Guanping Li[4], Olaf Stefanczyk[4], Tadeusz M. Muzioł[5], Katarzyna Dziedzic-Kocurek[6], Shin-ichi Ohkoshi[4]*, Robert Podgajny[1]*

[1] *Faculty of Chemistry, Jagiellonian University in Krakow, Gronostajowa 2, 30-387 Kraków, Poland; katarzyna.jedrzejowska@doctoral.uj.edu.pl (K.J.); dominika.tabor@student.uj.edu.pl (D.T.); monika.srebro@uj.edu.pl (M.S.-H.); robert.podgajny@uj.edu.pl (R.P.)*

[2] *Doctoral School of Exact and Natural Sciences, Jagiellonian University in Kraków, Prof. St. Łojasiewicza 11, 30-348 Kraków, Poland; katarzyna.jedrzejowska@doctoral.uj.edu.pl (K.J.)*

[3] *Institute of Nuclear Physics PAN, Radzikowskiego 152, 31-342 Kraków, Poland; jedrzej.kobylarczyk@ifj.edu.pl (J.K.)*

[4] *Department of Chemistry, School of Science, The University of Tokyo, 7-3-1 Hongo, Bunkyo-ku, Tokyo 113-0033, Japan; kunal-k@chem.s.u-tokyo.ac.jp (K.K.); guanping@chem.s.u-tokyo.ac.jp (G.L.); olaf@chem.s.u-tokyo.ac.jp (O.S.); ohkoshi@chem.s.u-tokyo.ac.jp (S.O.)*

[5] *Faculty of Chemistry, Nicolaus Copernicus University in Toruń, Gagarina 7, 87-100 Toruń, Poland; tmuziol@umk.pl (T.M.)*

[6] *Marian Smoluchowski Institute of Physics, Jagiellonian University, Łojasiewicza 11, 30-348 Krakow, Poland; k.dziedzic-kocurek@uj.edu.pl (K.D.-K.)*

***** *Correspondence: ohkoshi@chem.s.u-tokyo.ac.jp (S.O.), robert.podgajny@uj.edu.pl (R.P.). Tel. +48-12-686-2459 (R.P.)*

Table of contents

1. Details of structural description 6

Figure S1. Experimental room-temperature PXRD patterns of the polycrystalline samples of **MDiR** and **MTriRB** (M = Cr, Fe, and Co) in mother liquor (*RT*, exp), compared with the PXRD patterns simulated based on the respective SC XRD data.. 6

Figure S2. Asymmetric units of **MDiR** (M = Cr, Fe, and Co)... 7

Figure S3. Asymmetric units of **MTriRB** (M = Cr, Fe, and Co).. 8

Figure S4. Crystal structures of **CoDiR** and **CoTriRB**: hydrogen-bonded $\{[Co(CN)_6]^{3-};L_n\}_\infty$ subnetworks, organic cations and solvent molecules $\{PPh_4^+\}$·solv subnetwork, and complete projection. 9

Table S1. Crystal data and structure refinement of **MDiR**. 10

Table S2. Crystal data and structure refinement of **MTriRB**.. 11

Table S3. The most important bond lengths and angles for **MDiR**. 12

Table S4. The most important bond lengths and angles for **MTriRB**. 13

Table S5. Important hydrogen bonding distances and angles in $_L$O-H···$N_{M\text{-}C\equiv N}$ subnetworks of **MDiR**. 14

Table S6. Important hydrogen bonding distances and angles in $_L$O-H···$N_{M\text{-}C\equiv N}$ subnetworks of **CrTriRB** and **FeTriRB**. 14

Table S7. Important hydrogen bonding distances and angles in $_L$O-H···$N_{M\text{-}C\equiv N}$ subnetworks of **CoTriRB**. 15

Table S8. Distances and angles for X-H···O (X = C, O) hydrogen bonds in **MDiR**............................. 16

Table S9. Distances and angles for C-H···O hydrogen bonds in **MTriRB**. 17

Figure S5. ESI-MS spectra in the negative ionization mode for **FeDiR** and for **FeTriRB**................... 18

Figure S6. ESI-MS spectra in the positive ionization mode for **FeDiR** and for **FeTriRB**.................... 19

Table S10. Interplanar angles formed by twisting the aromatic rings in **MDiR** and **MTriRB** and visualization of the degree of rings twist in the described DiR and TriRB molecules 20

Figure S7. Schematic illustration of the interplanar angle α in DiR including the ring designation A and B and visualization of three orthogonal rotation axes C_2 producing the D_2 symmetry point group 20

Figure S8. Schematic illustration of the interplanar angles α, β, and γ in TriRB including the ring designation A, B, C, and D and visualization of the symmetry element for the C_2 or C_1 symmetry point group... 21

Figure S9. Two 3D projections of the *dmp* topology of hydrogen-bonded $\{[M(CN)_6]^{3-};DiR_2\}_\infty$ (M = Cr, Fe, Co) subnetworks in **MDiR** architectures along the selected mutually orthogonal directions. ... 23

Figure S10. The *neb* topology of hydrogen-bonded $\{[Cr(CN)_6]^{3-};TriRB\}_\infty$ subnetwork in CrTriRB and the *dia* topology of hydrogen-bonded $\{[Co(CN)_6]^{3-};TriRB\}_\infty$ subnetwork in CoTriRB 23

2. Quantum-chemical studies on hydrogen bonding interactions 24

2.1. Computational details.. 24

Figure S11. Visualization of the examined molecular clusters {$[M(CN)_6]^{3-}$;(**H**^{A}**H**^{B}DiR)$_2$(**H**$^A{}_2$DiR)(**H**^{A}DiR)}, {$[M(CN)_6]^{3-}$;(**H**^{A}**H**^{B}DiR)}, {$[M(CN)_6]^{3-}$;(**H**$^A{}_2$DiR)}, {$[M(CN)_6]^{3-}$;(**H**^{A}DiR)}, {$[M(CN)_6]^{3-}$;(**H**$^A{}_2$DiR)(**H**^{A}DiR)}, and {(DiR);(**H**^{A}DiR)} extracted from the crystal structures of **MDiR** with M = Cr, Fe, and Co. 25

Figure S12. Visualization of the examined molecular clusters {$[M(CN)_6]^{3-}$;(**H**^{B}**H**^{C}TriRB)$_2$(**H**TriRB)$_2$}, {$[M(CN)_6]^{3-}$;(**H**^{B}**H**^{C}TriRB)}, and {$[M(CN)_6]^{3-}$;(**H**TriRB)} extracted from the crystal structures of **MTriRB** with M = Fe and Co........ 26

Figure S13. Visualization of the examined molecular clusters {$[Cr(CN)_6]^{3-}$;(**H**^{B}**H**^{C}TriRB)$_2$(**H**TriRB)$_2$}, {$[Cr(CN)_6]^{3-}$;(**H**^{B}**H**^{C}TriRB)}, and {$[Cr(CN)_6]^{3-}$;(**H**TriRB)} extracted from the crystal structure of **CrTriRB**. 26

2.2. Calculated data 27

Table S11. DFT-computed (B3LYP+D4//TZP, BLYP+D4//TZP in parentheses) interaction energy values (in kcal/mol) between hexacyanometallate anion $[M(CN)_6]^{3-}$ (M = Cr, Fe, and Co) and polyresorcinol DiR molecule(s) in molecular clusters {$[M(CN)_6]^{3-}$;(**H**^{A}**H**^{B}DiR)$_2$(**H**$^A{}_2$DiR)(**H**^{A}DiR)}, {$[M(CN)_6]^{3-}$;(**H**^{A}**H**^{B}DiR)}, {$[M(CN)_6]^{3-}$;(**H**$^A{}_2$DiR)(**H**^{A}DiR)}, {$[M(CN)_6]^{3-}$;(**H**$^A{}_2$DiR)}, and {$[M(CN)_6]^{3-}$;(**H**^{A}DiR)}, and between the DiR molecules in {(DiR);(**H**^{A}DiR)} motif, extracted from the crystal structures of **MDiR** with M = Cr, Fe, and Co........ 28

Table S12. Hydrogen bond acceptor ($[Co(CN)_6]^{3-}$ or DiR) / hydrogen bond donor (DiR) energy interaction values and their components (in kcal/mol) according to the ETS energy decomposition scheme for the molecular clusters {$[Co(CN)_6]^{3-}$;(**H**^{A}**H**^{B}DiR)}, {$[Co(CN)_6]^{3-}$;(**H**$^A{}_2$DiR)(**H**^{A}DiR)}, {$[Co(CN)_6]^{3-}$;(**H**$^A{}_2$DiR)}, {$[Co(CN)_6]^{3-}$;(**H**^{A}DiR)}, and {(DiR);(**H**^{A}DiR)} extracted from the crystal structures of **MDiR** with M = Cr, Fe, and Co........ 29

Figure S14. ETS-NOCV analysis of the interaction between $[Co(CN)_6]^{3-}$ and DiR molecule in the molecular cluster {$[Co(CN)_6]^{3-}$;(**H**^{A}**H**^{B}DiR)}-I extracted from the crystal structure of **CoDiR**........ 30

Figure S15. ETS-NOCV analysis of the interaction between $[Co(CN)_6]^{3-}$ and DiR molecules in the molecular cluster {$[Co(CN)_6]^{3-}$;(**H**$^A{}_2$DiR)(**H**^{A}DiR)} extracted from the crystal structure of **CoDiR**...... 31

Figure S16. ETS-NOCV analysis of the interaction between $[Co(CN)_6]^{3-}$ and DiR molecule in the molecular cluster {$[Co(CN)_6]^{3-}$;(**H**$^A{}_2$DiR)} extracted from the crystal structure of **CoDiR**........ 32

Figure S17. ETS-NOCV analysis of the interaction between $[Co(CN)_6]^{3-}$ and DiR molecule in the molecular cluster {$[Co(CN)_6]^{3-}$;(**H**^{A}DiR)} extracted from the crystal structure of **CoDiR**........ 33

Figure S18. ETS-NOCV analysis of the interaction between two DiR molecules in the molecular cluster {(DiR);(**H**^{A}DiR)} extracted from the crystal structure of **CrDiR**. 34

Table S13. DFT-computed (B3LYP+D4//TZP, BLYP+D4//TZP in parentheses, values obtained with B3LYP+D3//TZVP using Gaussian 16 indicated by the superscript G16) interaction energy values (in kcal/mol) between hexacyanometallate anion $[M(CN)_6]^{3-}$ (M = Cr, Fe, Co) and polyresorcinol TriRB molecule(s) in molecular clusters {$[M(CN)_6]^{3-}$;(**H**^{B}**H**^{C}TriRB)$_2$(**H**TriRB)$_2$}, {$[M(CN)_6]^{3-}$;(**H**^{B}**H**^{C}TriRB)}, and {$[M(CN)_6]^{3-}$;(**H**TriRB)} extracted from the crystal structures of **MTriRB** with M = Cr, Fe, and Co..... 35

Table S14. Hydrogen bond acceptor ($[Co(CN)_6]^{3-}$) / hydrogen bond donor (TriRB) energy interaction values and their components (in kcal/mol) according to the ETS energy decomposition scheme for the molecular clusters {$[Co(CN)_6]^{3-}$;(**H**^{B}**H**^{C}TriRB)} and {$[Co(CN)_6]^{3-}$;(**H**TriRB)} extracted from the crystal structure of **CoTriRB**. 36

Figure S19. ETS-NOCV analysis of the interaction between $[Co(CN)_6]^{3-}$ and TriRB molecule in the molecular cluster {$[Co^{01}(CN)_6]^{3-}$;(**H**^{B}**H**^{C}TriRB)}-I extracted from the crystal structure of **CoTriRB**. ... 37

Figure S20. ETS-NOCV analysis of the interaction between $[Co(CN)_6]^{3-}$ and TriRB molecule in the molecular cluster {$[Co^{02}(CN)_6]^{3-}$;(**H**TriRB)}-I extracted from the crystal structure of **CoTriRB**. 38

3. Details of spectroscopic characterization 39

3.1. Vibrational studies 39

Figure S21. Room-temperature IR spectra for the single crystals of **CrDiR**, **FeDiR**, **CoDiR**, **CrTriRB**, **FeTriRB**, and **CoTriRB** measured in a wide range. 39

3.2. Room-temperature Raman scattering spectroscopy 39

Figure S22. Infrared spectra of **MDiR** and **MTriRB** in the absorption mode, including the regions characteristic of the *v*(O-H) and *v*(C≡N) vibrations, and the skeletal vibrations in the fingerprint region 39

Figure S23. Room-temperature Raman scattering spectra for the single crystals of **MDiR** and **MTriRB** measured in a wide range. 40

3.3. Low-frequency (LF) Raman scattering spectroscopy 41

Figure S24. Room-temperature LF-Raman scattering spectra for the single crystals of **CrDiR**, **CoDiR**, **CrTriRB**, and **FeTriRB**. 41

3.4. ^{57}Fe Mössbauer spectra 41

Figure S25. ^{57}Fe Mössbauer spectra of **FeDiR** and of **FeTriRB**. 41

3.5. UV-Vis electronic absorption spectra 41

Figure S26. Solid-state UV-Vis absorption spectra of the **MDiR** and **MTriRB** compounds in the range of 250-800 nm together with those of DiR, TriRB and $PPh_4[M(CN)_6]\cdot nH_2O$ precursors as the references; M = Cr, Fe, and Co. 42

Figure S27. Molecular models used in calculations of UV-Vis electronic spectra representative of $[Fe(CN)_6]^{3-}$ references, **FeH$_3$PG**, and **FeDiR**. 43

Figure S28. Simulated UV-Vis spectrum for the pristine $[Fe(CN)_6]^{3-}$ model 44

Figure S29. Simulated UV-Vis spectrum for the FeDiR-*side* model. 44

Figure S30. Simulated UV-Vis spectrum for the FeDiR-*frontal* model. 45

Figure S31. Simulated UV-Vis spectrum for the FeH$_3$PG-*frontal* model 45

4.6. Second-harmonic generation studies 46

Figure S32. Second-harmonic signals for **CrDiR**, **FeDiR**, and **CoDiR**. 46

Figure S33. Second-harmonic signals for **CrTriRB**, and **FeTriRB**. 47

Table S15. Room-temperature second-harmonic susceptibilities for powdered samples of **MDiR** and **MTriRB**, the KDP standard, and other reference assemblies. 48

4.7. Photoluminescence studies 50

Figure S34. Photoluminescence spectra of DiR. 50

Figure S35. Photoluminescence spectra of TriRB. 50

Figure S36. Photoluminescence spectra of **CrDiR**. 51

Figure S37. Photoluminescence spectra of **CrTriRB**. 51

Figure S38. Photoluminescence spectra of **FeDiR**. 52

Figure S39. Photoluminescence spectra of **FeTriRB**.. 52
Figure S40. Photoluminescence spectra of **CoDiR**. 52
Figure S41. Photoluminescence spectra of **CoTriRB**. 52
Figure S42. CIE1931 uniform chromaticity scale diagrams for DiR and TriRB ligands, **CrDiR**, **CrTriRB**, **FeDiR**, **FeTriRB**, **CoDiR**, and **CoTriRB**. 53
Table S16. Values of the measured quantum yield (QY) for **CrDiR**, **CrTriRB**, **CoDiR**, and **CoTriRB** together with the corresponding data for the coformers DiR and TriRB as the references. 54
4.8. Details of materials and methods 55
4.9. Thermogravimetric analysis 57
Figure S43. Thermogravimetric curves of **MDiR** and **MTriRB** 57
References to Supplementary Information 59

1. Details of structural description

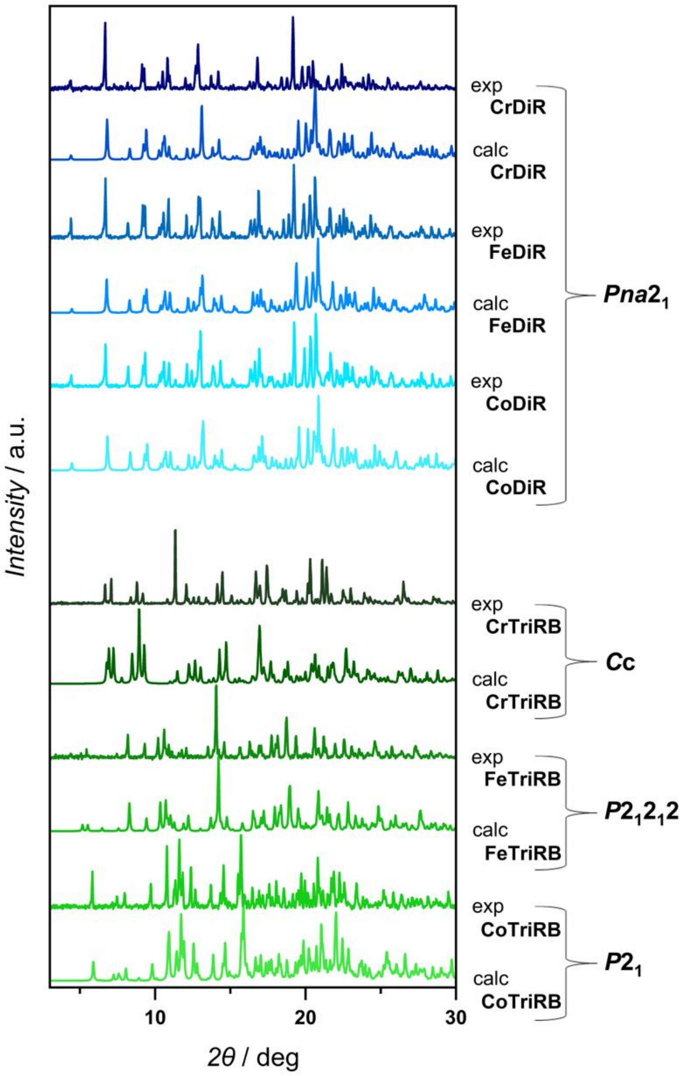

Figure S1. Experimental room-temperature PXRD patterns of the polycrystalline samples of MDiR and MTriRB (M = Cr, Fe, and Co) in mother liquor (*RT*, exp), compared with the PXRD patterns simulated based on the respective SC XRD data (*T* = 100 K, calc).

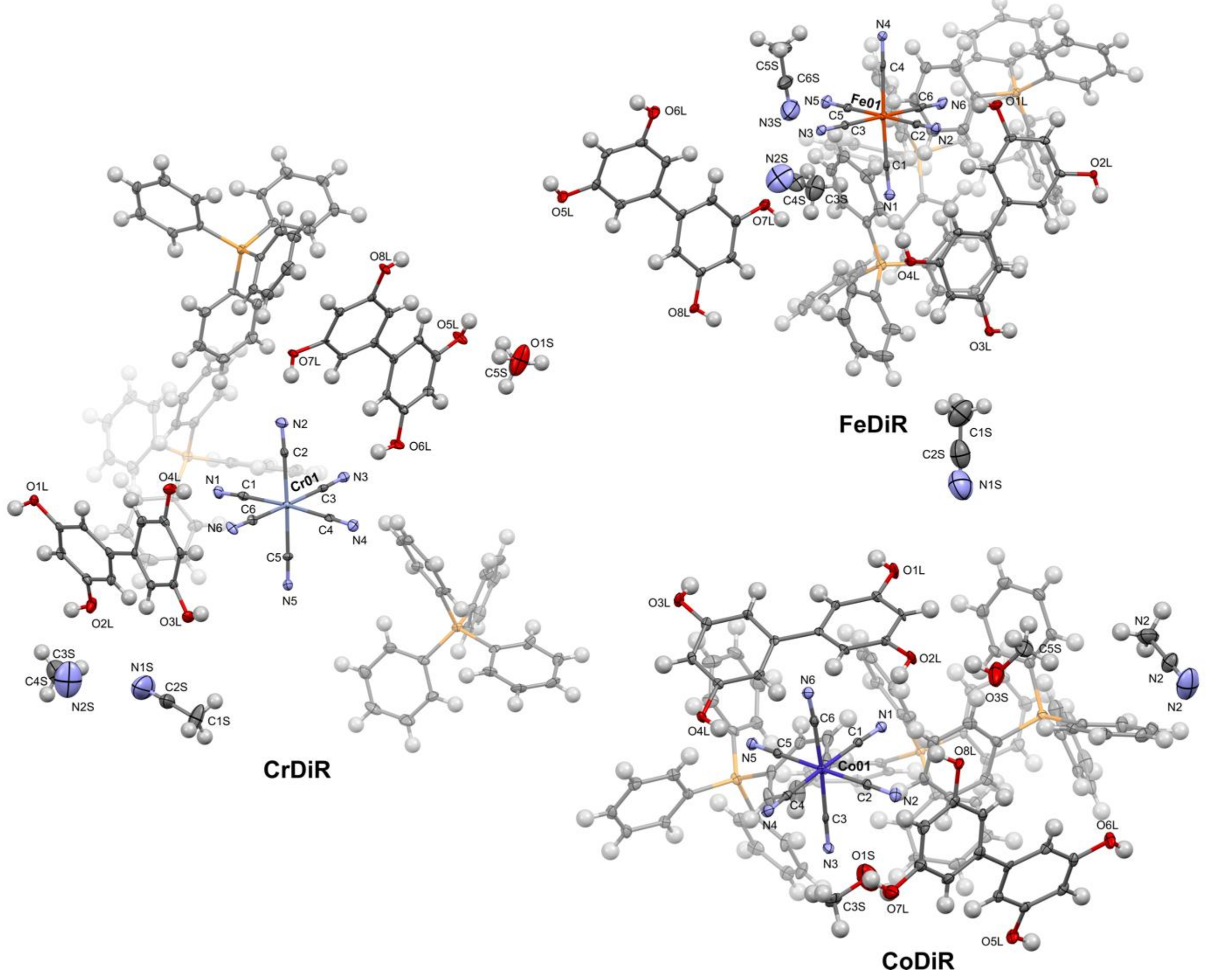

Figure S2. Asymmetric units of **MDiR** (M = Cr, Fe, and Co). Colors: light blue – Cr, dark orange – Fe, dark blue – Co, grey – C, blue – N, red – O, orange – P, light grey – H. The thermal ellipsoids are drawn at the 50% probability level.

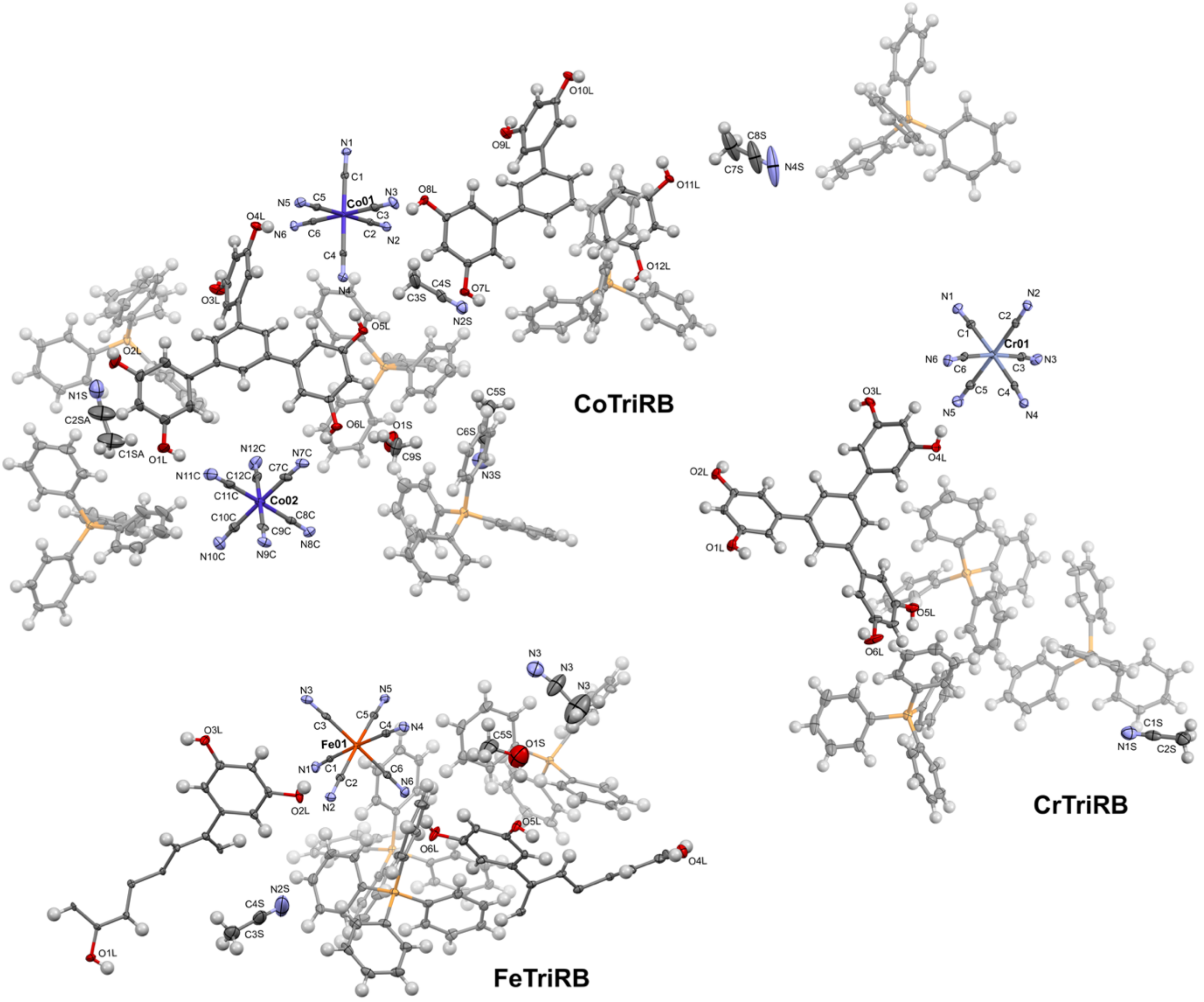

Figure S3. Asymmetric units of **MTriRB** (M = Cr, Fe, and Co). Colors: light blue – Cr, dark orange – Fe, dark blue – Co, grey – C, blue – N, red – O, orange – P, light grey – H. The thermal ellipsoids are drawn at the 50% probability level.

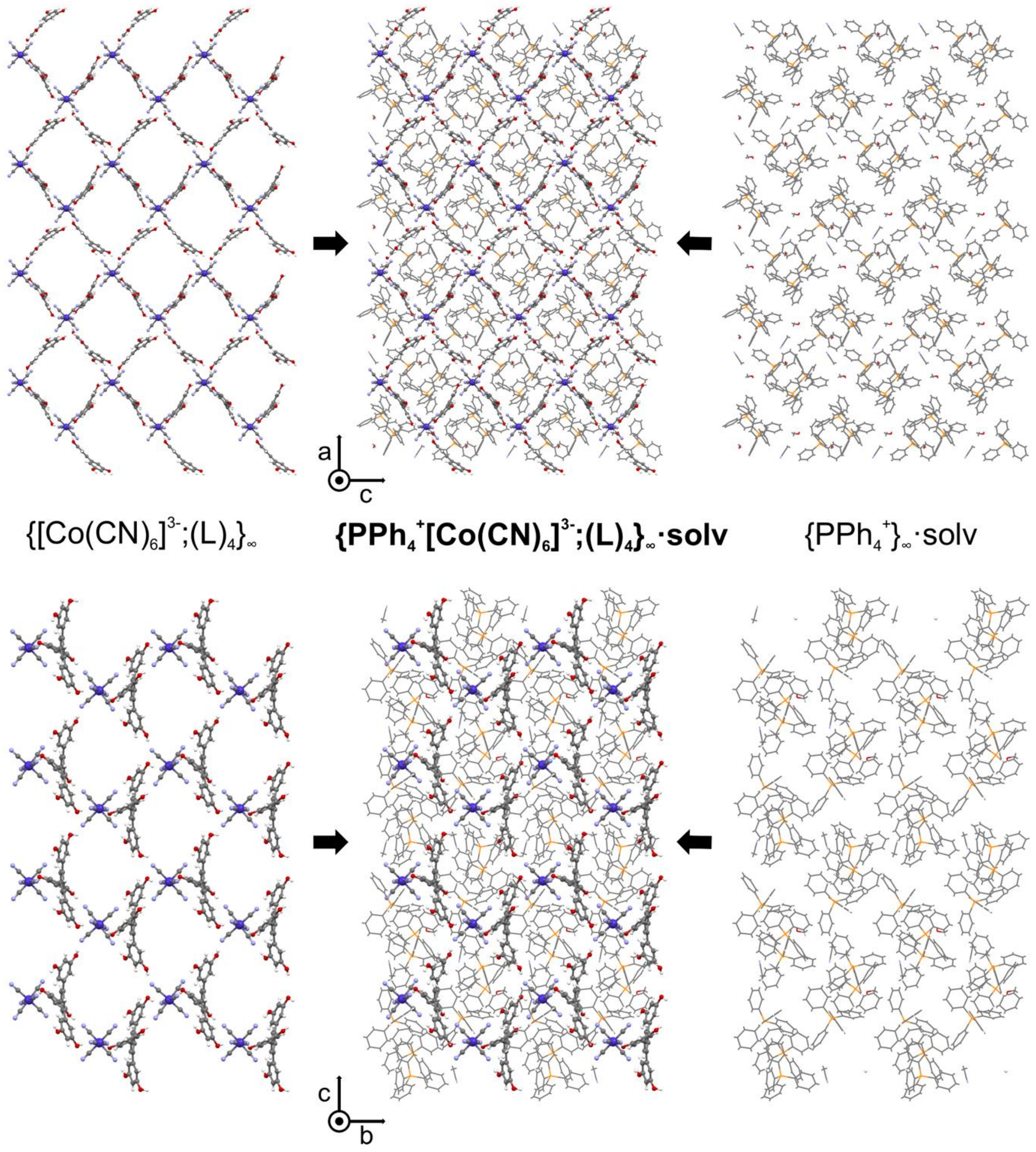

Figure S4. Crystal structures of **CoDiR** (top; projection along the *b* crystallographic direction) and **CoTriRB** (bottom; projection along the *a* crystallographic direction): hydrogen-bonded $\{[Co(CN)_6]^{3-};L_n\}_\infty$ subnetworks (*left panel*), organic cations and solvent molecules $\{PPh_4^+\}\cdot$solv subnetwork (*right panel*), and complete projection (*middle panel*).

Table S1. Crystal data and structure refinement of **MDiR**.

**Compound**		**CrDiR**	**FeDiR**	**CoDiR**
Crystal data				
formula		$C_{107}H_{90}CrN_8O_9P_3$	$C_{108}H_{89}FeN_9O_8P_3$	$C_{106}H_{91}CoN_7O_{10}P_3$
formula weight / g·mol^{-1}		1776.77	1789.64	1774.69
T / K		100.0		
λ / Å		0.71073 (MoKα)		
crystal system		orthorhombic		
space group		Pna2$_1$		
cell parameters	a / Å	40.1199(11)	39.6424(11)	39.5914(10)
	b / Å	13.7205(5)	13.8089(4)	13.6914(3)
	c / Å	16.8199(5)	16.7336(5)	16.6951(4)
	α, β, γ / deg	90		
	V / Å^3	9258.8(5)	9160.3(5)	9049.8(4)
Z		4		
ρ_{calc} / g·cm^{-3}		1.275	1.298	1.303
μ / cm^{-1}		0.240	0.282	0.308
F(000)		3716.0	3740.0	3712.0
crystal type		colorless plate	pale yellow plate	colourless plate
crystal size / mm × mm × mm		0.5 × 0.18 × 0.15	0.37 × 0.12 × 0.09	0.61 × 0.19 × 0.15
Data collection				
2θ range / deg		5.03 to 50.054	4.776 to 50.054	4.932 to 51.36
limiting indices		$-47 \leq h \leq 47$<br>$-16 \leq k \leq 16$<br>$-20 \leq l \leq 19$	$-47 \leq h \leq 47$<br>$-16 \leq k \leq 16$<br>$-19 \leq l \leq 19$	$-48 \leq h \leq 48$<br>$-16 \leq k \leq 16$<br>$-20 \leq l \leq 20$
collected reflections		81023	79649	74856
independent reflections		16006 [R_{sigma} = 0.0534]	16055 [R_{sigma} = 0.0519]	17083 [R_{sigma} = 0.0505]
R_{int}		0.0615	0.0588	0.0512
Refinement				
data/restraints/parameters		16006/1/1165	16055/7/1173	17083/33/1183
GOF on F^2		1.176	1.115	1.146
final R indices		R_1 = 0.0546 [I ≥2σ(I)]<br>wR_2 = 0.1058 (all data)	R_1 = 0.0480 [I ≥2σ(I)]<br>wR_2 = 0.0985 (all data)	R_1 = 0.0486 [I ≥2σ(I)]<br>wR_2 = 0.0968 (all data)
largest diff. peak/hole / e·Å^{-3}		0.41/-0.41	0.40/-0.39	0.38/-0.40
Flack x-parameter		0.024(9)	0.018(7)	0.025(5)

Table S2. Crystal data and structure refinement of **MTriRB**.

Compound		CrTriRB	FeTriRB	CoTriRB
Crystal data				
formula		$C_{107}CrH_{93}N_7O_9P_3$	$C_{107}H_{88}FeN_8O_7P_3$	$C_{213}H_{172}Co_2N_{16}O_{13}P_6$
formula weight / g·mol^{-1}		1765.79	1746.61	3467.34
T / K		100.0		
λ / Å		0.71073 (MoKα)		
crystal system		monoclinic	orthorhombic	monoclinic
space group		*Cc*	$P2_12_12$	$P2_1$
cell parameters	a / Å	28.5867(7)	19.7524(6)	16.7094(4)
	b / Å	15.4118(4)	27.2819(8)	18.0176(5)
	c / Å	24.4014(10)	17.0927(5)	30.2050(7)
	α / deg	90	90	90
	β / deg	121.2300(10)	90	96.8650(10)
	γ / deg	90	90	90
	V / Å^3	9192.7(5)	9211.0(5)	9028.4(4)
Z		4		2
ρ_{calc} / g·cm^{-3}		1.276	1.260	1.275
μ / cm^{-1}		0.241	0.277	0.305
F(000)		3700.0	3652.0	3620.0
crystal type		colourless plate	yellow plate	colourless plate
crystal size / mm × mm × mm		0.6 × 0.27 × 0.22	0.4 × 0.15 × 0.1	0.42 × 0.32 × 0.11
Data collection				
2θ range / deg		5.066 to 50.054	4.386 to 52.044	4.498 to 50.054
limiting indices		$-34 \leq h \leq 34$<br>$-18 \leq k \leq 18$<br>$-29 \leq l \leq 29$	$-24 \leq h \leq 24$<br>$-33 \leq k \leq 33$<br>$-21 \leq l \leq 21$	$-19 \leq h \leq 19$<br>$-21 \leq k \leq 21$<br>$-35 \leq l \leq 31$
collected reflections		60397	122720	74347
independent reflections		16199 [R_{sigma} = 0.0314]	18148 [R_{sigma} = 0.0346]	31771 [R_{sigma} = 0.0580]
R_{int}		0.0276	0.0461	0.0330
Refinement				
data/restraints/parameters		16199/2/1097	18148/57/1149	31771/72/2354
GOF on F^2		1.059	1.110	1.050
final R indices		R_1 = 0.0308 [I ≥2σ(I)]<br>wR_2 = 0.0702 (all data)	R_1 = 0.0469 [I ≥2σ(I)]<br>wR_2 = 0.1133 (all data)	R_1 = 0.0465 [I ≥2σ(I)]<br>wR_2 = 0.1018 (all data)
largest diff. peak/hole / e·Å^{-3}		0.28/-0.27	0.90/-0.43	0.73/-0.41
Flack x-parameter		0.014(4)	0.016(4)	0.019(4)

Table S3. The most important bond lengths (in Å) and angles (in deg) for **MDiR**. Detailed metric parameters of hexacyanidometallate anions.

compound	**CrDiR**	**FeDiR**	**CoDiR**
M =	**Cr**	**Fe**	**Co**
M–C	2.077(5)	1.933(5)	1.896(4)
	2.055(5)	1.936(5)	1.898(5)
	2.064(5)	1.934(5)	1.894(5)
	2.060(5)	1.938(5)	1.909(4)
	2.070(5)	1.945(5)	1.894(4)
	2.066(5)	1.940(5)	1.901(5)
C≡N	1.142(6)	1.151(6)	1.152(5)
	1.147(6)	1.148(6)	1.154(6)
	1.147(6)	1.154(6)	1.149(6)
	1.153(6)	1.153(6)	1.133(6)
	1.153(6)	1.148(6)	1.147(6)
	1.148(6)	1.145(6)	1.142(6)
M–C≡N	177.1(4)	177.5(4)	177.5(4)
	175.1(5)	178.5(4)	176.8(4)
	175.1(5)	177.4(4)	178.2(4)
	178.8(5)	178.4(4)	176.7(4)
	177.5(4)	177.9(4)	177.7(4)
	177.3(4)	177.7(5)	176.7(4)

Table S4. The most important bond lengths (in Å) and angles (in deg) for **MTriRB**. Detailed metric parameters of hexacyanidometallate anions.

compound	**CrTriRB**	**FeTriRB**	**CoTriRB**[a]	
M =	**Cr**	**Fe**	**Co^{01}**	**Co^{02}**
M–C	2.079(3)	1.934(4)	1.910(4)	1.881(5)
	2.083(3)	1.945(4)	1.899(5)	1.901(5)
	2.068(3)	1.949(4)	1.895(4)	1.901(5)
	2.074(3)	1.935(4)	1.894(4)	1.896(5)
	2.061(3)	1.941(4)	1.907(5)	1.891(5)
	2.069(3)	1.929(4)	1.891(4)	1.894(5)
C≡N	1.142(4)	1.151(5)	1.148(5)	1.153(6)
	1.148(4)	1.157(5)	1.142(6)	1.139(6)
	1.148(4)	1.163(5)	1.140(5)	1.144(6)
	1.149(3)	1.148(5)	1.147(5)	1.144(6)
	1.147(4)	1.156(5)	1.136(5)	1.146(6)
	1.144(4)	1.155(5)	1.143(5)	1.148(6)
M–C≡N	177.2(3)	176.3(3)	177.8(4)	177.4(4)
	178.1(2)	178.6(3)	177.7(4)	179.0(4)
	175.6(3)	177.5(3)	178.9(4)	179.3(5)
	174.2(2)	177.9(4)	178.3(4)	177.9(4)
	178.4(3)	175.8(3)	178.6(4)	177.3(5)
	173.7(3)	178.5(4)	178.3(4)	177.6(5)

[a] Two crystallographically independent $[Co(CN)_6]^{3-}$ complexes were described.

Table S5. Important hydrogen bonding distances (in Å) and angles (in deg) in $_{L}$O-H···$N_{M\text{-}C\equiv N}$ subnetworks of **MDiR**.[a]

	CrDiR				FeDiR				CoDiR			
	D–H···A	D···A /Å	H···A /Å	D–H···A /deg	D–H···A	D···A /Å	H···A /Å	D–H···A /deg	D–H···A	D···A /Å	H···A /Å	D–H···A /deg
side synthon	O5L–H5L···N5	2.775	1.941	172.3	O1L–H1L···N6	2.741	1.901	176.6	O1L–H1L···N6	2.736	1.902	174.8
	O8L–H8LA···N4	2.693	1.870	165.9	O4L–H4L···N1	2.715	1.896	164.5	O3L–H3L···N4	2.740	1.917	178.7
	O7L–H7L···N2	2.710	1.874	172.8	O3L–H3L···N3	2.830	2.005	167.1	O2L–H2L···N1	2.801	1.982	164.6
	O6L–H6L···N3	2.704	1.883	165.3	O2L–H2L···N2	2.724	1.933	156.5	O4L–H4L···N5	2.724	1.908	163.2
**av.**		**2.72**	**1.89**	**169.1**		**2.75**	**1.93**	**166.1**		**2.75**	**1.93**	**170.3**
frontal synthon	O1L–H1L···N5	2.874	2.051	166.4	O7L–H7L···N4	2.754	1.915	177.0	O7L–H7L···N3	2.739	1.900	176.6
	O2L–H2L···N6	2.766	1.927	177.5	O8L–H8L···N3	2.828	1.991	174.4	O8L–H8L···N1	2.877	2.040	174.2
	O4L–H4L···N1	2.731	1.896	172.0	O5L–H5L···N5	2.751	1.925	167.5	O6L–H6L···N2	2.731	1.897	172.0
**av.**		**2.79**	**1.96**	**172.0**		**2.78**	**1.94**	**173.0**		**2.78**	**1.95**	**174.2**

[a] D – hydrogen bond donor L = DiR; A – hydrogen bond acceptor $[M(CN)_6]^{3-}$.

Table S6. Important hydrogen bonding distances (in Å) and angles (in deg) in $_{L}$O-H···$N_{M\text{-}C\equiv N}$ subnetworks of **CrTriRB** and **FeTriRB**. [a]

	CrTriRB				FeTriRB			
	D–H···A	D···A /Å	H···A /Å	D–H···A /deg	D–H···A	D···A /Å	H···A /Å	D–H···A /deg
side synthon	O1L–H1L···N1	2.785	1.950	172.6	O1L–H1L···N4	2.700	1.861	176.6
	O6L–H6L···N3	2.717	1.916	159.1	O3L–H3L···N2	2.779	1.942	173.9
	O2L–H2L···N2	2.742	1.904	174.4	O4L–H4L···N3	2.978	2.145	171.5
	O3L–H3L···N4	2.850	2.018	170.9	O5L–H5L···N5	2.725	1.885	177.4
**av.**		**2.77**	**1.95**	**169.2**		**2.80**	**1.96**	**174.9**
frontal synthon	O4L–H4L···N5	2.664	1.856	160.9	O6L–H6LA···N6	2.684	1.852	170.7
	O5L–H5L···N6	2.714	1.880	171.4	O2L–H2L···N1	2.730	1.890	178.1
**av.**		**2.69**	**1.87**	**166.2**		**2.71**	**1.87**	**174.4**

[a] D – hydrogen bond donor L = TriRB; A – hydrogen bond acceptor $[M(CN)_6]^{3-}$.

Table S7. Important hydrogen bonding distances (in Å) and angles (in deg) in $_{L}O\text{-}H\cdots N_{M\text{-}C\equiv N}$ subnetworks of **CoTriRB**.

	**CoTriRB** [a]							
	**Co**^{01}				**Co**^{02}			
	D–H···A	D···A /Å	H···A /Å	D–H···A /deg	D–H···A	D···A /Å	H···A /Å	D–H···A /deg
side synthon	O4L–H4L···N5C	2.716	1.879	174.9	O1L–H1L···N11C	2.638	1.803	172.7
	O5L–H5L···N4C	2.719	1.886	170.8	O6L–H6L···N7C	2.625	1.791	171.8
	O7L–H7L···N2C	2.683	1.845	175.6	O10L–H10A···N8C	2.741	1.901	179.6
	O12L–H12A···N6C	2.685	1.848	173.8	O11L–H11L···N12C	2.713	1.882	169.7
**av.**		**2.70**	**1.86**	**173.8**		**2.69**	**1.84**	**173.5**
frontal synthon	O8L–H8LA···N3C	2.684	1.850	171.7	O3L–H3L···N9C	2.694	1.862	170.4
	O9L–H9L···N1C	2.776	1.945	169.9	O2L–H2L···N10C	2.736	1.919	164.0
**av.**		**2.73**	**1.90**	**170.8**		**2.71**	**1.89**	**167.2**

[a] There are two different $[Co(CN)_6]^{3-}$ anions in the asymmetric unit, hence separate hydrogen bonds for **Co**^{01} and **Co**^{02}. D – hydrogen bond donor L = TriRB; A – hydrogen bond acceptor $[M(CN)_6]^{3-}$.

Table S8. Distances (in Å) and angles (in deg) for X-H···O (X = C, O) hydrogen bonds in **MDiR**.[a]

**CrDiR**				**FeDiR**				**CoDiR**			
O···H–X	O···X /Å	O···H /Å	X–H···O /deg	O···H–X	O···X /Å	O···H /Å	X–H···O /deg	O···H–X	O···X /Å	O···H /Å	X–H···O /deg
O1L···H3L–O3L	2.783	1.967	164.0	O1L···H21K–C21K	3.504	2.672	146.5	O2L···H3S–O3S	2.859	2.044	163.1
O2L···H3SB–C3S	3.178	2.597	118.1	O2L···H2K–C2K	3.401	2.557	148.3	O3L···H35K–C35K	3.486	2.656	146.3
O2L···H70K–C70K	3.234	2.427	142.7	O5L···H66K–C66K	3.465	2.571	156.9	O3L···H5SA–C5S	3.370	2.527	144.2
O3L···H4K–C4K	3.454	2.582	152.7	O6L···H46K–C46K	3.384	2.527	150.3	O4L···H38K–C38K	3.402	2.547	149.9
O3L···H53K–C53K	3.412	2.599	143.7	O6L···H71K–C71K	3.482	2.675	143.1	O5L···H21K–C21K	3.476	2.684	141.3
O4L···H72K–C72K	3.487	2.589	158.0	O7L···H64K–C64K	3.227	2.437	140.5	O5L···H58K–C58K	3.322	2.546	153.4
O5L···H1S–O1S	2.850	2.048	159.7	O8L···H6L–O6L	2.715	1.903	162.2	O6L···H2K–C2K	3.438	2.539	158.2
O7L···H5SC–C5S	3.460	2.599	146.7	–	–	–	–	O7L···H4K–C4K	3.272	2.449	144.8
O8L···H26K–C26K	3.377	2.520	150.2	–	–	–	–	O8L···H5L–O5L	2.783	1.993	156.5
O1S···H54K–C54K	3.321	2.571	136.0	–	–	–	–	O3S···H14K–C14K	3.188	2.424	137.3
O1S···H62K–C62K	3.231	2.446	139.9	–	–	–	–	O3S···H20K–C20K	3.284	2.546	134.7

[a] L, K, S, SA, SB, SC are part of the designations of the individual atoms that build the crystal lattice, see Figure S2 and crystallographic files. Hydrogen atoms have been omitted for the sake of clarity.

Table S9. Distances (in Å) and angles (in deg) for C-H···O hydrogen bonds in **MTriRB**.[a]

**CrTriRB**				**FeTriRB**				**CoTriRB**			
O···H–X	O···X /Å	O···H /Å	X–H···O /deg	O···H–X	O···X /Å	O···H /Å	X–H···O /deg	O···H–X	O···X /Å	O···H /Å	X–H···O /deg
O2L···H6K–C6K	3.251	2.544	131.3	O1L···H62K–C62K	3.339	2.453	155.1	O1L···H38–C38	3.126	2.435	129.3
								O2L···H4A–C4A	3.059	2.596	110.3
O3L···H16K–C16K	3.311	2.505	142.7	O2L···H64K–C64K	3.528	2.626	157.5	O2L···H106–C106	3.290	2.572	132.6
O4L···H47K–C47K	3.485	2.591	156.8	O3L···H6K–C6K	3.528	2.628	158.2	O3L···H102–C102	3.608	2.666	171.7
O4L···H70K–C70K	3.206	2.525	128.8	O5L···H1S–O1S	2.997	2.170	168.1	O3L···H6A–C6A	2.988	2.302	128.6
O5L···H20K–C20K	3.269	2.333	168.3	O6L···H54K–C54K	3.144	2.314	145.6	O4L···H132–C132	3.439	2.518	163.3
O6L···H2SC–C2S	3.574	2.604	170.5	–	–	–	–	O5L···H64–C64	3.211	2.634	119.6
O6L···H72K–C72K	3.166	2.412	136.0	–	–	–	–	O6L···H144–C144	3.591	2.708	154.7
–	–	–	–	–	–	–	–	O6L···H1S–O1S	2.694	1.899	157.3
–	–	–	–	–	–	–	–	O6L···H9SA–C9S	3.126	2.703	106.5
–	–	–	–	–	–	–	–	O7L···H86–C86	3.211	2.428	139.6
–	–	–	–	–	–	–	–	O8L···H130–C130	3.468	2.555	161.3
–	–	–	–	–	–	–	–	O8L···H5SB–C5S	3.476	2.715	134.8
–	–	–	–	–	–	–	–	O11L···H7SA–C7S	3.310	2.380	158.2
–	–	–	–	–	–	–	–	O11L···H10B–C10A	3.269	2.554	132.2
–	–	–	–	–	–	–	–	O12L···H104–C104	3.156	2.490	127.1
–	–	–	–	–	–	–	–	O12L···H21A–C21A	3.580	2.687	157.2
–	–	–	–	–	–	–	–	O1S···H56–C56	3.087	2.539	116.9
–	–	–	–	–	–	–	–	O1S···H89–C89	3.233	2.619	122.7
–	–	–	–	–	–	–	–	O1S···H90–C90	3.236	2.631	122.0

[a] L, K, S, SA, SB, SC are part of the designations of the individual atoms that build the crystal lattice, see Figure S3 and crystallographic files. Hydrogen atoms have been omitted for the sake of clarity.

The representative ESI-MS spectrograms measured for MeOH solutions of **FeDiR** and **FeTriRB** crystals in the negative ionization mode exhibit a mono-negative peak-set (*m/z* = 890.22$^-$) assignable to the salt-like $\{(PPh_4)_2[Fe(CN)_6]\}^-$ aggregates, which are, however, accompanied by two progressive peak-sets attributable to cocrystal salt-like $\{(PPh_4)_2[Fe(CN)_6](DiR)\}^-$ (*m/z* = 1108.27$^-$) and $\{(PPh_4)_2[Fe(CN)_6](DiR)_2\}^-$ (*m/z* = 1326.35$^-$) aggregates for **FeDiR**, or $\{(PPh_4)_2[Fe(CN)_6](TriRB)\}^-$ (*m/z* = 1292.32$^-$) and $\{(PPh_4)_2[Fe(CN)_6](TriRB)_2\}^-$ (*m/z* = 1694.42$^-$) aggregates for **FeTriRB** (Figure S5). The positive ionization mode revealed the peak-set (*m/z* = 1565.48$^+$) representing $\{(PPh_4)_4[Fe(CN)_6]\}^+$ aggregates in both cases, accompanied (among others) by $\{(PPh_4)_4[Fe(CN)_6](DiR)\}^+$ (*m/z* = 1691.49$^+$) and $\{(PPh_4)_4[Fe(CN)_6](TriRB)\}^+$ (*m/z* = 1917.44$^+$) peak-sets for **FeDiR** and **FeTriRB**, respectively (Figure S6). All spectrograms confirm a strong preference for the formation of aggregates of the building blocks in the gas phase, including hydrogen-bonded ones, in line with the crystallographic and computed data.

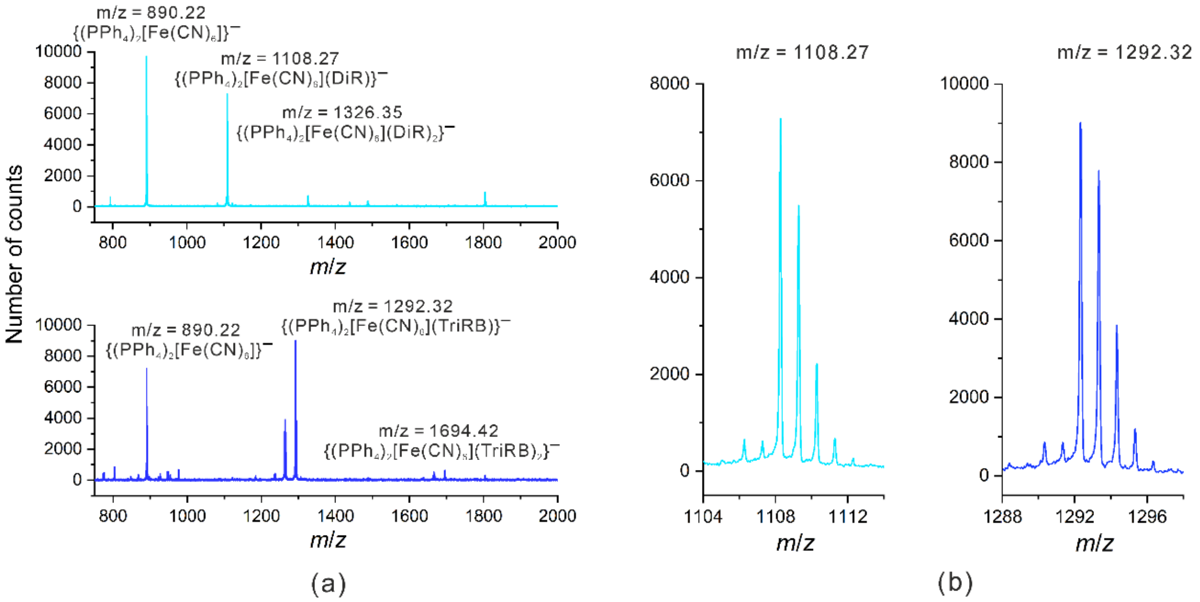

Figure S5. ESI-MS spectra in the negative ionization mode for **FeDiR** (cyan) and for **FeTriRB** (blue): (a) *m/z* range between 750$^-$ and 2000$^-$ showing the peak-sets assigned to $\{(PPh_4)_2[Fe(CN)_6](DiR)_n\}^-$, and $\{(PPh_4)_2[Fe(CN)_6](TriRB)_n\}^-$ aggregates (n = 0, 1, 2); (b) representative details of the relevant isotopic patterns. All patterns were fairly reproducible using the EnviPat software.[S1]

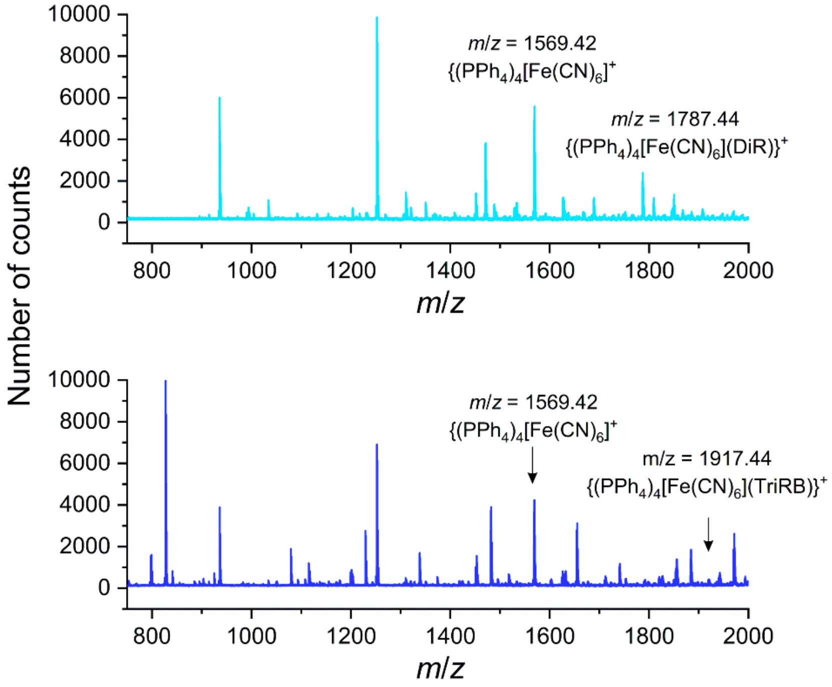

Figure S6. ESI-MS spectra in the positive ionization mode for **FeDiR** (cyan, top) and for **FeTriRB** (blue, bottom).

Table S10. Interplanar angles (in deg) formed by twisting the aromatic rings in **MDiR** and **MTriRB** (left) and visualization of the degree of rings twist in the described DiR and TriRB molecules (right). For further illustration, see **Figures S7** and **S8**.

		A–B	A–C	A–D	B–C	B–D	C–D
	**SIJFEY**	0.00	–	–	–	–	–
Cr	DiR*side*	21.9	–	–	–	–	–
	DiR*frontal*	21.4					
Fe	DiR*side*	17.2					
	DiR*frontal*	26.5					
Co	DiR*side*	23.4					
	DiR*frontal*	19.1					
	**SJGAV**	34.6	42.6	43.7	32.7	67.1	45.8
Cr	TriRB	39.9	45.2	47.3	31.6	49.3	77.7
Fe	^{1}TriRB	44.9	40.6	40.6	42.5	42.5	71.7
	^{2}TriRB	51.2	49.8	49.8	51.4	51.4	89.6
Co	^{1}TriRB	34.9	57.8	33.9	50.1	30.6	78.4
	^{2}TriRB	36.4	43.4	43.1	40.8	36.9	74.2

DiR*side* is a coformer molecule containing C1L to C12L carbon atoms, while DiR*frontal* comprises C13L to C24L atoms. The coformers in **FeTriRB** and **CoTriRB** contain C1L to C15L, C16L to C29L, and C1L to C24L and C25L to C48L, carbon atoms, respectively.

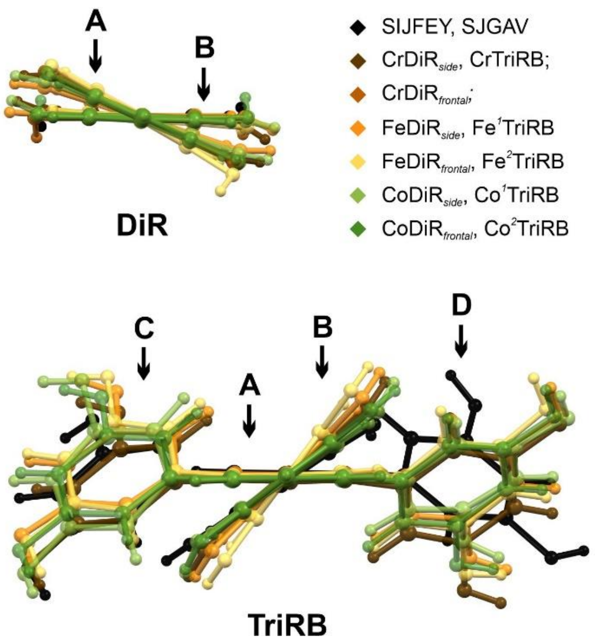

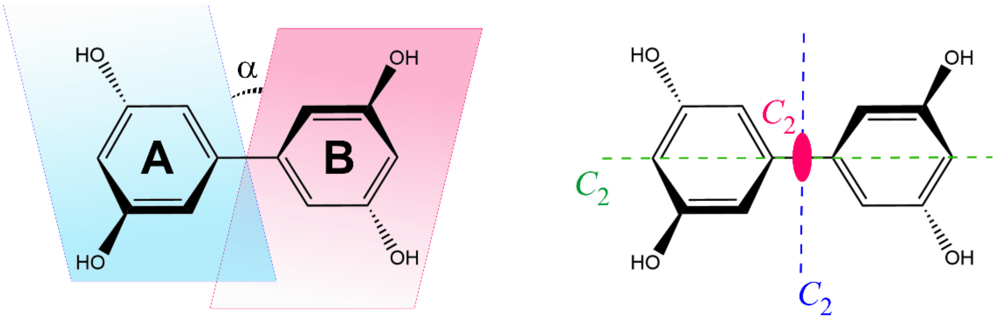

$0 < \alpha < 90^{\circ}$

D_2 symmetry

Figure S7. Schematic illustration of the interplanar angle α in DiR including the ring designation A and B (left) and visualization of three orthogonal rotation axes C_2 producing the D_2 symmetry point group (right). For the values of α, see the upper section of **Table S10**. The positions of the phenolic protons and the resulting orientation of O-H bonds with respect to the ring were disregarded.

(a)

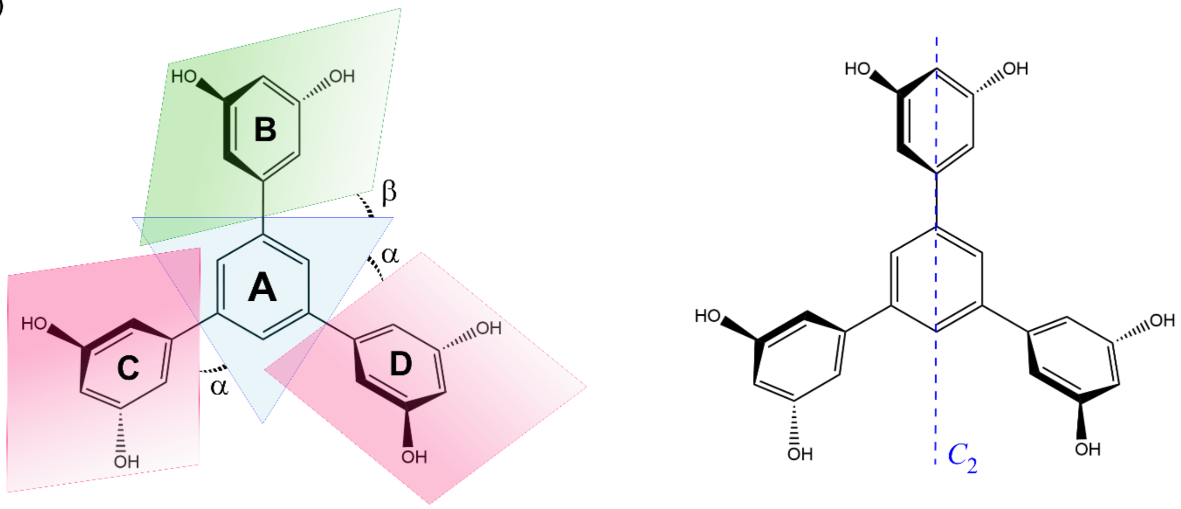

$0 < \alpha, \beta < 90^o$

$\alpha \neq \beta$

C_2 symmetry

(b)

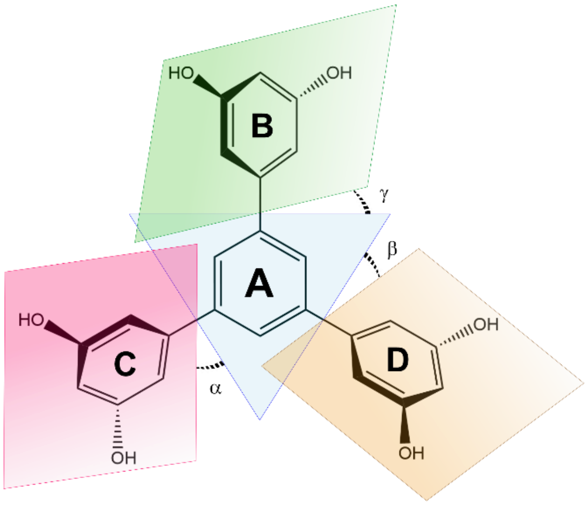

no symmetry elements other then E

$0 < \alpha, \beta, \gamma < 90^o$

$\alpha \neq \beta \neq \gamma$

C_1 symmetry

Figure S8. Schematic illustration of the interplanar angles α, β, and γ in TriRB (left) including the ring designation A, B, C, and D (left) and visualization of the symmetry element for the C_2 or C_1 symmetry point group (right): (a) *C_2* symmetry point group in **FeTriRB** and (b) *C_1* symmetry point group in **CrTriRB** and **CoTriRB**. For the values of α, β, and γ, see the bottom section of **Table S10**. The positions of the phenolic protons and the resulting orientation of the O-H bonds with respect to the ring were disregarded.

Discussion of the role of PPh_4^+ cation in the occurrence of noncentrosymmetric space groups.

A statistical approach might shed some light on that issue. Therefore, we screened the CSD database for the occurrence of XPh_4^+-containing (X = P, As) noncentrosymmetric space groups and, further, similar Sohncke groups allowing enantiopurity. We have found that out of 4523 of such crystal structures (with R_{int} not exceeding 10) 543 (12%) were noncentrosymmetric, of which 251 (5.54%) belonged to Sohncke space groups. Such statistical results indicate that XPh_4^+ cations cannot provide the simple key to achieve a noncentrosymmetric solution. We also extracted the percentage representation of "our" space groups over the total number of 543 noncentrosymmetric space groups: $P2_1$ (87, 16.0%), Cc (55, 10.2%) $Pna2_1$ (39, 7.18%) and $P2_12_12$ (12 2.21%). These results make them take first, fourth, sixth, and tenth place, respectively, among the 10 space groups exceeding 10 records (418 records in total). This suggests that within this study we (statistically) hit a target of 193 (35.5%) noncentrosymmetric space groups. The dataset shown below illustrates the results of CSD screening involving other cations with the tetrahedral core, e.g. some tetraalkylammonium cations offering various lengths and flexibility of long alkyl chains.

R_4N^+ cations

22.4% (597 per the total number of 2661 crystal structures) for tetramethylammonium cation,

0% (0 per the total number of 3759 crystal structures) for tetraethylammonium cation,

30.8% (134 per the total number of 435 crystal structures) for tetrapropylammonium cation,

16.2% (1023 per the total number of 6042 crystal structures) for tetrabutylammonium cation,

$R^1 3R^2N^+$ cations

39.7% (37 per the total number of 93 crystal structures) for tripropylmethylammonium cation,

21.4% (3 per the total number of 14 crystal structures) for triethylmethylammonium cation.

The above statistical data confirm that the PPh_4^+ cation would not rather be considered an efficient component to contribute to the induction of the noncentrosymmetric space group, compared to other cations that reveal *ca.* two-fold or even three-fold larger number of noncentrosymmetric space groups. However, we are aware that this simple approach does not consider the role of other components in all crystal structures. Other networks involving more extended cations might also be examined, and such screening might be performed following successful future studies.

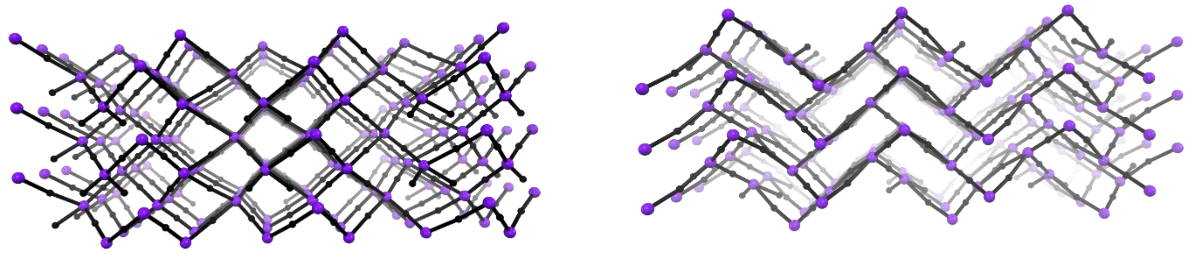

Figure S9. Two 3D projections of the dmp topology of hydrogen-bonded $\{[M(CN)_6]^{3-};DiR_2\}_\infty$ (M = Cr, Fe, Co) subnetworks in **MDiR** architectures along the selected mutually orthogonal directions. Legend: purple spheres – M sites representing $[M(CN)_6]^{3-}$ anions; black spheres – centroids of the C-C bond between the DiR phenyl rings representing coformers; black sticks – hydrogen-bonded synthons. Note that $[M(CN)_6]^{3-}$ anions are 4-connected nodes, whereas DiR molecules are linkers.

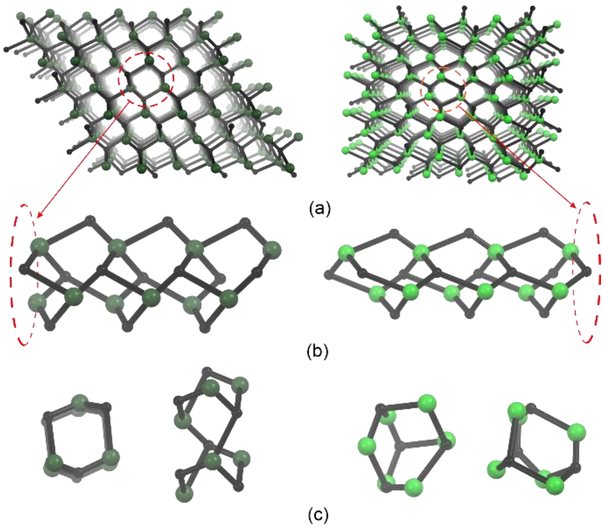

Figure S10. The *neb* topology of hydrogen-bonded $\{[Cr(CN)_6]^{3-};TriRB\}_\infty$ subnetwork in **CrTriRB** *(left panel)* and the *dia* topology of hydrogen-bonded $\{[Co(CN)_6]^{3-};TriRB\}_\infty$ subnetwork in **CoTriRB** (*right panel*): (a) selected 3D projections; (b) side views of selected 1D arrangements; (c) two projections of relevant 6^6 "cage" in **CrTriRB** and 6^4 "cage" in **CoTriRB**. Legend: dark / light green spheres – Cr / Co sites representing $[Cr(CN)_6]^{3-}$ / $[Co(CN)_6]^{3-}$ anions; black spheres – centroids of the central ring of TriRB representing coformers; black sticks – hydrogen-bonded synthons. Note that both $[M(CN)_6]^{3-}$ anions and TriRB coformers are 4-connected nodes. The images for **CoTriRB** (*right panel*) are also representative of **FeTriRB**, despite the differences in the space groups.

2. Quantum-chemical studies on hydrogen bonding interactions

2.1. Computational details

Calculations were performed for hydrogen-bonded molecular clusters consisting of the hexacyanidometallate anion $[M(CN)_6]^{3-}$ (M = Cr, Fe, Co) and polyresorcinol molecule(s) DiR ({$[M(CN)_6]^{3-}$;(**H**^{A}**H**^{B}DiR)$_2$(**H**$^{A}_{2}$DiR)(**H**^{A}DiR)}, {$[M(CN)_6]^{3-}$;(**H**^{A}**H**^{B}DiR)}, {$[M(CN)_6]^{3-}$;(**H**$^{A}_{2}$DiR)(**H**^{A}DiR)}, {$[M(CN)_6]^{3-}$;(**H**$^{A}_{2}$DiR)} and {$[M(CN)_6]^{3-}$;(**H**^{A}DiR)}) or TriRB ({$[M(CN)_6]^{3-}$;(**H**^{B}**H**^{C}TriRB)$_2$(**H**TriRB)$_2$}, {$[M(CN)_6]^{3-}$;(**H**^{B}**H**^{C}TriRB)} and {$[M(CN)_6]^{3-}$;(**H**TriRB)}), extracted from the crystal structures of **MDiR** and **MTriRB**, respectively. For the former compounds, the {(DiR);(**H**^{A}DiR)} motif with hydrogen bonding interaction between two polyresorcinol DiR molecules was also studied. All the molecular clusters listed above are presented in **Figures S11-S13**. The corresponding computed data for the {$[M(CN)_6]^{3-}$;(**H**$_2$PGH)$_2$(**H**PGH$_2$)$_2$}, {$[M(CN)_6]^{3-}$;**H**$_2$PGH}, and {$[M(CN)_6]^{3-}$;**H**PGH$_2$} (M = Cr, Fe, Co; PGH_3 = phloroglucinol) molecular clusters, presented in the main text, were taken from Reference S2.

The computations were mainly carried out using the Amsterdam Density Functional (ADF) program, version 2019.304[S3,S4], at the density functional theory (DFT) level with the fourth-generation Grimme's set of semiempirical dispersion corrections (D4),[S5] DFT+D4, employing the global hybrid B3LYP[S6-S8] and gradient BLYP[S7,S9] exchange-correlation density functionals. The calculations utilized a relativistic all-electron triple-ξ singly polarized (TZP) basis set from the ADF library. Scalar relativistic effects were modeled by using the zeroth-order regular approximation (ZORA).[S10,S11] Note that for several motifs SCF convergence and/or spin contamination problems were encountered during the calculations using the BLYP functional, and consequently, the analysis was based on the B3LYP results. See Reference S2 for a study of the dependence of computed interaction energy values on the basis set and density functional used in the calculations, which was recently performed for the aforementioned $[M(CN)_6]^{3-}$/phloroglucinol motifs. As the ADF B3LYP calculations for the {$[Fe(CN)_6]^{3-}$;(**H**TriRB)} cluster always led to a spin-contaminated wavefunction, for all the {$[M(CN)_6]^{3-}$;(**H**TriRB)} motifs additional computations were performed using the Gaussian 16 program, version C.01,[S12] employing the B3LYP functional with the third-generation Grimme's set of semiempirical dispersion corrections with the Becke-Johnson damping (D3)[S13,S14] along with a triple-ξ valence basis set augmented by polarization functions (def2-TZVP).[S15,S16]

The interaction energy between the hydrogen bond acceptor ($[M(CN)_6]^{3-}$ with a spin-quartet d^3 electronic configuration of Cr, spin-doublet d^5 of Fe, and closed-shell d^6 of Co; DiR) and the hydrogen bond donor (DiR; TriRB) molecule(s) in the examined clusters was calculated as the difference between the electronic ground-state energy of the given molecular cluster and the sum of the electronic ground-state energies of its fragments (hydrogen bond acceptor and hydrogen bond donor molecule(s)) in the geometry of the cluster. For the closed-shell motifs ($[Co(CN)_6]^{3-}$/DiR, DiR/DiR, $[Co(CN)_6]^{3-}$/TriRB), ETS-NOCV[S17,S18] charge and energy decomposition analyses were carried out, the methodological details of which are presented briefly below.

Using the extended transition state (ETS) approach, the interaction energy, ΔE_{int}, between the fragments in the geometry of the system can be divided into the following components:

$$\Delta E_{int} = \Delta E_{orb} + \Delta E_{Pauli} + \Delta E_{elstat} \left(+\Delta E_{disp}\right)$$

where:

ΔE_{orb} – corresponds to the stabilizing interactions between the occupied molecular orbitals on one fragment with the unoccupied molecular orbitals of the other fragment (describing inter-fragment donation and back-donation charge transfers), as well as the mixing of occupied and virtual orbitals within the same fragment (describing intra-fragment polarization, charge redistribution),

ΔE_{Pauli} – accounts for the repulsive Pauli interaction between occupied orbitals on the two fragments,

ΔE_{elstat} – represents the classical electrostatic interaction between the fragments in the combined system,

ΔE_{disp} – corresponds to the dispersion interaction between the fragments in the combined system, and herein was obtained at the DFT+D4 level.

The natural orbitals for chemical valence (NOCV) are eigenvectors that diagonalize the deformation density matrix in the basis of fragment orbitals. The NOCV pairs (ψ_{-k} and ψ_k with eigenvalues respectively $-v_k$ and v_k, that is of the same absolute value but opposite signs) decompose the deformation density $\Delta\rho$ into NOCV contributions $\Delta\rho_k$:

$$\Delta\rho = \sum_k \Delta\rho_k = \sum_k v_k(-\psi_{-k}^2 + \psi_k^2)$$

with k going over the pairs of NOCV's. The plots of the NOCV contributions $\Delta\rho_k$ can be analyzed visually to assign symmetry and the direction of the density flow.

Finally, within the ETS-NOCV scheme, the orbital interaction term ΔE_{orb} is further expressed in the NOCV representation as a sum of the orbital energy contributions ΔE_k corresponding to the particular $\Delta\rho_k$ channels.

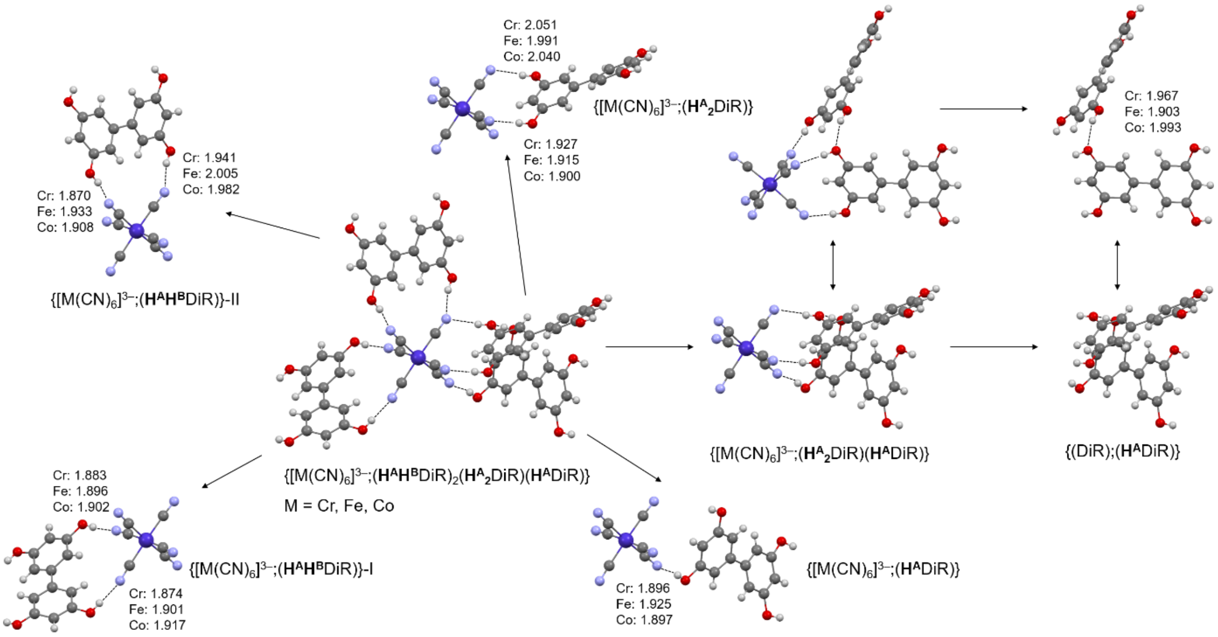

Figure S11. Visualization of the examined molecular clusters {[M(CN)$_6$]$^{3-}$;(**H**$^{\mathbf{A}}$**H**$^{\mathbf{B}}$DiR)$_2$(**H**$^{\mathbf{A}}$$_2$DiR)(**H**$^{\mathbf{A}}$DiR)}, {[M(CN)$_6$]$^{3-}$;(**H**$^{\mathbf{A}}$**H**$^{\mathbf{B}}$DiR)}, {[M(CN)$_6$]$^{3-}$;(**H**$^{\mathbf{A}}$$_2$DiR)}, {[M(CN)$_6$]$^{3-}$;(**H**$^{\mathbf{A}}$DiR)}, {[M(CN)$_6$]$^{3-}$;(**H**$^{\mathbf{A}}$$_2$DiR)(**H**$^{\mathbf{A}}$DiR)} (shown in two orientations), and {(DiR);(**H**$^{\mathbf{A}}$DiR)} (shown in two orientations) extracted from the crystal structures of **MDiR** with M = Cr, Fe, and Co. Numbers listed are O–H···N distances, in Å.

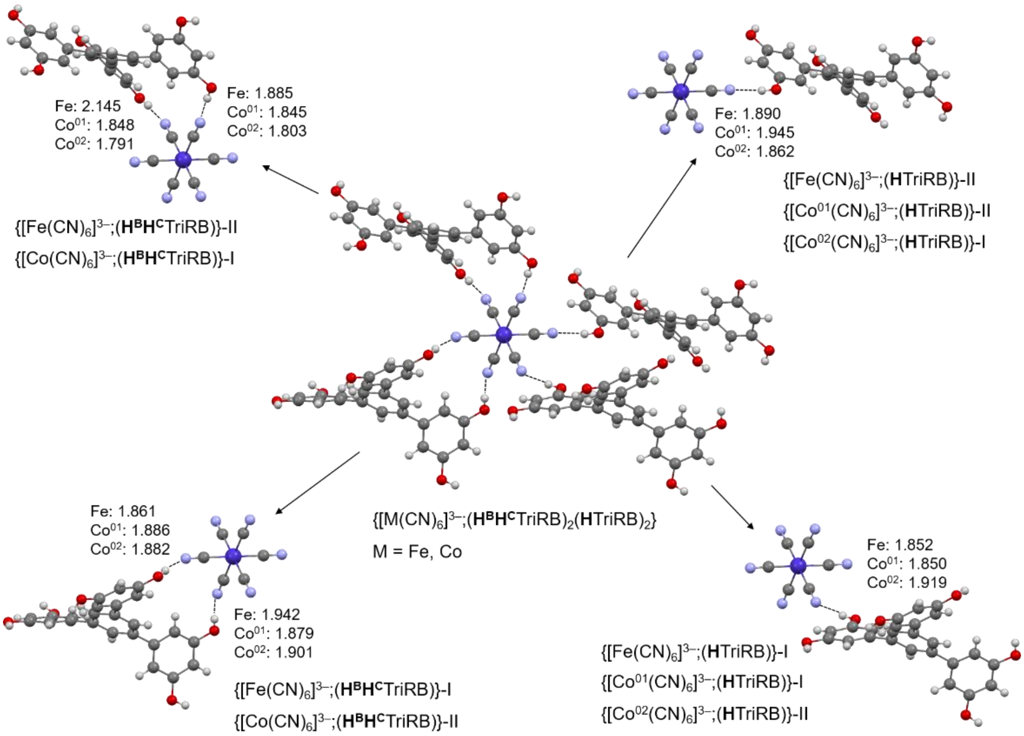

Figure S12. Visualization of the examined molecular clusters $\{[M(CN)_6]^{3-}$;(**H**$^{\mathbf{B}}$**H**$^{\mathbf{C}}$TriRB)$_2$(**H**TriRB)$_2\}$, $\{[M(CN)_6]^{3-}$;(**H**$^{\mathbf{B}}$**H**$^{\mathbf{C}}$TriRB)$\}$, and $\{[M(CN)_6]^{3-}$;(**H**TriRB)$\}$ extracted from the crystal structures of **MTriRB** with M = Fe and Co. Note that for **CoTriRB** there are two different $[Co(CN)_6]^{3-}$ anions in the asymmetric unit (Co^{01} and Co^{02}), hence two sets of motifs were examined. Numbers listed are O–H···N distances, in Å.

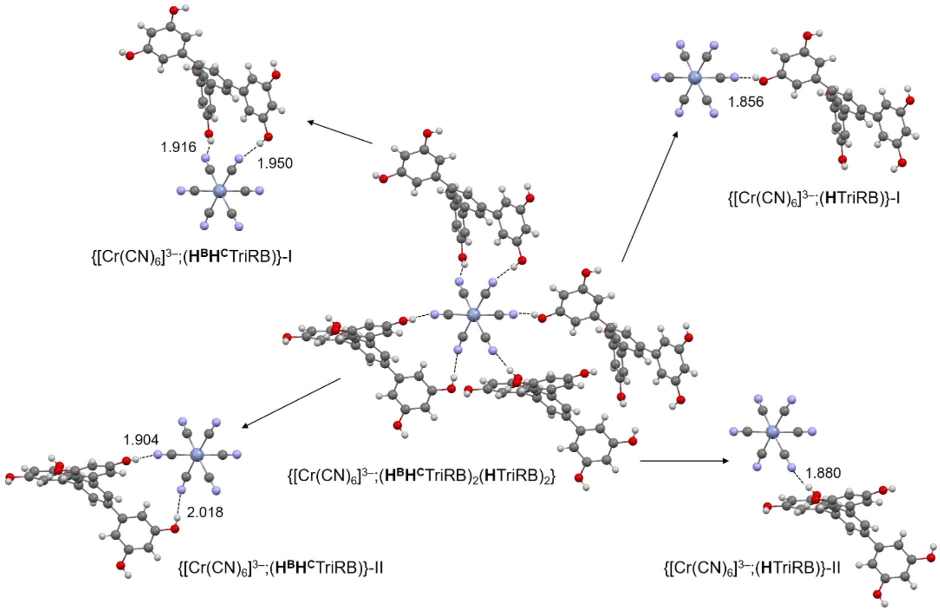

Figure S13. Visualization of the examined molecular clusters $\{[Cr(CN)_6]^{3-}$;(**H**$^{\mathbf{B}}$**H**$^{\mathbf{C}}$TriRB)$_2$(**H**TriRB)$_2\}$, $\{[Cr(CN)_6]^{3-}$;(**H**$^{\mathbf{B}}$**H**$^{\mathbf{C}}$TriRB)$\}$, and $\{[Cr(CN)_6]^{3-}$;(**H**TriRB)$\}$ extracted from the crystal structure of **CrTriRB**. Numbers listed are O–H···N distances, in Å.

2.2. Calculated data

As expected and directly shown by the results of extended transition state – natural orbitals for chemical valence (ETS-NOCV) charge and bonding-energy decomposition analyses performed for the closed-shell motifs ($[Co(CN)_6]^{3-}$/DiR and $[Co(CN)_6]^{3-}$/TriRB: **Tables S12** and **S14**, **Figures S14-S17** and **S19-S20**), the $[M(CN)_6]^{3-}$/DiR and $[M(CN)_6]^{3-}$/TriRB interactions in **MDiR** and **MTriRB** involve hydrogen bonding interaction. Its covalent nature is clearly visible in the dominant NOCV contributions to the differential electron density (redistribution of electron density around $[Co(CN)_6]^{3-}$ and DiR or TriRB in the considered molecular clusters) *via* hydrogen bond acceptor and donor charge-transfer (CT) interaction between the occupied lone pair of nitrogen and the unoccupied σ* orbital of the O–H bond (σ-CT) with participation of π-type orbitals of both moieties. Energetically relevant NOCV contributions demonstrate also the strong polarization (intra-CT) of the π-electron system within the hydrogen bond donor DiR or TriRB molecule, facilitated by the ion-dipole interaction imposed by the negative charge of the hydrogen bond acceptor $[M(CN)_6]^{3-}$. These two effects underly significantly negative (stabilizing) values of orbital-interaction ΔE_{orb} contribution to the total interaction energies, which is accompanied by overall even more stabilizing electrostatic ΔE_{elstat} term, lead to the strong interactions between $[M(CN)_6]^{3-}$ and DiR or TriRB, similar to what we previously reported for the $[M(CN)_6]^{3-}$/phloroglucinol system in Reference S2. This also accounts for a pronounced decrease in the magnitude of the total interaction energy observed for the {(DiR);(**H^A^**DiR)} motif vs. the corresponding {$[M(CN)_6]^{3-}$;(**H^A^**DiR)} one (**Tables S11-S12**, **Figures S17-S18**), although in part this drop is probably due to not optimal arrangement of the DiR molecules enforced by the overall crystal structure network.

Table S11. DFT-computed (B3LYP+D4//TZP, BLYP+D4//TZP in parentheses) interaction energy values (in kcal/mol) between hexacyanometallate anion $[M(CN)_6]^{3-}$ (M = Cr, Fe, and Co) and polyresorcinol DiR molecule(s) in molecular clusters $\{[M(CN)_6]^{3-};(\mathbf{H^AH^B}\mathrm{DiR})_2(\mathbf{H^A}_2\mathrm{DiR})(\mathbf{H^A}\mathrm{DiR})\}$, $\{[M(CN)_6]^{3-};(\mathbf{H^AH^B}\mathrm{DiR})\}$, $\{[M(CN)_6]^{3-};(\mathbf{H^A}_2\mathrm{DiR})(\mathbf{H^A}\mathrm{DiR})\}$, $\{[M(CN)_6]^{3-};(\mathbf{H^A}_2\mathrm{DiR})\}$, and $\{[M(CN)_6]^{3-};(\mathbf{H^A}\mathrm{DiR})\}$, and between the DiR molecules in $\{(\mathrm{DiR});(\mathbf{H^A}\mathrm{DiR})\}$ motif, extracted from the crystal structures of **MDiR** with M = Cr, Fe, and Co. For clusters visualization, see **Figure S11**. 2HB/1HB stands for double/single hydrogen bonding interaction.

	$[Cr(CN)_6]^{3-}$	$[Fe(CN)_6]^{3-}$	$[Co(CN)_6]^{3-}$	DiR
$(\mathbf{H^AH^B}\mathrm{DiR})_2(\mathbf{H^A}_2\mathrm{DiR})(\mathbf{H^A}\mathrm{DiR})$	-163.34<br>(-164.24)	-168.43<br>(-169.51)	-171.23<br>(-172.16)	–
$(\mathbf{H^AH^B}\mathrm{DiR})$-I<br>*side 2HB*	-51.07<br>(-51.62)	-53.64<br>(-54.70)	-54.50<br>(-55.02)	–
$(\mathbf{H^AH^B}\mathrm{DiR})$-II<br>*side 2HB*	-50.23<br>(-50.87)	-50.77<br>(-52.04)	-52.13<br>(-52.79)	–
$(\mathbf{H^A}_2\mathrm{DiR})(\mathbf{H^A}\mathrm{DiR})$<br>*frontal 2HB & 1HB*	-78.80<br>(-80.74)	-82.03<br>(#)	-82.70<br>(-84.32)	–
$\mathbf{H^A}_2\mathrm{DiR}$<br>*frontal 2HB*	-45.97<br>(-48.29)	-47.94<br>(#)	-49.06<br>(†)	–
$\mathbf{H^A}\mathrm{DiR}$<br>*frontal 1HB*	-36.05<br>(-40.78)	-37.48<br>(#)	-37.24<br>(†)	Cr: -5.60 (-5.50)<br>Fe: -5.46 (-5.35)<br>Co: -5.24 (-5.14)

† Calculations failed to reach SCF convergence for the motif. # Obtained results were considered unreliable due to significant spin-contamination in the wavefunction of the molecular cluster (expectation value of S^2 ~ 0.93-1.10 vs. exact 0.75).

Table S12. Hydrogen bond acceptor ($[\mathrm{Co(CN)_6}]^{3-}$ or DiR) / hydrogen bond donor (DiR) energy interaction values and their components (in kcal/mol) according to the ETS energy decomposition scheme for the molecular clusters $\{[\mathrm{Co(CN)_6}]^{3-};(\mathbf{H^AH^B}\mathrm{DiR})\}$, $\{[\mathrm{Co(CN)_6}]^{3-};(\mathbf{H^A}_2\mathrm{DiR})(\mathbf{H^A}\mathrm{DiR})\}$, $\{[\mathrm{Co(CN)_6}]^{3-};(\mathbf{H^A}_2\mathrm{DiR})\}$, $\{[\mathrm{Co(CN)_6}]^{3-};(\mathbf{H^A}\mathrm{DiR})\}$, and $\{(\mathrm{DiR});(\mathbf{H^A}\mathrm{DiR})\}$ extracted from the crystal structures of **MDiR** with M = Cr, Fe, and Co. For clusters visualization, see **Figure S11**. Based on B3LYP+D4//TZP and BLYP+D4//TZP (in parentheses) calculations. 2HB/1HB stands for double/single hydrogen bonding interaction.

Molecular cluster		ΔE_{int}	ΔE_{orb}	ΔE_{Pauli}	ΔE_{elstat}	ΔE_{disp}
$\{[\mathrm{Co(CN)_6}]^{3-};(\mathbf{H^AH^B}\mathrm{DiR})\}$-I<br>*side 2HB*		-54.50<br>(-55.02)	-30.49<br>(-32.23)	26.78<br>(29.40)	-45.76<br>(-45.94)	-5.03<br>(-6.26)
$\{[\mathrm{Co(CN)_6}]^{3-};(\mathbf{H^AH^B}\mathrm{DiR})\}$-II<br>*side 2HB*		-52.13<br>(-52.79)	-29.28<br>(-31.02)	24.53<br>(27.02)	-42.30<br>(-42.50)	-5.07<br>(-6.28)
$\{[\mathrm{Co(CN)_6}]^{3-};(\mathbf{H^A}_2\mathrm{DiR})(\mathbf{H^A}\mathrm{DiR})\}$<br>*frontal 2HB & 1HB*		-82.70<br>(-84.32)	-46.14<br>(-49.23)	36.29<br>(40.02)	-64.25<br>(-64.50)	-8.59<br>(-10.62)
$\{[\mathrm{Co(CN)_6}]^{3-};(\mathbf{H^A}_2\mathrm{DiR})\}$<br>*frontal 2HB*		-49.06<br>(†)	-28.32<br>(†)	22.51<br>(†)	-37.07<br>(†)	-6.19<br>(†)
$\{[\mathrm{Co(CN)_6}]^{3-};(\mathbf{H^A}\mathrm{DiR})\}$<br>*frontal 1HB*		-37.24<br>(†)	-19.87<br>(†)	13.78<br>(†)	-28.36<br>(†)	-2.80<br>(†)
$\{(\mathrm{DiR});(\mathbf{H^A}\mathrm{DiR})\}$<br>*frontal 1HB*	Cr:	-5.60<br>(-5.50)	-3.18<br>(-3.47)	6.75<br>(7.73)	-6.23<br>(-6.27)	-2.94<br>(-3.48)
	Fe:	-5.46<br>(-5.35)	-3.67<br>(-4.00)	8.21<br>(9.26)	-6.88<br>(-6.93)	-3.11<br>(-3.69)
	Co:	-5.24<br>(-5.14)	-2.95<br>(-3.22)	6.60<br>(7.57)	-5.90<br>(-5.95)	-2.99<br>(-3.53)

† Calculations failed to reach SCF convergence for the motif.

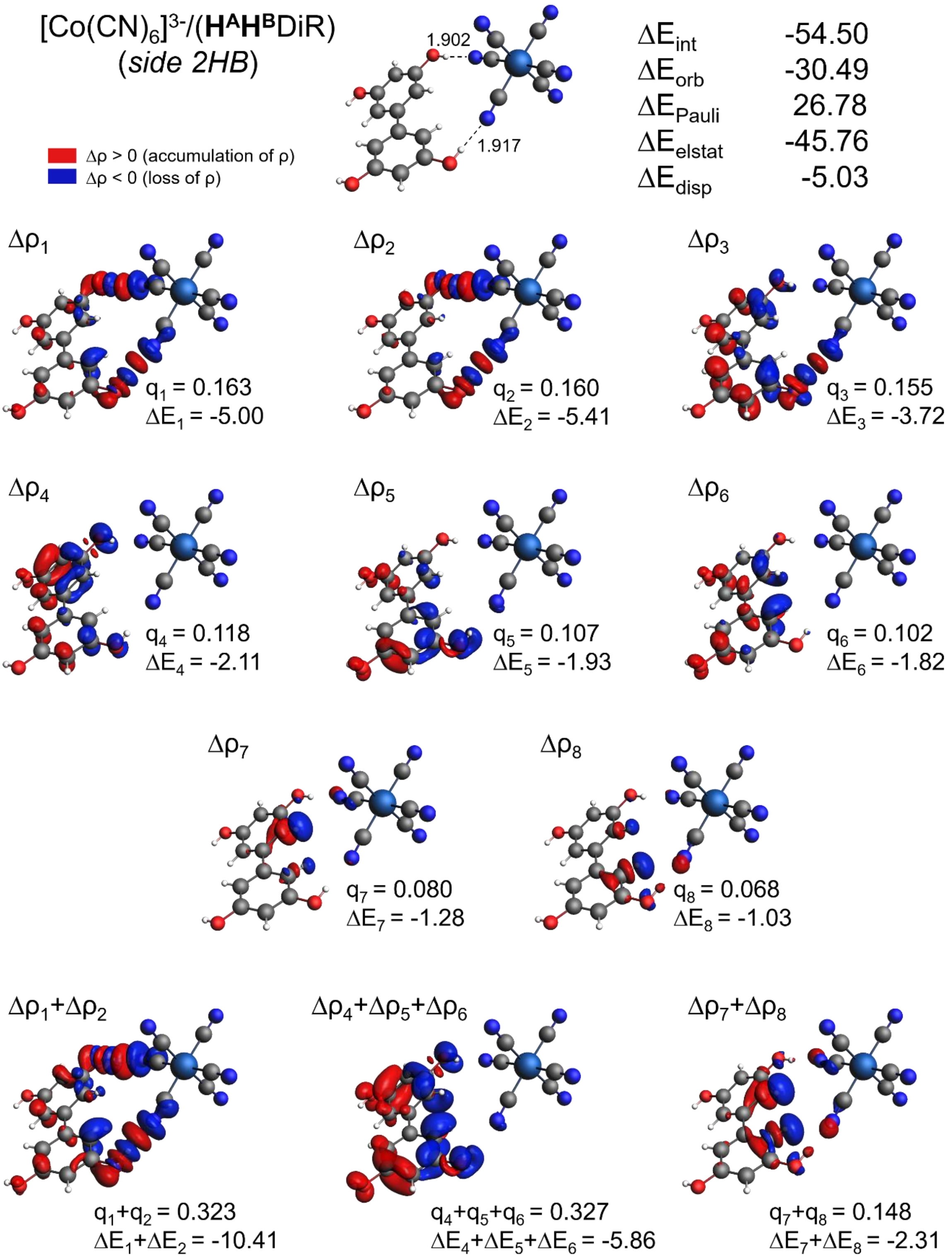

Figure S14. ETS-NOCV analysis of the interaction between $[Co(CN)_6]^{3-}$ and DiR molecule in the molecular cluster $\{[Co(CN)_6]^{3-}$;(**H**$^{\mathbf{A}}$**H**$^{\mathbf{B}}$DiR)}-I extracted from the crystal structure of **CoDiR**. Top: Molecular cluster $\{[Co(CN)_6]^{3-}$;(**H**$^{\mathbf{A}}$**H**$^{\mathbf{B}}$DiR)}-I with the hydrogen bond distances (in Å) listed. $[Co(CN)_6]^{3-}$/(**H**$^{\mathbf{A}}$**H**$^{\mathbf{B}}$DiR) energy interaction value and its components (in kcal/mol) according to the ETS energy decomposition scheme. Bottom: Isosurfaces (±0.0005 au) of dominant NOCV contributions to charge deformation (differential) density Δρ along with their corresponding charge (q in e) and orbital energy (ΔE in kcal/mol) assessments. Red/blue indicates inflow (gain)/outflow (loss) of electron density. Based on B3LYP+D4//TZP calculations.

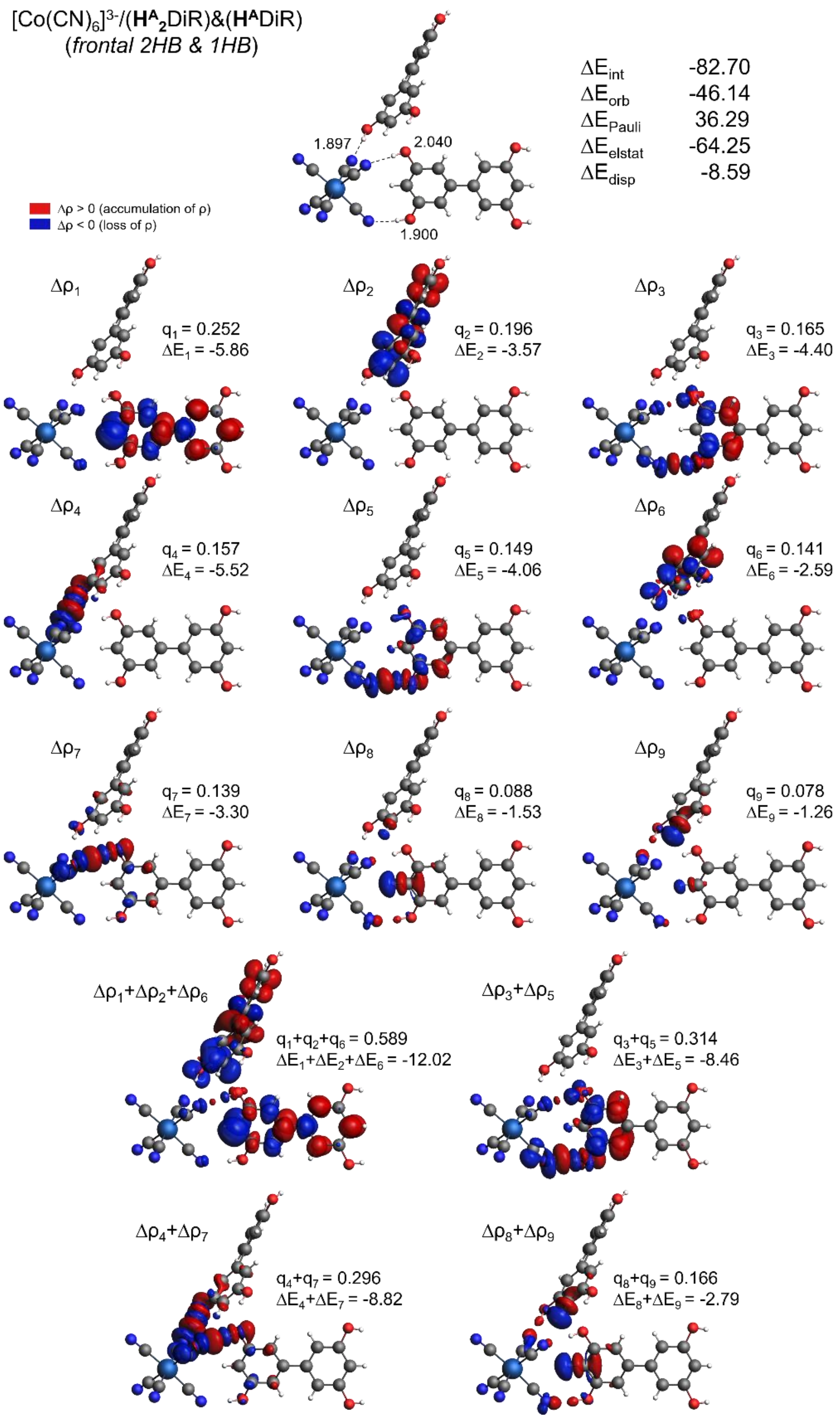

Figure S15. ETS-NOCV analysis of the interaction between $[Co(CN)_6]^{3-}$ and DiR molecules in the molecular cluster $\{[Co(CN)_6]^{3-}$;(**H$^A_2$**DiR)(**H^A**DiR)} extracted from the crystal structure of **CoDiR**. Top: Molecular cluster $\{[Co(CN)_6]^{3-}$;(**H$^A_2$**DiR)(**H^A**DiR)} with the hydrogen bond distances (in Å) listed. $[Co(CN)_6]^{3-}$/(**H$^A_2$**DiR)&(**H^A**DiR) energy interaction value and its components (in kcal/mol) according to the ETS energy decomposition scheme. Bottom: Isosurfaces (±0.0005 au) of dominant NOCV contributions to charge deformation (differential) density Δρ along with their corresponding charge (q in e) and orbital energy (ΔE in kcal/mol) assessments. Red/blue indicates inflow (gain)/outflow (loss) of electron density. Based on B3LYP+D4//TZP calculations.

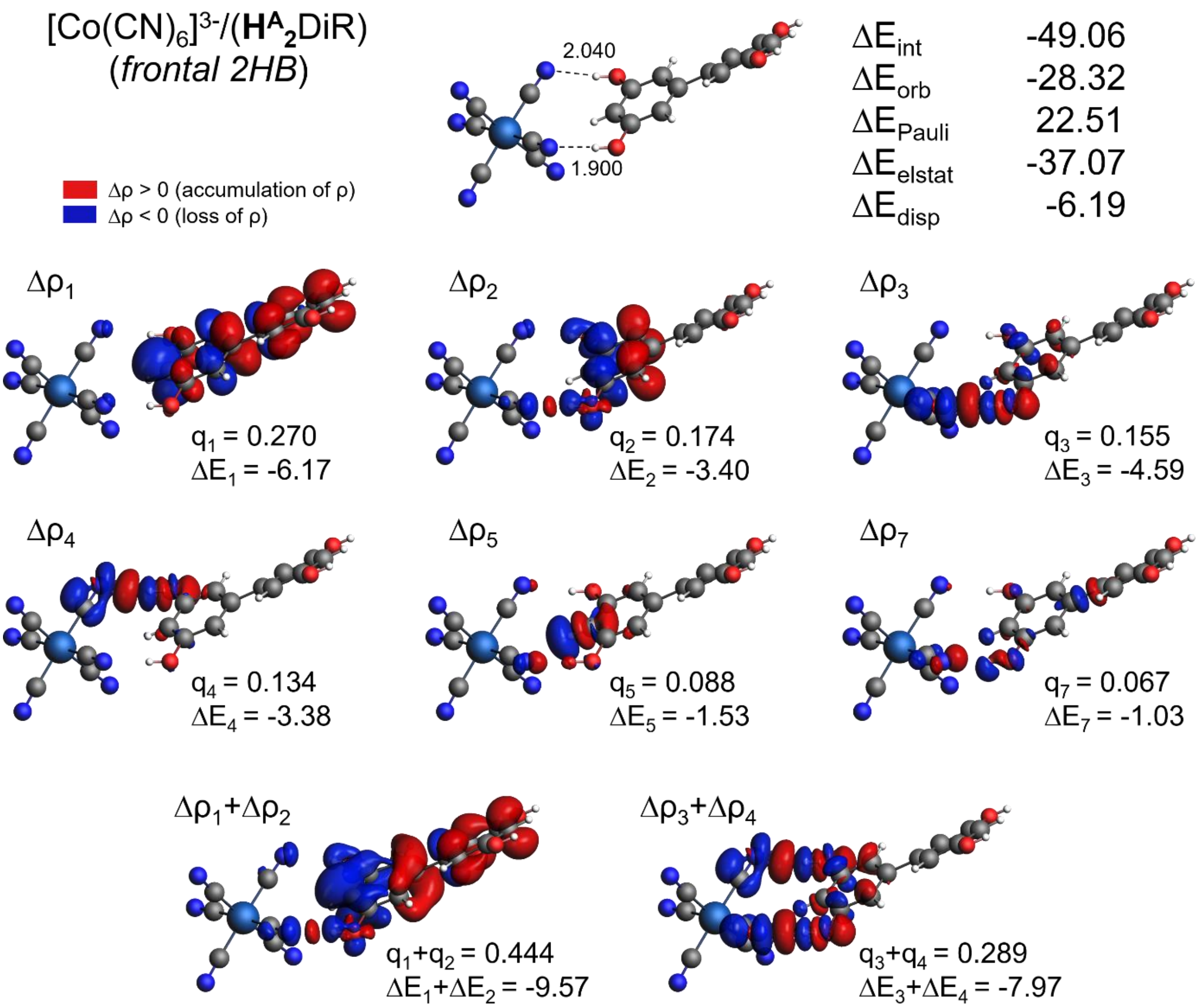

Figure S16. ETS-NOCV analysis of the interaction between $[Co(CN)_6]^{3-}$ and DiR molecule in the molecular cluster $\{[Co(CN)_6]^{3-};(\mathbf{H^A{}_2}DiR)\}$ extracted from the crystal structure of **CoDiR**. Top: Molecular cluster $\{[Co(CN)_6]^{3-};(\mathbf{H^A{}_2}DiR)\}$ with the hydrogen bond distances (in Å) listed. $[Co(CN)_6]^{3-}/(\mathbf{H^A{}_2}DiR)$ energy interaction value and its components (in kcal/mol) according to the ETS energy decomposition scheme. Bottom: Isosurfaces (±0.0005 au) of dominant NOCV contributions to charge deformation (differential) density Δρ along with their corresponding charge (q in e) and orbital energy (ΔE in kcal/mol) assessments. Red/blue indicates inflow (gain)/outflow (loss) of electron density. Based on B3LYP+D4//TZP calculations.

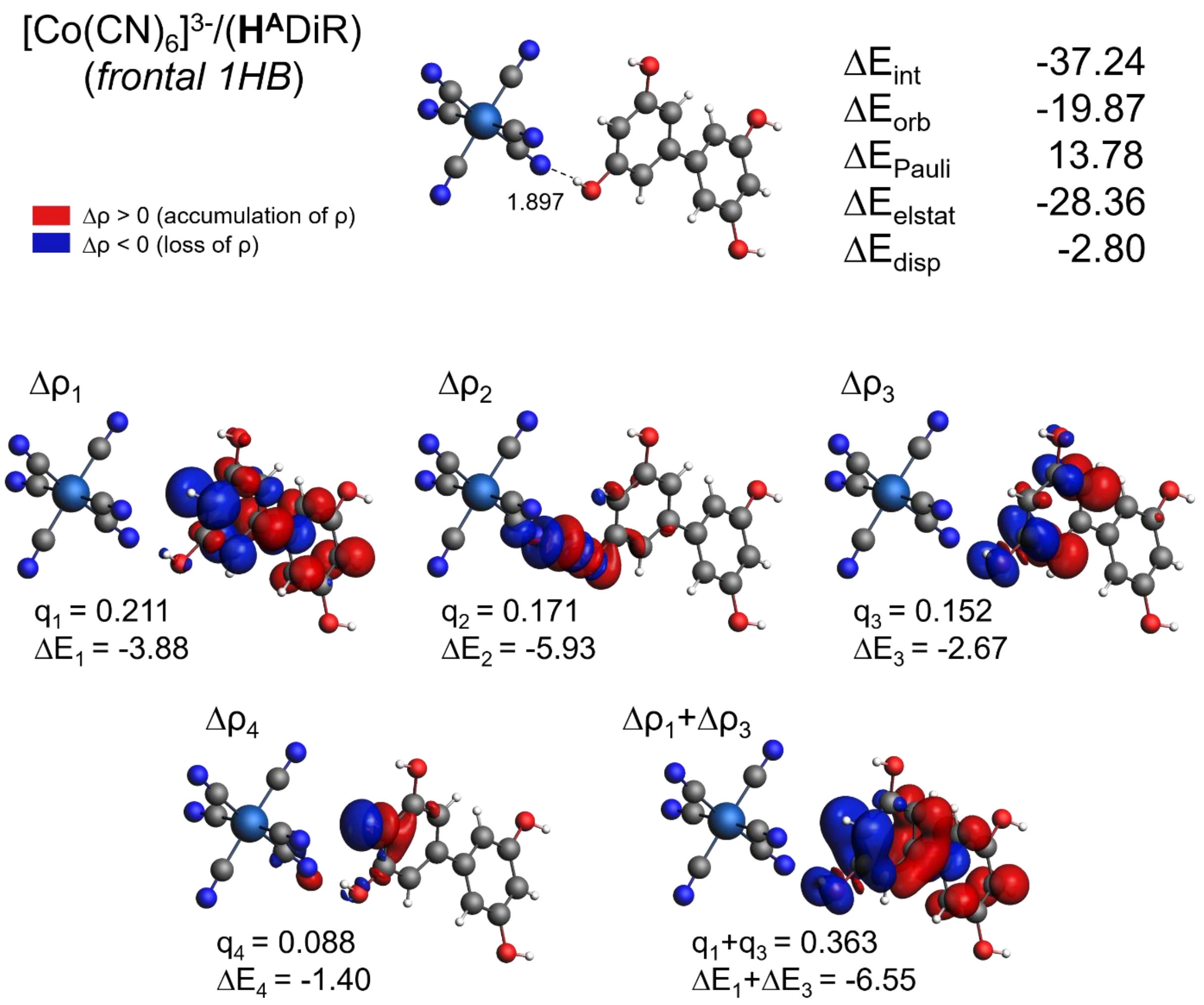

Figure S17. ETS-NOCV analysis of the interaction between $[Co(CN)_6]^{3-}$ and DiR molecule in the molecular cluster $\{[Co(CN)_6]^{3-}$;(**H**$^{\mathbf{A}}$DiR)} extracted from the crystal structure of **CoDiR**. Top: Molecular cluster $\{[Co(CN)_6]^{3-}$;(**H**$^{\mathbf{A}}$DiR)} with the hydrogen bond distance (in Å) listed. $[Co(CN)_6]^{3-}$/(**H**$^{\mathbf{A}}$DiR) energy interaction value and its components (in kcal/mol) according to the ETS energy decomposition scheme. Bottom: Isosurfaces (±0.0005 au) of dominant NOCV contributions to charge deformation (differential) density Δρ along with their corresponding charge (q in e) and orbital energy (ΔE in kcal/mol) assessments. Red/blue indicates inflow (gain)/outflow (loss) of electron density. Based on B3LYP+D4//TZP calculations.

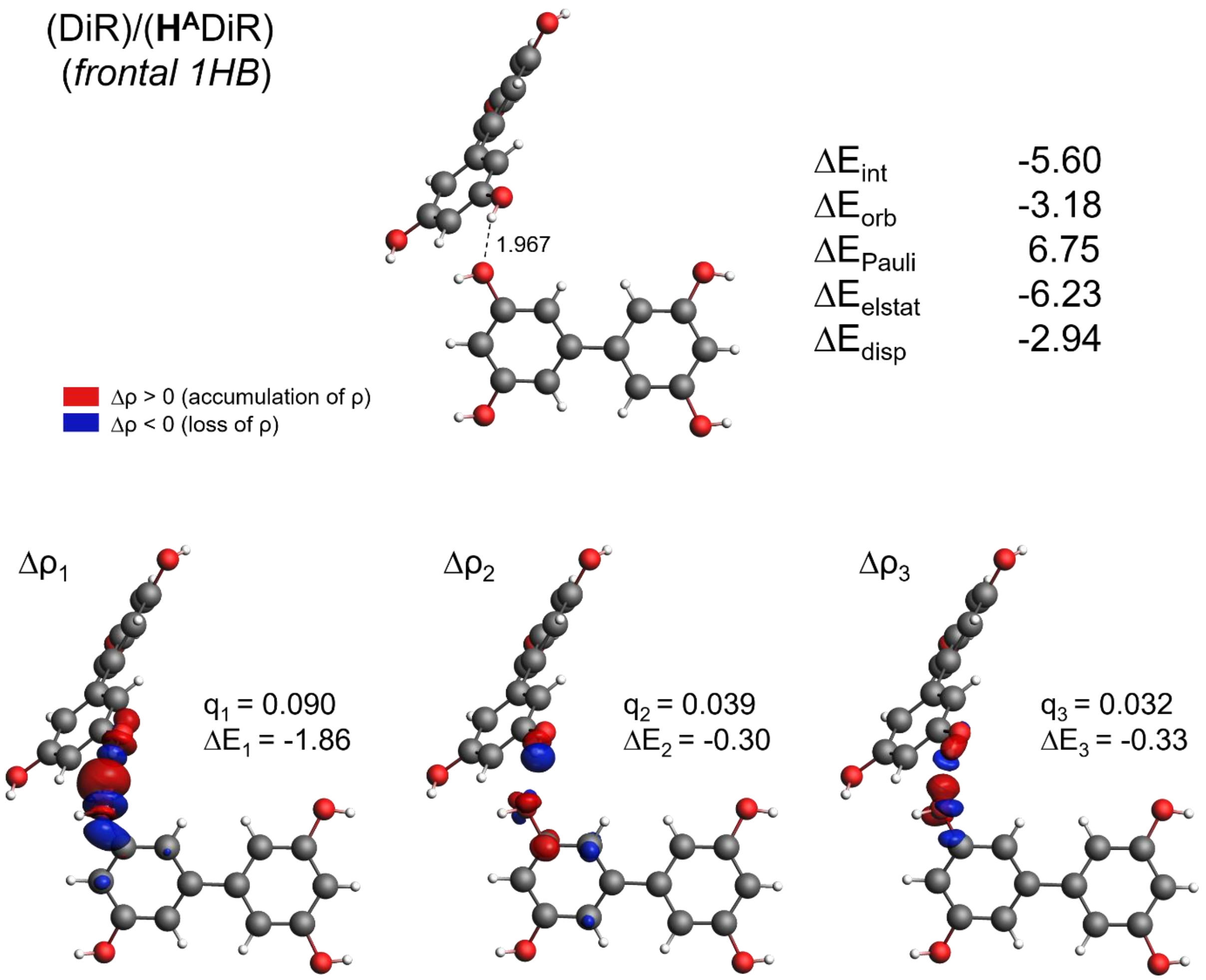

Figure S18. ETS-NOCV analysis of the interaction between two DiR molecules in the molecular cluster {(DiR);(**H**^{A}DiR)} extracted from the crystal structure of **CrDiR**. Top: Molecular cluster {(DiR);(**H**^{A}DiR)} with the hydrogen bond distance (in Å) listed. (DiR)/(**H**^{A}DiR) energy interaction value and its components (in kcal/mol) according to the ETS energy decomposition scheme. Bottom: Isosurfaces (±0.0005 au) of dominant NOCV contributions to charge deformation (differential) density Δρ along with their corresponding charge (q in e) and orbital energy (ΔE in kcal/mol) assessments. Red/blue indicates inflow (gain)/outflow (loss) of electron density. Based on B3LYP+D4//TZP calculations.

Table S13. DFT-computed (B3LYP+D4//TZP, BLYP+D4//TZP in parentheses, values obtained with B3LYP+D3//TZVP using Gaussian 16 indicated by the superscript G16) interaction energy values (in kcal/mol) between hexacyanometallate anion $[M(CN)_6]^{3-}$ (M = Cr, Fe, Co) and polyresorcinol TriRB molecule(s) in molecular clusters $\{[M(CN)_6]^{3-}$;(**H**^{B}**H**^{C}TriRB)$_2$(**H**TriRB)$_2\}$, $\{[M(CN)_6]^{3-}$;(**H**^{B}**H**^{C}TriRB)$\}$, and $\{[M(CN)_6]^{3-}$;(**H**TriRB)$\}$ extracted from the crystal structures of **MTriRB** with M = Cr, Fe, and Co. For clusters visualization, see **Figures S12** and **S13**. 2HB/1HB stands for double/single hydrogen bonding interaction.

	$[Cr(CN)_6]^{3-}$	$[Fe(CN)_6]^{3-}$	$[Co^{01}(CN)_6]^{3-}$	$[Co^{02}(CN)_6]^{3-}$
(**H**^{B}**H**^{C}TriRB)$_2$(**H**TriRB)$_2$	-148.82<br>(-150.86)	-156.65<br>(#)	-158.27<br>(†)	-157.17<br>(-158.73)
(**H**^{B}**H**^{C}TriRB)-I<br>*side 2HB*	-54.54<br>(-57.39)	-58.86<br>(#)	-60.00<br>(†)	-58.63<br>(†)
(**H**^{B}**H**^{C}TriRB)-II<br>*side 2HB*	-54.90<br>(-57.79)	-55.72<br>(#)	-57.14<br>(†)	-57.84<br>(†)
(**H**TriRB)-I<br>*frontal 1HB*	-27.79 / -27.57^{G16}<br>(-40.45)	# / -30.36^{G16}<br>(#)	-29.87 / -30.01^{G16}<br>(†)	-31.32 / -31.39^{G16}<br>(†)
(**H**TriRB)-II<br>*frontal 1HB*	-29.46 / -29.30^{G16}<br>(-41.87)	# / #^{G16}<br>(#)	-30.42 / -30.53^{G16}<br>(†)	-28.92 / -29.14^{G16}<br>(†)

† Calculations failed to reach SCF convergence for the motif. # Obtained results were considered unreliable due to significant spin-contamination in the wavefunction of the molecular cluster (expectation value of S^2 ~ 0.95-1.29 vs. exact 0.75).

Table S14. Hydrogen bond acceptor ($[Co(CN)_6]^{3-}$) / hydrogen bond donor (TriRB) energy interaction values and their components (in kcal/mol) according to the ETS energy decomposition scheme for the molecular clusters {$[Co(CN)_6]^{3-}$;(**H**$^{\mathbf{B}}$**H**$^{\mathbf{C}}$TriRB)} and {$[Co(CN)_6]^{3-}$;(**H**TriRB)} extracted from the crystal structure of **CoTriRB**. For clusters visualization, see **Figure S12**. Based on B3LYP+D4//TZP calculations. 2HB/1HB stands for double/single hydrogen bonding interaction.

Molecular cluster	ΔE_{int}	ΔE_{orb}	ΔE_{Pauli}	ΔE_{elstat}	ΔE_{disp}
{$[Co^{01}(CN)_6]^{3-}$;(**H**$^{\mathbf{B}}$**H**$^{\mathbf{C}}$TriRB)}-I *side 2HB*	-60.00	-38.59	33.09	-48.88	-5.62
{$[Co^{01}(CN)_6]^{3-}$;(**H**$^{\mathbf{B}}$**H**$^{\mathbf{C}}$TriRB)}-II *side 2HB*	-57.14	-35.61	28.08	-44.56	-5.06
{$[Co^{01}(CN)_6]^{3-}$;(**H**TriRB)}-I *frontal 1HB*	-29.87	-21.63	15.87	-21.20	-2.92
{$[Co^{01}(CN)_6]^{3-}$;(**H**TriRB)}-II *frontal 1HB*	-30.42	-19.81	12.14	-20.04	-2.71
{$[Co^{02}(CN)_6]^{3-}$;(**H**$^{\mathbf{B}}$**H**$^{\mathbf{C}}$TriRB)}-I *side 2HB*	-58.63	-40.67	39.06	-51.19	-5.84
{$[Co^{02}(CN)_6]^{3-}$;(**H**$^{\mathbf{B}}$**H**$^{\mathbf{C}}$TriRB)}-II *side 2HB*	-57.84	-36.06	28.27	-44.89	-5.15
{$[Co^{02}(CN)_6]^{3-}$;(**H**TriRB)}-I *frontal 1HB*	-31.32	-21.60	15.61	-22.55	-2.78
{$[Co^{02}(CN)_6]^{3-}$;(**H**TriRB)}-II *frontal 1HB*	-28.92	-19.91	13.25	-19.37	-2.88

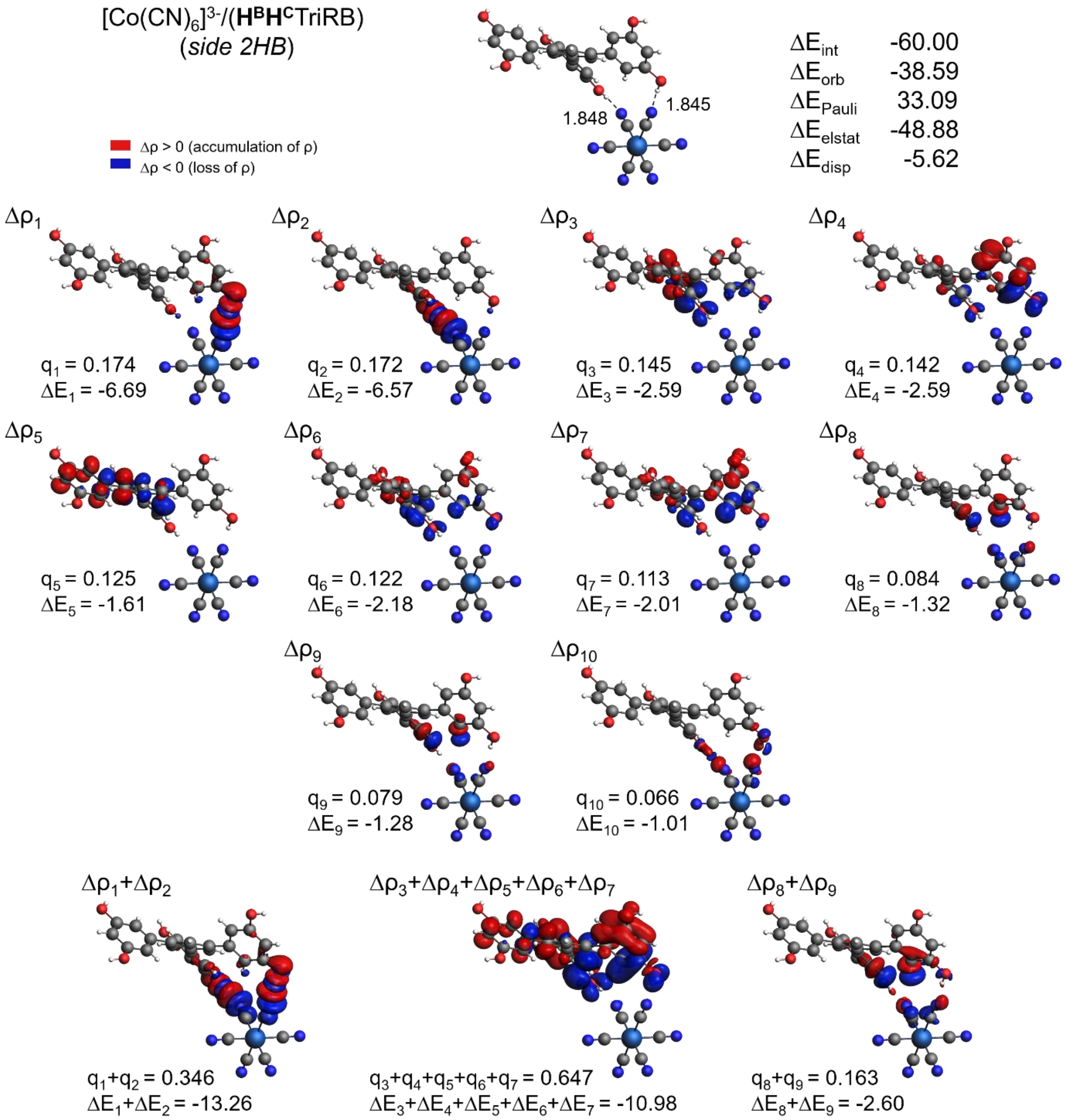

Figure S19. ETS-NOCV analysis of the interaction between $[Co(CN)_6]^{3-}$ and TriRB molecule in the molecular cluster $\{[Co^{01}(CN)_6]^{3-};(\mathbf{H^BH^C}TriRB)\}$-I extracted from the crystal structure of **CoTriRB**. Top: Molecular cluster $\{[Co^{01}(CN)_6]^{3-};(\mathbf{H^BH^C}TriRB)\}$-I with the hydrogen bond distances (in Å) listed. $[Co(CN)_6]^{3-}/(\mathbf{H^BH^C}TriRB)$ energy interaction value and its components (in kcal/mol) according to the ETS energy decomposition scheme. Bottom: Isosurfaces (±0.0005 au) of dominant NOCV contributions to charge deformation (differential) density Δρ along with their corresponding charge (q in e) and orbital energy (ΔE in kcal/mol) assessments. Red/blue indicates inflow (gain)/outflow (loss) of electron density. Based on B3LYP+D4//TZP calculations.

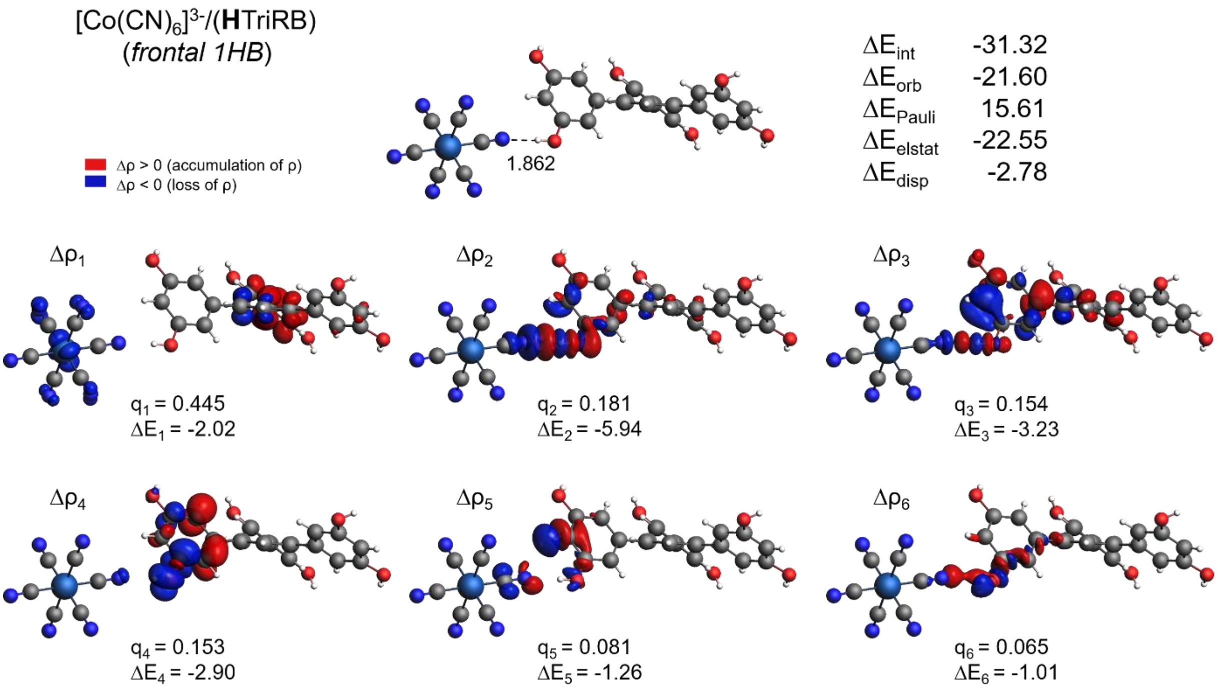

Figure S20. ETS-NOCV analysis of the interaction between $[Co(CN)_6]^{3-}$ and TriRB molecule in the molecular cluster $\{[Co^{02}(CN)_6]^{3-}$;(**H**TriRB)}-I extracted from the crystal structure of **CoTriRB**. Top: Molecular cluster $\{[Co^{02}(CN)_6]^{3-}$;(**H**TriRB)}-I with the hydrogen bond distance (in Å) listed. $[Co(CN)_6]^{3-}$/(**H**TriRB) energy interaction value and its components (in kcal/mol) according to the ETS energy decomposition scheme. Bottom: Isosurfaces (±0.0005 au) of dominant NOCV contributions to charge deformation (differential) density Δρ along with their corresponding charge (q in e) and orbital energy (ΔE in kcal/mol) assessments. Red/blue indicates inflow (gain)/outflow (loss) of electron density. Based on B3LYP+D4//TZP calculations.

3. Details of spectroscopic characterization

3.1. Vibrational studies

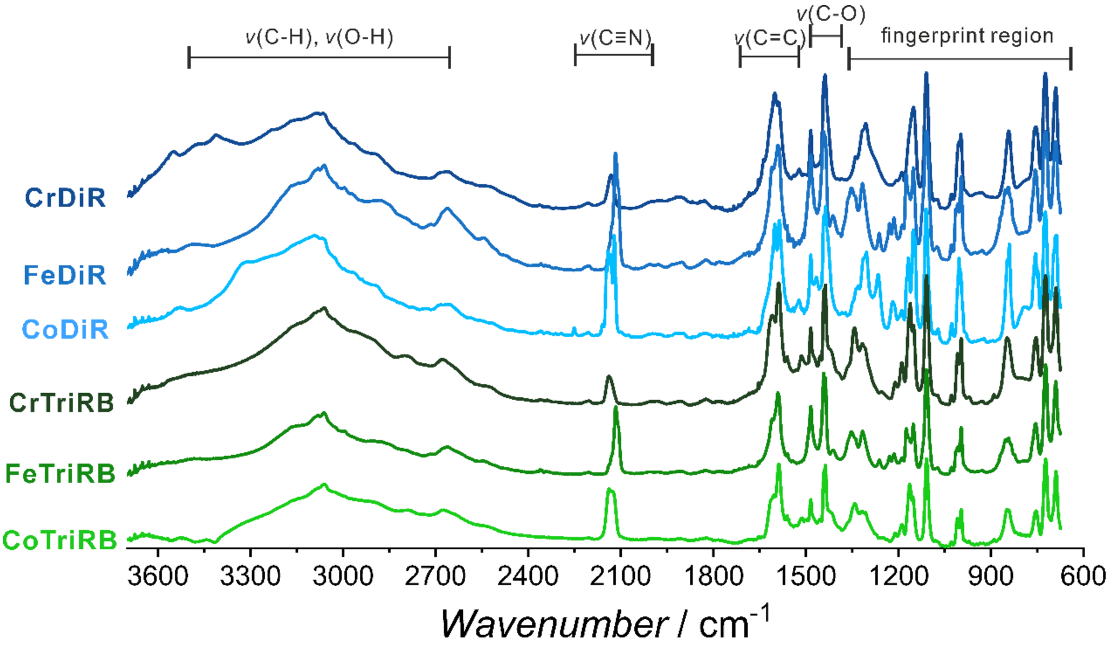

Figure S21. Room-temperature IR spectra for the single crystals of **CrDiR**, **FeDiR**, **CoDiR**, **CrTriRB**, **FeTriRB**, and **CoTriRB** measured in a wide range.

3.2. Room-temperature Raman scattering spectroscopy

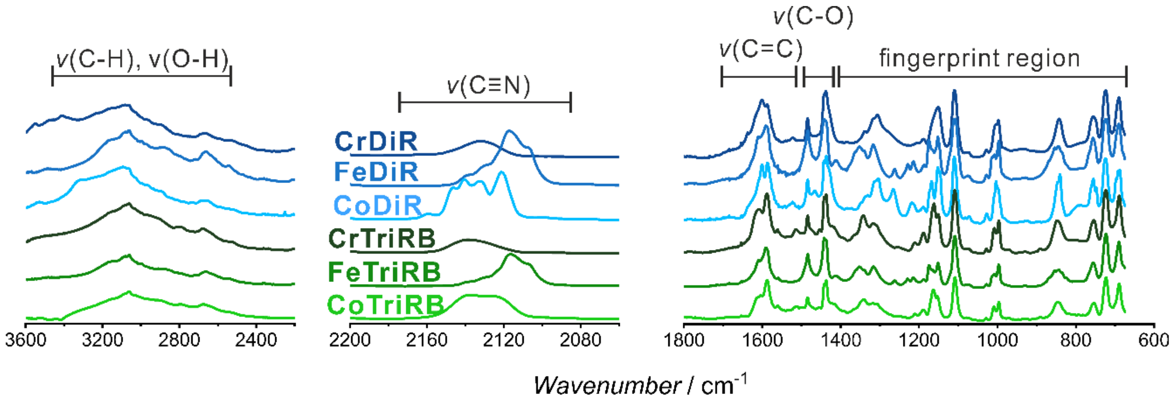

Figure S22. Infrared spectra of **MDiR** and **MTriRB** in the absorption mode, including the regions characteristic of the *ν*(O-H) and *ν*(C≡N) vibrations, and the skeletal vibrations in the fingerprint region.

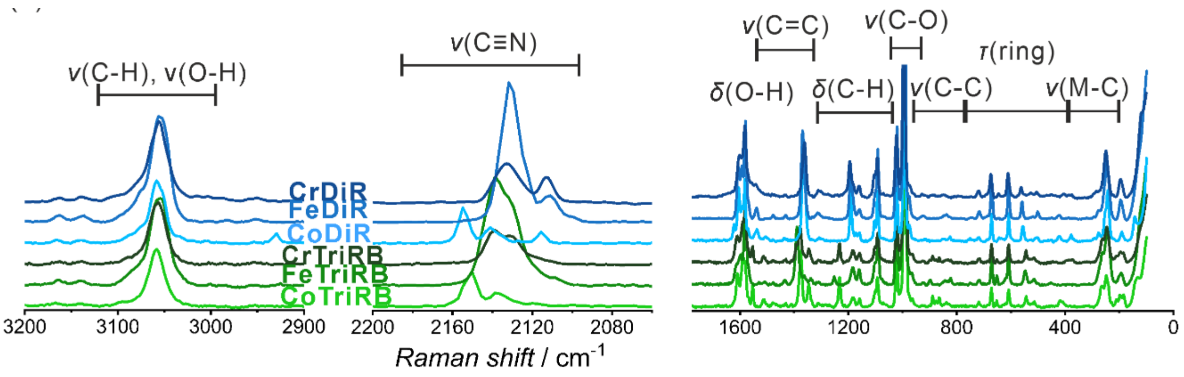

Figure S23. Room-temperature Raman scattering spectra for the single crystals of **MDiR** and **MTriRB** measured in a wide range.

IR and Raman spectra contain bands corresponding to molecular components and intermolecular synthons indicated by SC XRD analysis: *ν*(O-H) vibrations of DiR and TriRB; *ν*(C-H) vibrations of DiR, TriRB, PPh_4^+, MeOH, and MeCN; *ν*(C≡N) vibrations of $[M(CN)_6]^{3-}$ and MeCN, and a whole set of skeletal vibrations of DiR, TriRB, and PPh_4^+ (see Figures 21-23). The IR spectra of all the cocrystal salts exhibit a broad complex band within the 3600-2500 cm^{-1} range. This band is attributed to the vibrations of *ν*(O-H) within the extended charge-assisted hydrogen bond network as well as the vibrations of *ν*(C-H). The maximum intensity in this region is severely shifted towards *ca.* 3060 cm^{-1}, from *ca.* 3280 cm^{-1} observed in the spectra of both coformers in the native form. The following IR-active ν(C≡N) vibrations: 2132 cm^{-1} for **CrDiR**; 2138 cm^{-1} for **CrTriRB**; 2139, 2131, 2117, and 2107 cm^{-1} for **FeDiR**; 2139, 2131, 2116, and 2106 cm^{-1} for **FeTriRB**; 2159, 2146, 2132 and 2121 cm^{-1} for **CoDiR**; 2138 and 2124 cm^{-1} for **CoTriRB** are about 20 cm^{-1} shifted to a higher wavenumber compared to the respective precursors $(PPh_4)_3[M(CN)_6]$.[S2] The same effect is visible in the corresponding Raman spectra involving the following peak groups: 2133, 2123 and 2113 cm^{-1} for **CrDiR**; 2139, 2129 and 2121 cm^{-1} for **CrTriRB**; 2131, 2122, and 2112 cm^{-1} for **FeDiR**; 2139, 2129, and 2123 cm^{-1} for **FeTriRB**; 2154, 2140, and 2116 cm^{-1} for **CoDiR**; 2151, 2137, and 2119 cm^{-1} for **CoTriRB** (Figures S23). As for the skeletal coformer IR-active vibrations, the only extractable (e.g. free from the contributions of other components) essential spectral shifts of *ca.* 20 cm^{-1} (**DiR** line) or 30 cm^{-1} (**TriRB** line) compared to the solid specimens of coformers might be claimed in the region of 800-870 cm^{-1}, assignable to the composed *γ*(C-H) type vibrations.[S19] In the Raman spectra, the reasonably extractable spectral changes occur in the 1590-1640 cm^{-1} range of *ν*(C=C) and *δ*(O-H···D) vibrations and in the 1250-1190 cm^{-1} range of *β*(C-H) and *δ*(O-H···D) vibrations,[S20] D – hydrogen bond donor atom. All the observed modifications with respect to the reference solids are attributable to the specific hydrogen-bond networks involving $[M(CN)_6]^{3-}$ and coformers established in **MDiR** and **MTriRB** irrespective of the space group observed in both groups of compounds.

3.3. Low-frequency (LF) Raman scattering spectroscopy

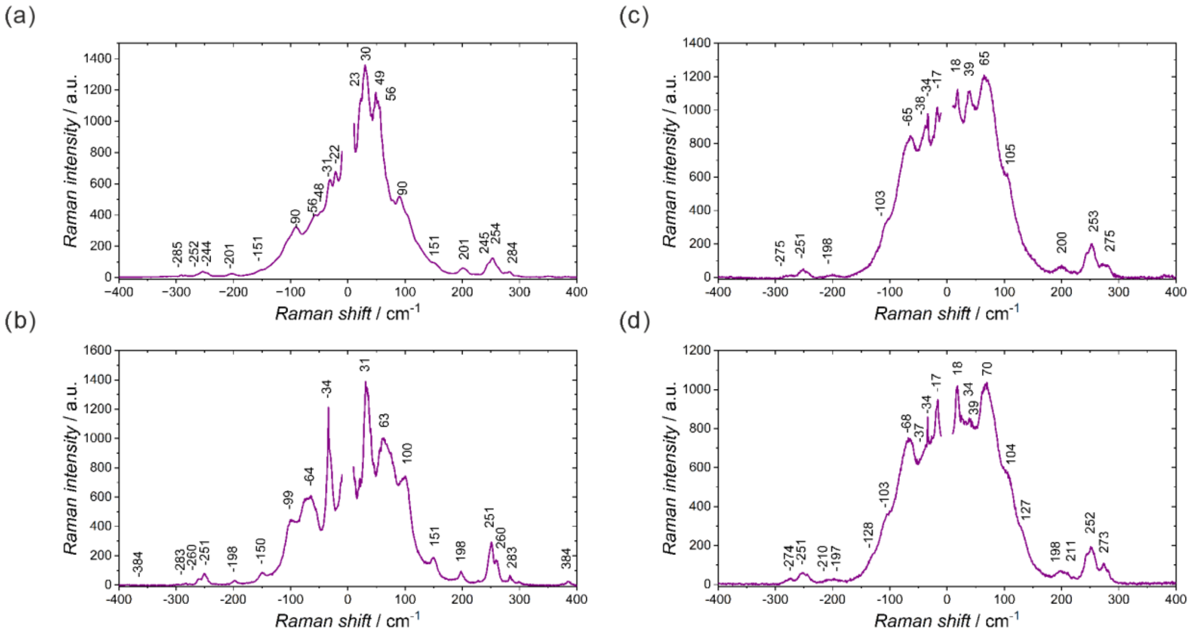

Figure S24. Room-temperature LF-Raman scattering spectra for the single crystals of **CrDiR** (a), CoDiR (b), **CrTriRB** (c), and **FeTriRB** (d). The numbers above the peaks correspond to the position of the peaks in cm^{-1}.

3.4. ^{57}Fe Mössbauer spectra

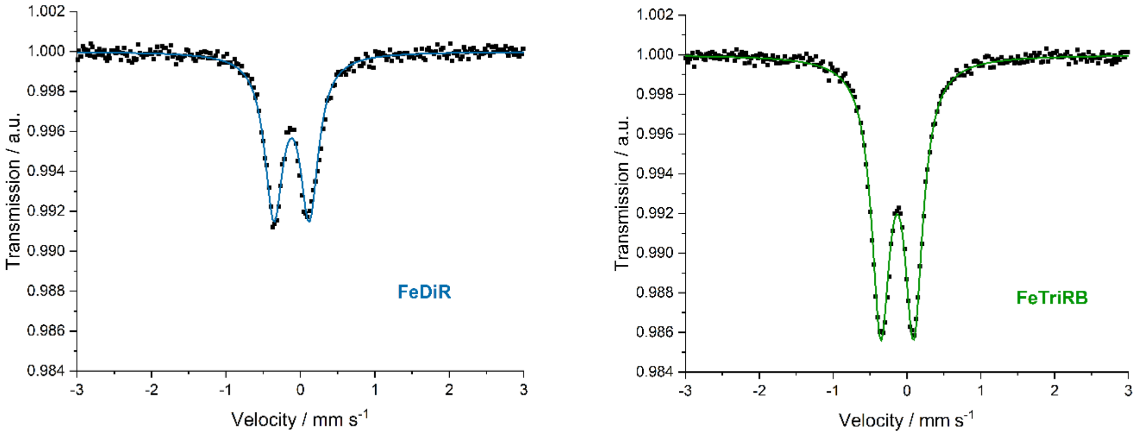

Figure S25. ^{57}Fe Mössbauer spectra of **FeDiR** (left) and of **FeTriRB** (right). The values of the fitted parameters δ = –0.12(1) mm s^{-1}, QS = 0.48(1) mm s^{-1} for **FeDiR**, and δ = –0.13(1) mm s^{-1}, QS = 0.45(1) mm s^{-1} for **FeTriRB**, are very close to the parameters sets obtained previously for **FeH$_3$PG**, –0.11 and 0.55 mm s^{-1}, in line with the presence of $_{L}$O-H···N$_{M\text{-}C\equiv N}$ hydrogen bonds.[S2]

3.5. UV-Vis electronic absorption spectra

UV-Vis spectra of cocrystal salts **MDiR** and **MTriRB** in the solid state, together with the spectra of the reference solids of the pristine coformers and metal complexes in the form of the Kubelka-Munk function, are presented in Figure S26. The spectra show diverse absorption threshold points depending on the $[M(CN)_6]^{3-}$ used, and remain in line with the results presented previously for the $\{M(CN)_6]^{3-}$;$H_3PG\}$ networks.[S2] The lowest-energy absorption bands for the Cr(III) containing compounds, centered around 379, 375, and 373 nm for **CrTriRB**, **CrDiR,** and $K_3[Cr(CN)_6]_3$ salt, respectively, correspond to the lowest-energy spin-allowed $^4T_{2g} \leftarrow {}^4A_{2g}$ transition for Cr^{3+} ions in the octahedral ligand field. The corresponding lowest-energy observed bands of $[Co(CN)_6]^{3-}$ moieties, spin-allowed $^1T_{1g} \leftarrow {}^1A_{1g}$ transitions with the maxima expected below 320 nm, are on the other hand hidden under the far more intense intra-ligand (IL) transitions and the CT transitions dominating in the UV-region. The Fe(III) based networks exhibit intense absorption bands with maxima at *ca.* 411 and 427 nm (**FeDiR**), *ca.* 410 and 430 nm (**FeTriRB**), and *ca.* 403 and 422 nm ($K_3[Fe(CN)_6]_3$), all assigned to the ligand-to-metal charge-transfer (LMCT) $\sigma(CN^-) \rightarrow \pi(t_{2g})$ transitions (Figure 5, Figure S26).

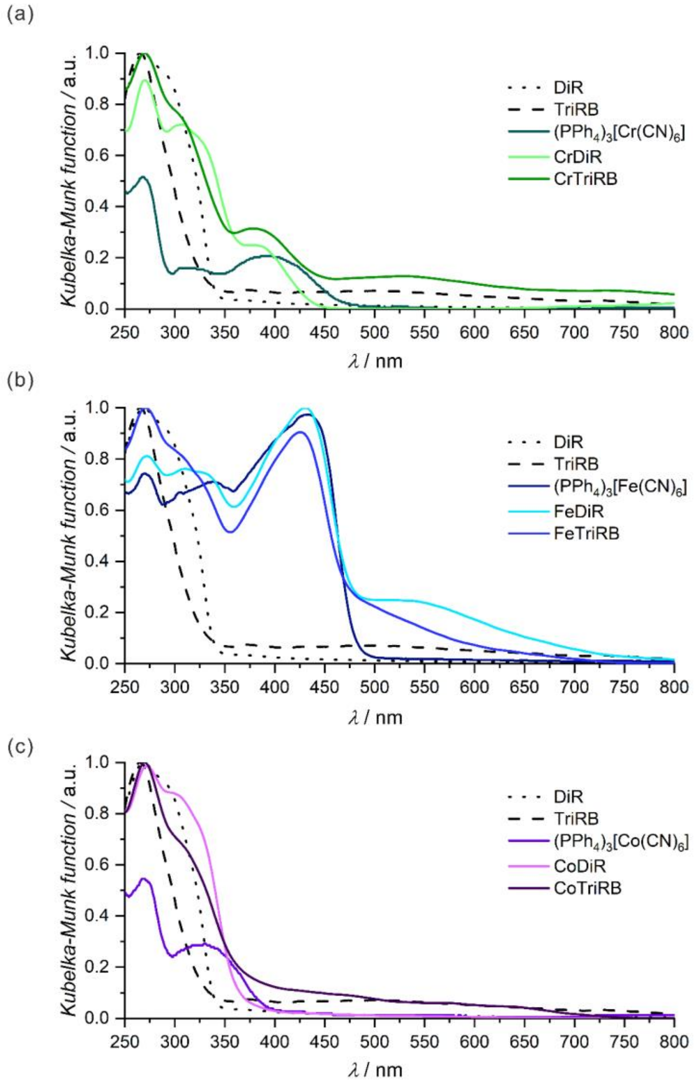

Figure S26. Solid-state UV-Vis absorption spectra of the **MDiR** and **MTriRB** compounds in the range of 250-800 nm together with those of DiR, TriRB and $PPh_4[M(CN)_6]\cdot nH_2O$ precursors as the references; M = Cr (a), Fe (b), and Co (c).

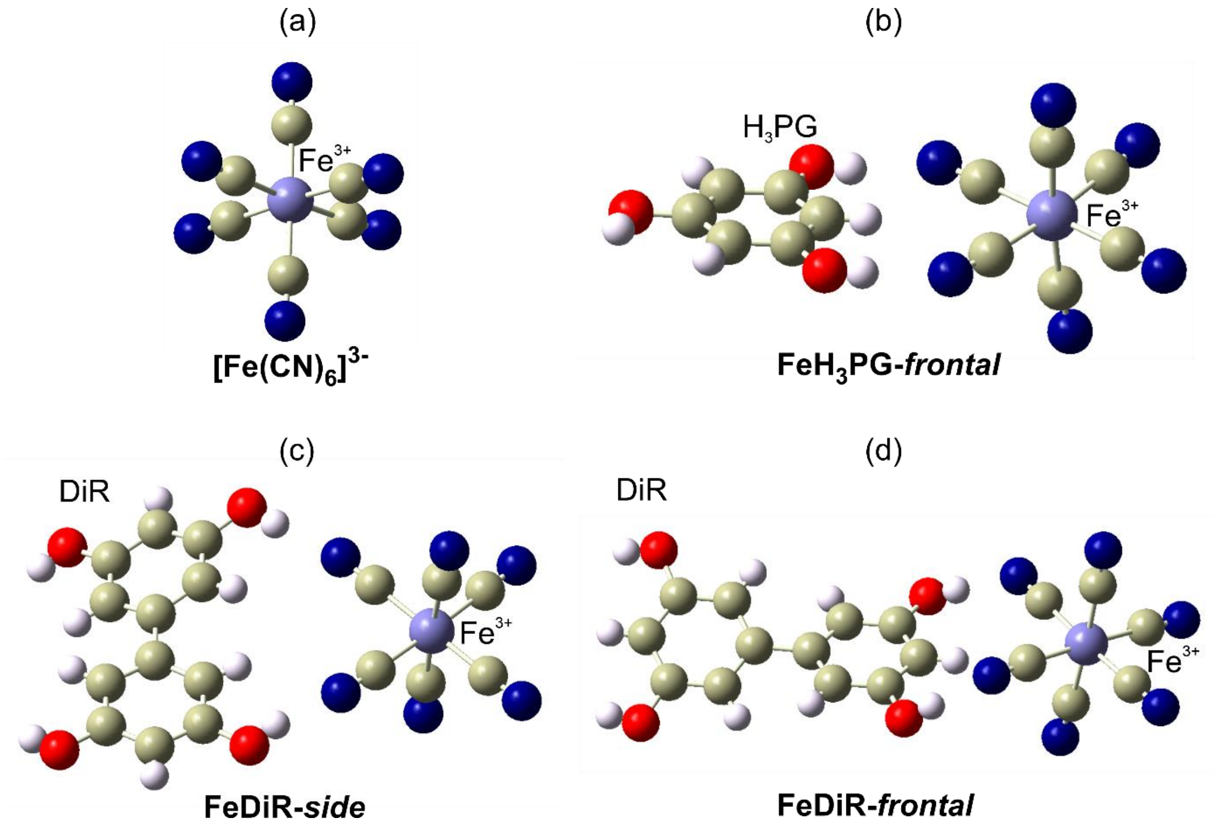

Figure S27. Molecular models used in calculations of UV-Vis electronic spectra representative of $[Fe(CN)_6]^{3-}$ references (a), **FeH$_3$PG** (b), and **FeDiR** (c, d).

Quantum-chemical calculations of the UV-Vis absorption spectra for the models presented in **Figure S27** were done using the Gaussian 16 software.[S12] The models were prepared based on the respective crystal structures by removing the solvent, counterion, and appropriate organic ligand molecules, to form the pristine $Fe(CN)_6^{3-}$ model, FeH$_3$PG-*frontal* model representative of the hydrogen-bonded contacts observed in the crystal structure of **FeH$_3$PG**,[S2] and two – FeDiR-*side* and FeDiR-*frontal* – models representative of the hydrogen-bonded contacts observed in the crystal structure of **FeDiR** (**Figure S27**). The single-point DFT and TD-DFT calculations were performed with the PBE density functional,[S21] using the Karlsruhe type def2-TZVP basis set for Fe, C, N, and H atoms.[S15,S16] Vertical excitation energies from the ground state to the lowest 300 electronic excited states were calculated along with the corresponding oscillator strengths for respective electronic transitions. The simulated spectra shown were obtained using the GaussView 6.1.1 program[S22] using Gaussian-broadening with the implemented UV-Vis peak half-width at half height parameter of 0.333 eV. For the assignment of the selected key transitions, see **Figures S28-S31**. As the ground-state energy calculations for the corresponding molecular model of FeTriRB-*side* (representative of the hydrogen-bonded contacts observed in the crystal structure of **FeTriRB**) failed to reach SCF convergence at the DFT-PBE level of theory, we were not able to simulate its UV-Vis spectrum using the above protocol. However, based on the results shown in **Figure 5** (main text) and **Figures S28-S31**, we believe that we might infer similar spectral properties for this system to those for **FeDiR**.

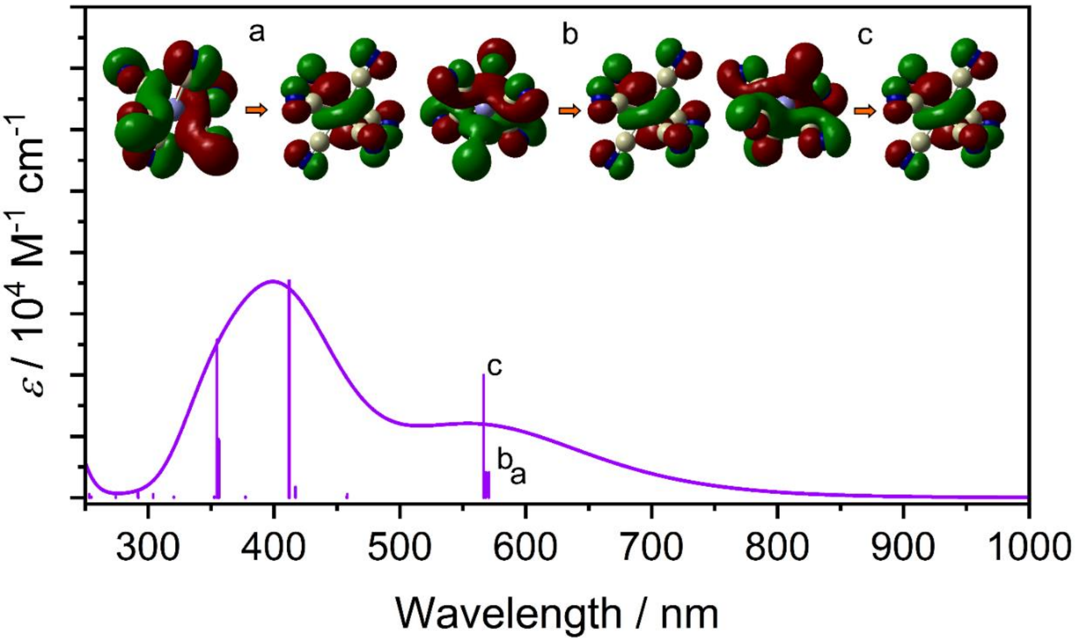

Figure S28. Simulated UV-Vis spectrum for the pristine $[Fe(CN)_6]^{3-}$ model. The bars indicate the energies and oscillator strengths for the electronic excitations computed in the presented spectral region. The analysed excitations a-c are assigned as intramolecular LMCT[2] transitions in $[Fe(CN)_6]^{3-}$, judging by the isosurfaces of the involved molecular orbitals.

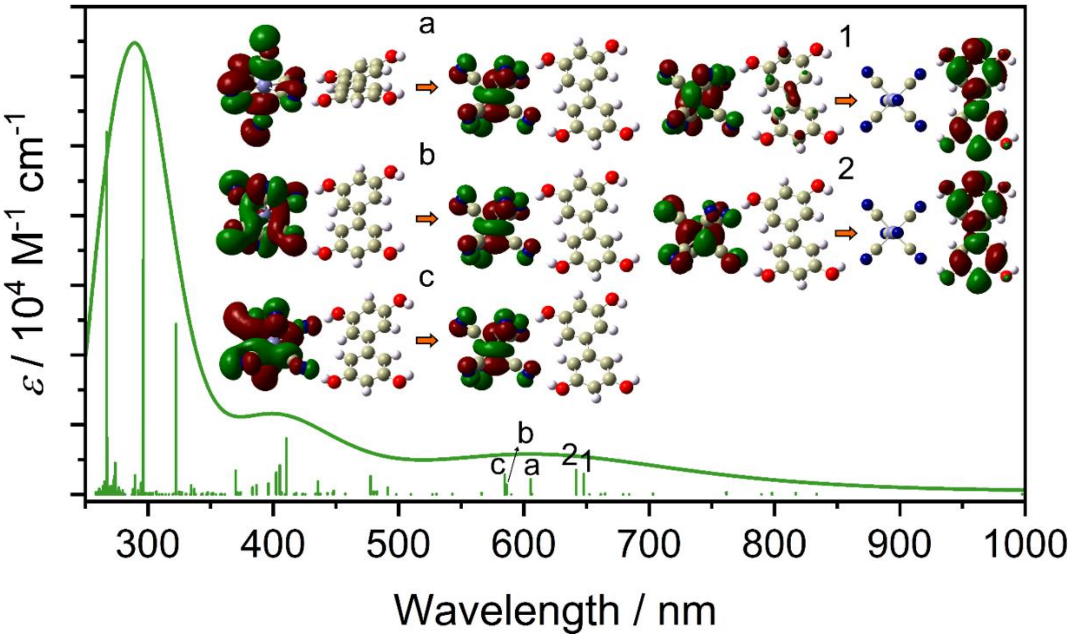

Figure S29. Simulated UV-Vis spectrum for the FeDiR-*side* model. The bars indicate the energies and oscillator strengths for the electronic excitations computed in the presented spectral region. The analysed excitations a-c are assigned as intramolecular LMCT[2] transitions in $[Fe(CN)_6]^{3-}$, whereas the excitations 1 and 2 correspond to intermolecular OSCT $[Fe(CN)_6]^{3-} \rightarrow$ DiR transitions, judging by the isosurfaces of the involved molecular orbitals. Note that the OSCT transitions are of lower energy compared to the LMCT transitions.

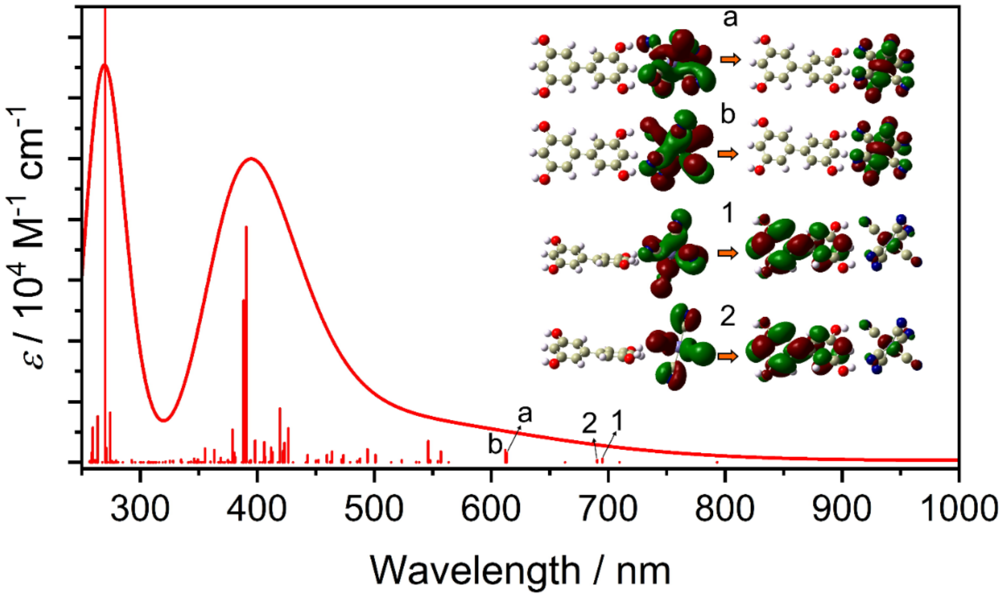

Figure S30. Simulated UV-Vis spectrum for the FeDiR-*frontal* model. The bars indicate the energies and oscillator strengths for the electronic excitations computed in the presented spectral region. The analysed excitations a and b are assigned as intramolecular LMCT[2] transitions in $[Fe(CN)_6]^{3-}$, whereas the excitations 1 and 2 correspond to intermolecular OSCT $[Fe(CN)_6]^{3-} \rightarrow$ DiR transitions, judging by the isosurfaces of the involved molecular orbitals. Note that the OSCT transitions are of lower energy compared to the LMCT transitions.

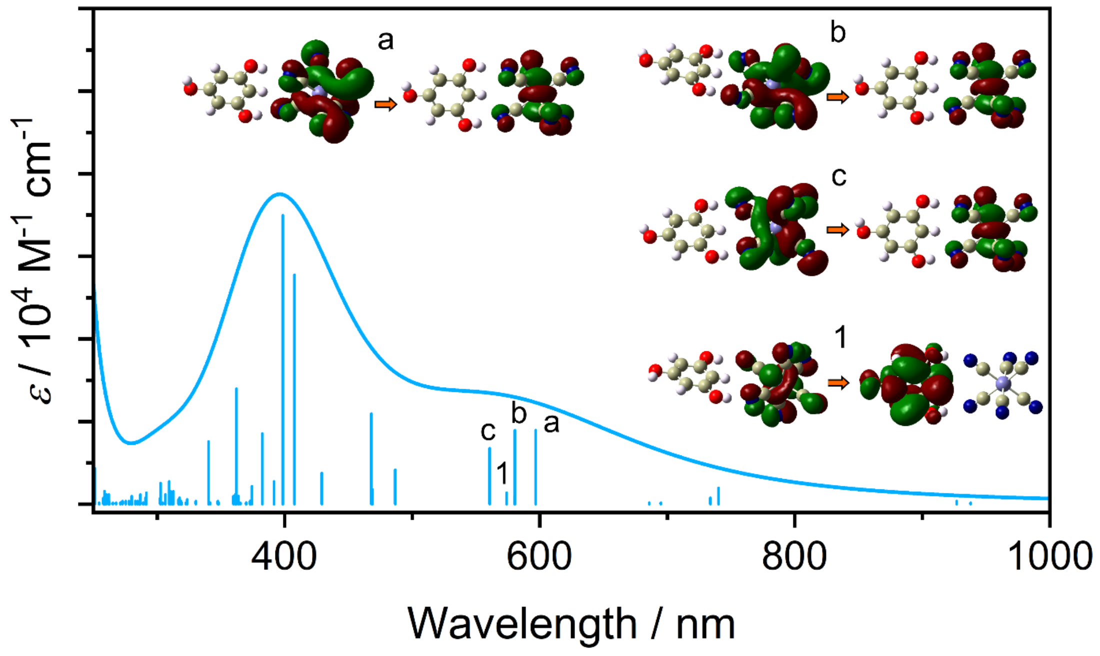

Figure S31. Simulated UV-Vis spectrum for the FeH$_3$PG-*frontal* model. The bars indicate the energies and oscillator strengths for the electronic excitations computed in the presented spectral region. The analysed excitations a-c are assigned as intramolecular LMCT[2] transitions in $[Fe(CN)_6]^{3-}$, whereas the excitation 1 corresponds to intermolecular OSCT $[Fe(CN)_6]^{3-} \rightarrow$ H$_3$PG transition, judging by the isosurfaces of the involved molecular orbitals. Note that in this case the OSCT transition is of comparable energy to the LMCT transitions, unlike in the case of FeDiR-*side* and FeDiR-*frontal*.

4.6. Second-harmonic generation studies

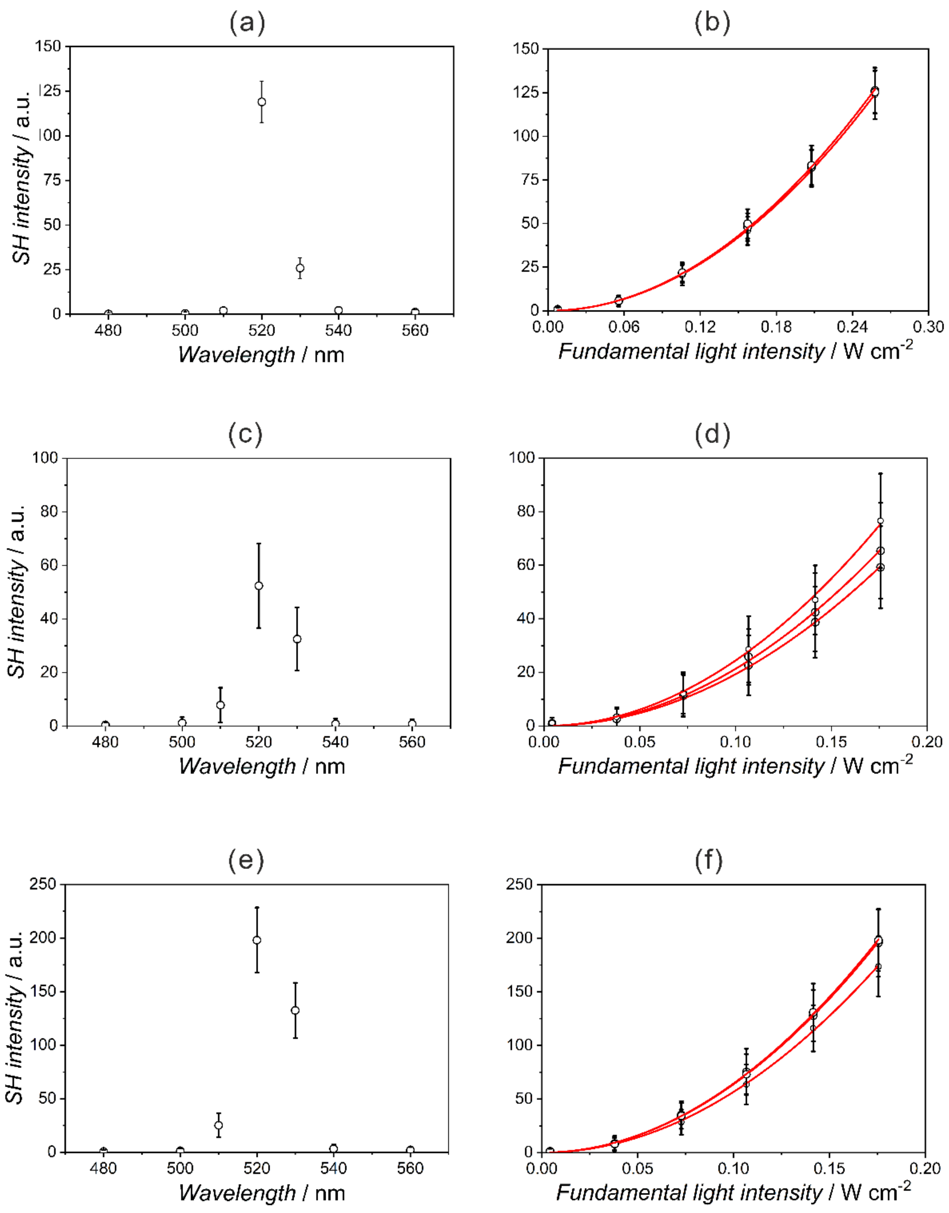

(g)

$$\begin{pmatrix} P_x \\ P_y \\ P_z \end{pmatrix} = \begin{pmatrix} 0 & 0 & 0 & 0 & \chi_{xzx} & 0 \\ 0 & 0 & 0 & \chi_{yyz} & 0 & 0 \\ \chi_{zxx} & \chi_{zyy} & \chi_{zzz} & 0 & 0 & 0 \end{pmatrix} \begin{pmatrix} E_x E_x \\ E_y E_y \\ E_z E_z \\ 2E_y E_z \\ 2E_z E_x \\ 2E_x E_y \end{pmatrix}$$

Figure S32. Second-harmonic signals for **CrDiR** (a), (b), (g), **FeDiR** (c), (d), (g), and **CoDiR** (e), (f), (g). SHG plotted against wavelength to confirm the chromaticity aberration of the SH signal of **CrDiR** (a), **FeDiR** (c), and **CoDiR** (e). Average SHG signals of **CrDiR** (b), **FeDiR** (d), and **CoDiR** (f); red solid lines correspond to the results of fitting using a quadratic function ($y = Ax^2$). Relation between tensor elements of the SH susceptibility corresponding to space group $Pna2_1$ and SH polarization (g).

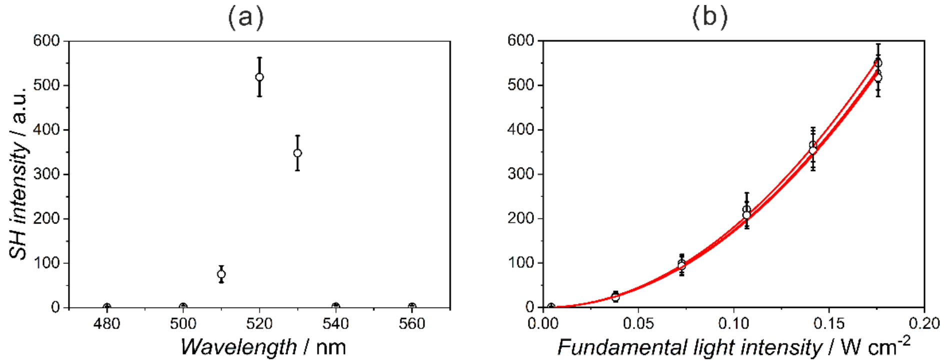

(c)

$$\begin{pmatrix} P_x \\ P_y \\ P_z \end{pmatrix} = \begin{pmatrix} \chi_{xxx} & \chi_{xyy} & \chi_{xzz} & 0 & 0 & \chi_{xxy} \\ \chi_{yxx} & \chi_{yyy} & \chi_{yzz} & 0 & 0 & \chi_{yxy} \\ 0 & 0 & 0 & \chi_{zyz} & \chi_{zzx} & 0 \end{pmatrix} \begin{pmatrix} E_xE_x \\ E_yE_y \\ E_zE_z \\ 2E_yE_z \\ 2E_zE_x \\ 2E_xE_y \end{pmatrix}$$

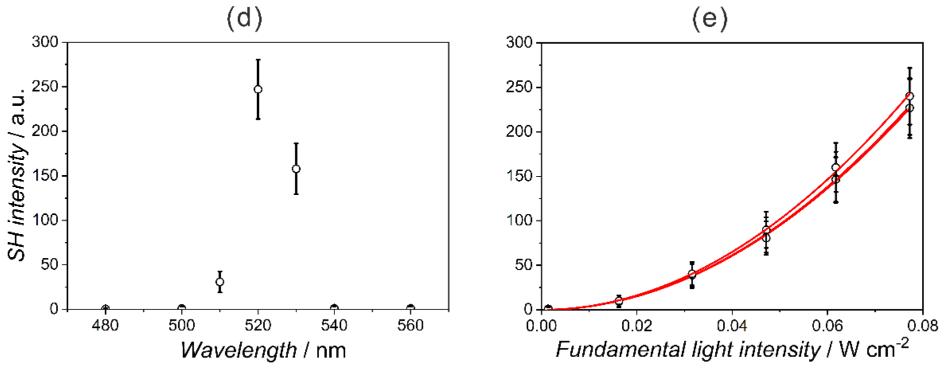

(f)

$$\begin{pmatrix} P_x \\ P_y \\ P_z \end{pmatrix} = \begin{pmatrix} 0 & 0 & 0 & \chi_{xyz} & 0 & 0 \\ 0 & 0 & 0 & 0 & \chi_{yzx} & 0 \\ 0 & 0 & 0 & 0 & 0 & \chi_{zxy} \end{pmatrix} \begin{pmatrix} E_xE_x \\ E_yE_y \\ E_zE_z \\ 2E_yE_z \\ 2E_zE_x \\ 2E_xE_y \end{pmatrix}$$

Figure S33. Second-harmonic signals for **CrTriRB** (a), (b), (c), and **FeTriRB** (d), (e), (f). SHG plotted against wavelength to confirm the chromaticity aberration of the SH signal of **CrTriRB** (a) and **FeTriRB** (d). Average SHG signals of **CrTriRB** (b) and **FeTriRB** (e); red solid lines correspond to the results of fitting using a quadratic function ($y = Ax^2$). Relation between tensor elements of the SH susceptibility corresponding to space group *Cc* and $P2_12_12$ and SH polarization, respectively, (c) and (f).

Table S15. Room-temperature second-harmonic susceptibilities for powdered samples of **MDiR** and **MTriRB**, the KDP standard, and other reference assemblies.

Compound	Topology	Space group	SH susceptibility (esu) (% KDP)	Reference
**CrDiR**	Ionic: cocrystal salt solvates	$Pna2_1$	6.0×10^{-12} (0.5%)	This work
**FeDiR**		$Pna2_1$	7.2×10^{-12} (0.6%)	This work
**CoDiR**		$Pna2_1$	2.2×10^{-11} (1.8%)	This work
**CrTriRB**		Cc	6.2×10^{-11} (5.2%)	This work
**FeTriRB**		$P2_12_12$	3.2×10^{-11} (2.7%)	This work
**CoTriRB**		$P2_1$	1.4×10^{-10} (11.7%)	This work
KH_2PO_4 (KDP)	Ionic salt	$P2_1$	1.2×10^{-9} (100%)	S23
$[Dy(phen)_2(NO_3)(H_2O)][Fe(CN)_5(NO)]\cdot3H_2O$	1D	$Pna2_1$	3.5×10^{-10} (30%)	S24
$\{[Cu(NH_3)_2]_2[Mo(CN)_8]\}$	3D	$Fdd2$	1.8×10^{-10} (15%)	S25
$\{[Gd(dmf)_6][Mo(CN)_5(NO)]\}$	1D	$Pna2_1$	1.2×10^{-10} (10%)	S26
$[Fe_2[Nb(CN)_8](R\text{-}/S\text{-}pEtOH)_8\cdot6H_2O$	3D	$I2_13$	1.1×10^{-10} (9.2%)	S27
$[Dy(dma)_5][W(CN)_8]$	1D	$P2_1$	9.4×10^{-11} (7.8%)	S28
$[Tb(dma)_5][W(CN)_8]$	1D	$P2_1$	8.9×10^{-11} (7.4%)	S28
$Rb_{0.94}Mn[Fe(CN)_6]_{0.98}\cdot0.2H_2O$	3D	$F\bar{4}3m$	8×10^{-11} (6.7%)	S29
$[Er(dma)_5][W(CN)_8]$	1D	$P2_1$	6.0×10^{-11} (5.0%)	S28,S30
$[Mn(pz)(H_2O)_2][Mn(H_2O)_2][Mo(CN)_8]\cdot4H_2O$	3D	$P2_1$	6×10^{-11} (5.0%)	S31
$[Y(dma)_5][W(CN)_8]$	1D	$P2_1$	4.9×10^{-11} (4.1%)	S28
$[Ho(dma)_5][W(CN)_8]$	1D	$P2_1$	2.5×10^{-11} (2.1%)	S28

$[Gd(dma)_5][W(CN)_8]$	1D	$P2_1$	2.2×10^{-11} (1.8%)	S28
$[Mn(pz)(H_2O)_2][Mn(H_2O)_2][Nb(CN)_8]\cdot4H_2O$	3D	$P2_1$	2×10^{-11} (1.7%)	S32
$[Tm(dma)_5][W(CN)_8]$	1D	$P2_1$	5.5×10^{-12} (0.5%)	S28
$[Yb(dma)_5][W(CN)_8]$	1D	$P2_1$	4.9×10^{-12} (0.4%)	S28
$[Lu(dma)_5][W(CN)_8]$	1D	$P2_1$	4.7×10^{-12} (0.4%)	S28
$\{[Nd_4(H_2O)_{17}(pzdo)_5][W(CN)_8]_3\}\cdot9H_2O$	2D	$C2$	2.6×10^{-12} (0.22%)	S33
$\{[Nd_4(H_2O)_{17}(pzdo)_5][Mo(CN)_8]_3\}\cdot9H_2O$	2D	$C2$	1.3×10^{-12} (0.11%)	S33

Ligands: dma = *N*,*N*-dimethylacetamide; dmf = *N*,*N*-dimethylformamide; pEtOH = 1-(3-pyridyl)ethanol; phen = 1,10-phenanthroline; pz = pyrazine; pzdo = pyrazine-*N*,*N′*-dioxide.

4.7. Photoluminescence studies

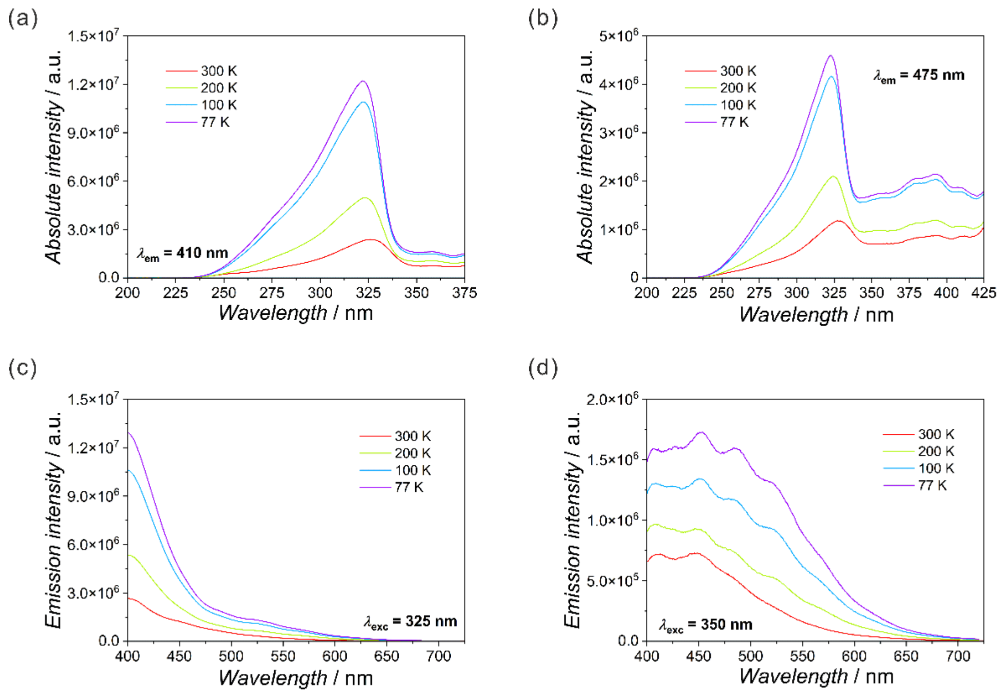

Figure S34. Photoluminescence spectra of DiR. Excitation spectra followed with the emission wavelength of 410 nm (a) and 475 nm (b). Emission spectra for the corresponding excitation wavelengths of 325 nm (c) and 350 nm (d).

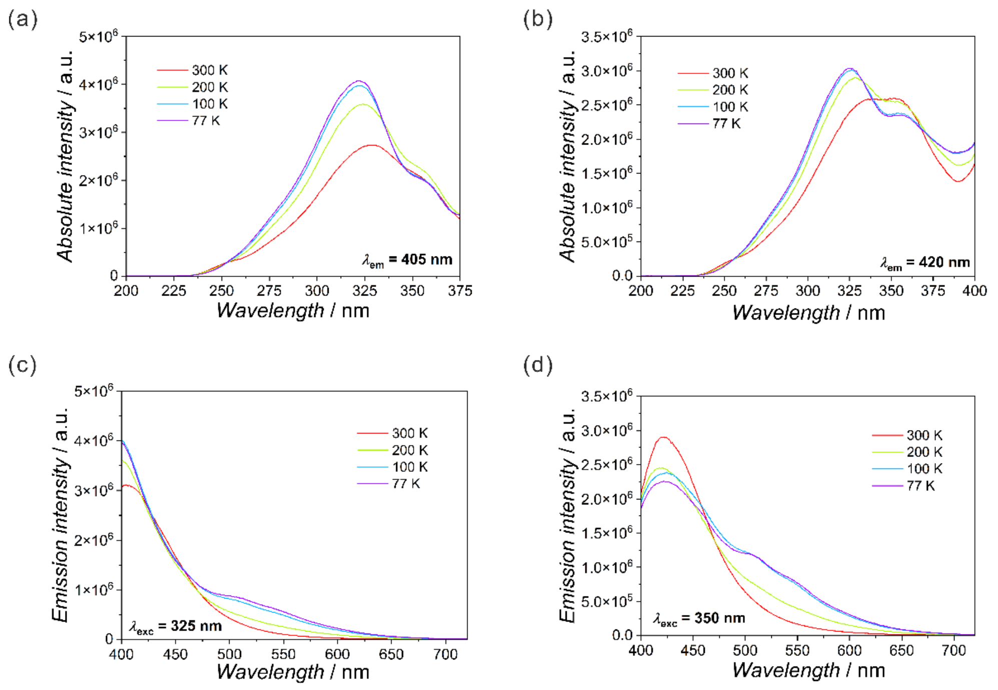

Figure S35. Photoluminescence spectra of TriRB. Excitation spectra followed with the emission wavelength of 405 nm (a) and 420 nm (b). Emission spectra for the corresponding excitation wavelengths of 325 nm (c) and 350 nm (d).

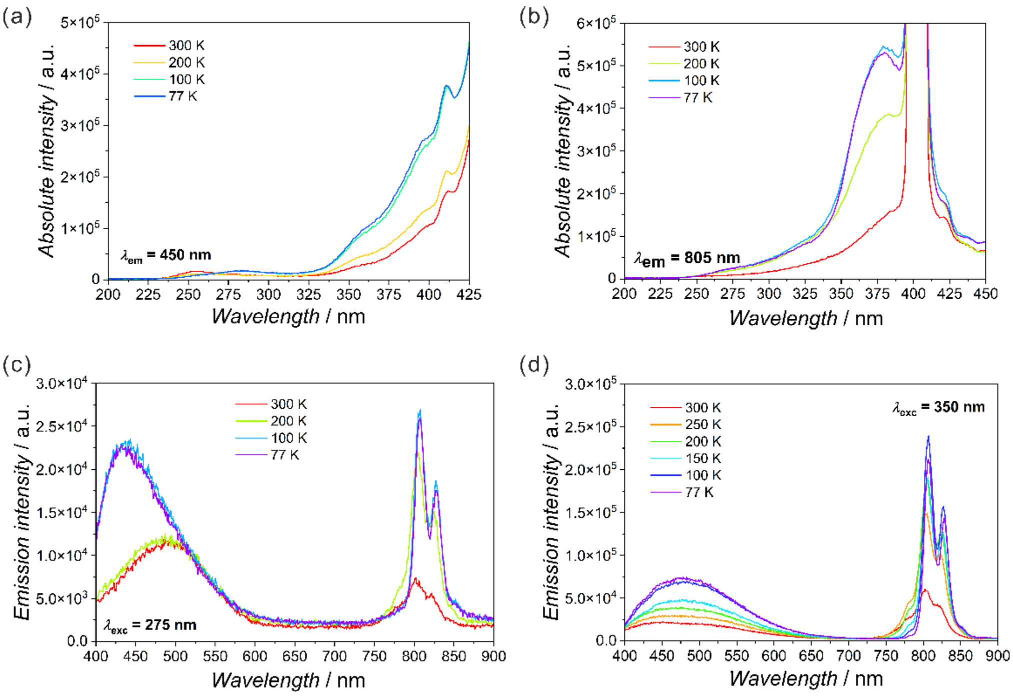

Figure S36. Photoluminescence spectra of **CrDiR**. Excitation spectra followed with emission wavelengths of 450 nm (a) and 805 nm (b). Emission spectra for the corresponding excitation wavelengths of 275 nm (c) and 350 nm (d).

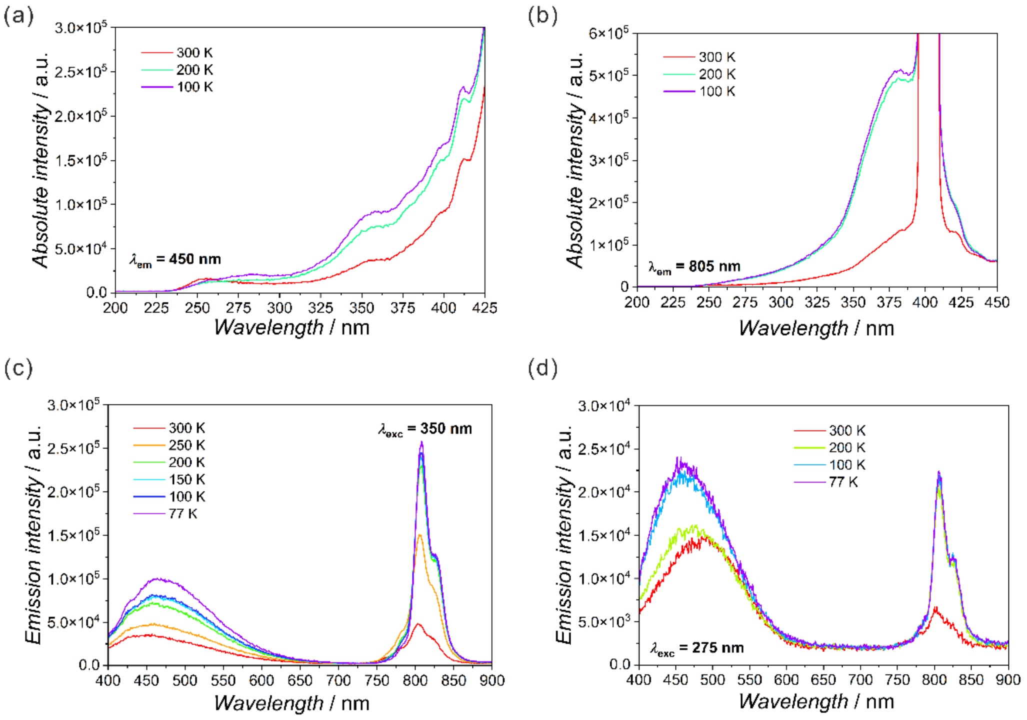

Figure S37. Photoluminescence spectra of **CrTriRB**. Excitation spectra followed with emission wavelengths of 450 nm (a) and 805 nm (b). Emission spectra for the corresponding excitation wavelengths of 350 nm (c) and 275 nm (d).

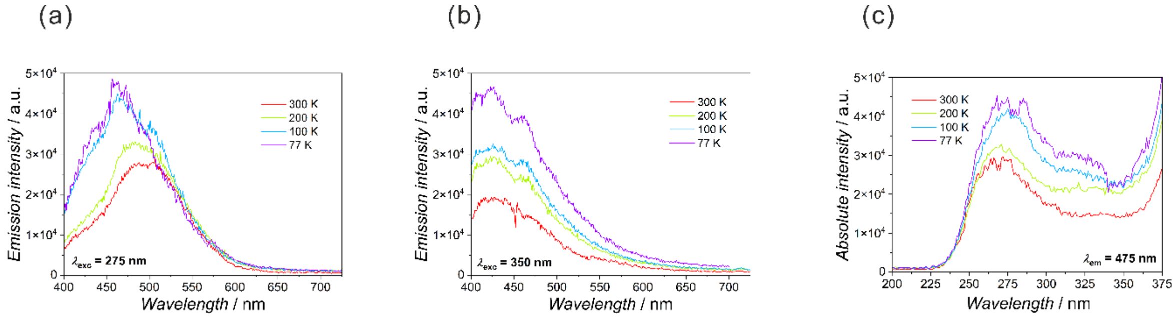

Figure S38. Photoluminescence spectra of **FeDiR**. Emission spectra for the corresponding excitation wavelengths of 275 nm (a) and 350 nm (b). Excitation spectra followed with emission wavelength of 475 nm (c).

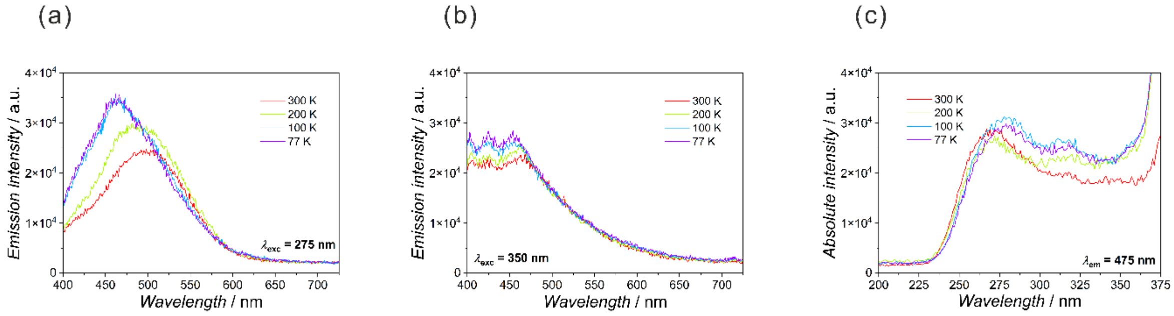

Figure S39. Photoluminescence spectra of **FeTriRB**. Emission spectra for the corresponding excitation wavelengths of 275 nm (a) and 350 nm (b). Excitation spectra followed with emission wavelength of 475 nm (c).

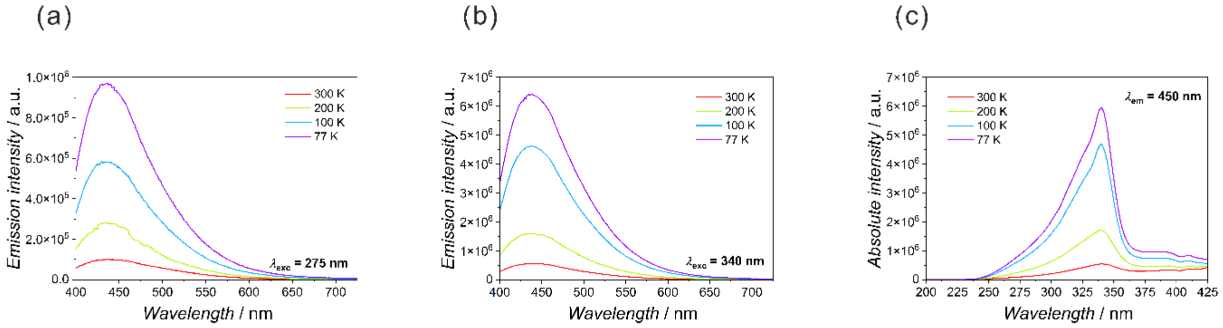

Figure S40. Photoluminescence spectra of **CoDiR**. Emission spectra for the corresponding excitation wavelengths of 275 nm (a) and 340 nm (b). Excitation spectra followed with emission wavelength of 450 nm (c).

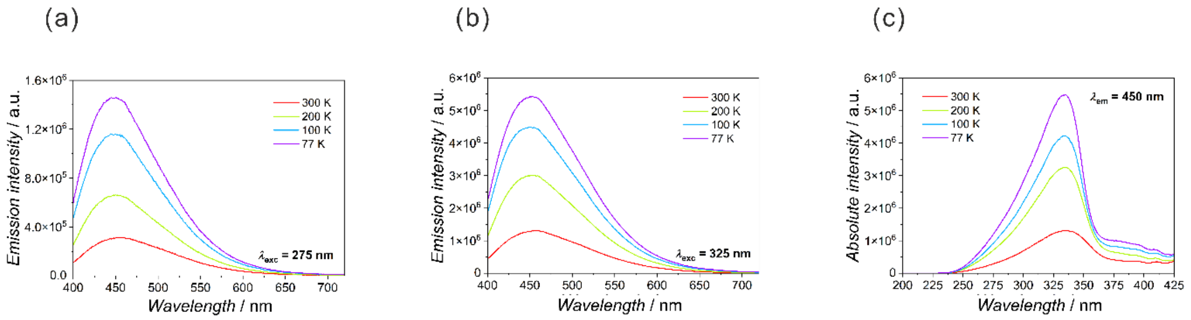

Figure S41. Photoluminescence spectra of **CoTriRB**. Emission spectra for the corresponding excitation wavelengths of 275 nm (a) and 325 nm (b). Excitation spectra followed with emission wavelength of 450 nm (c).

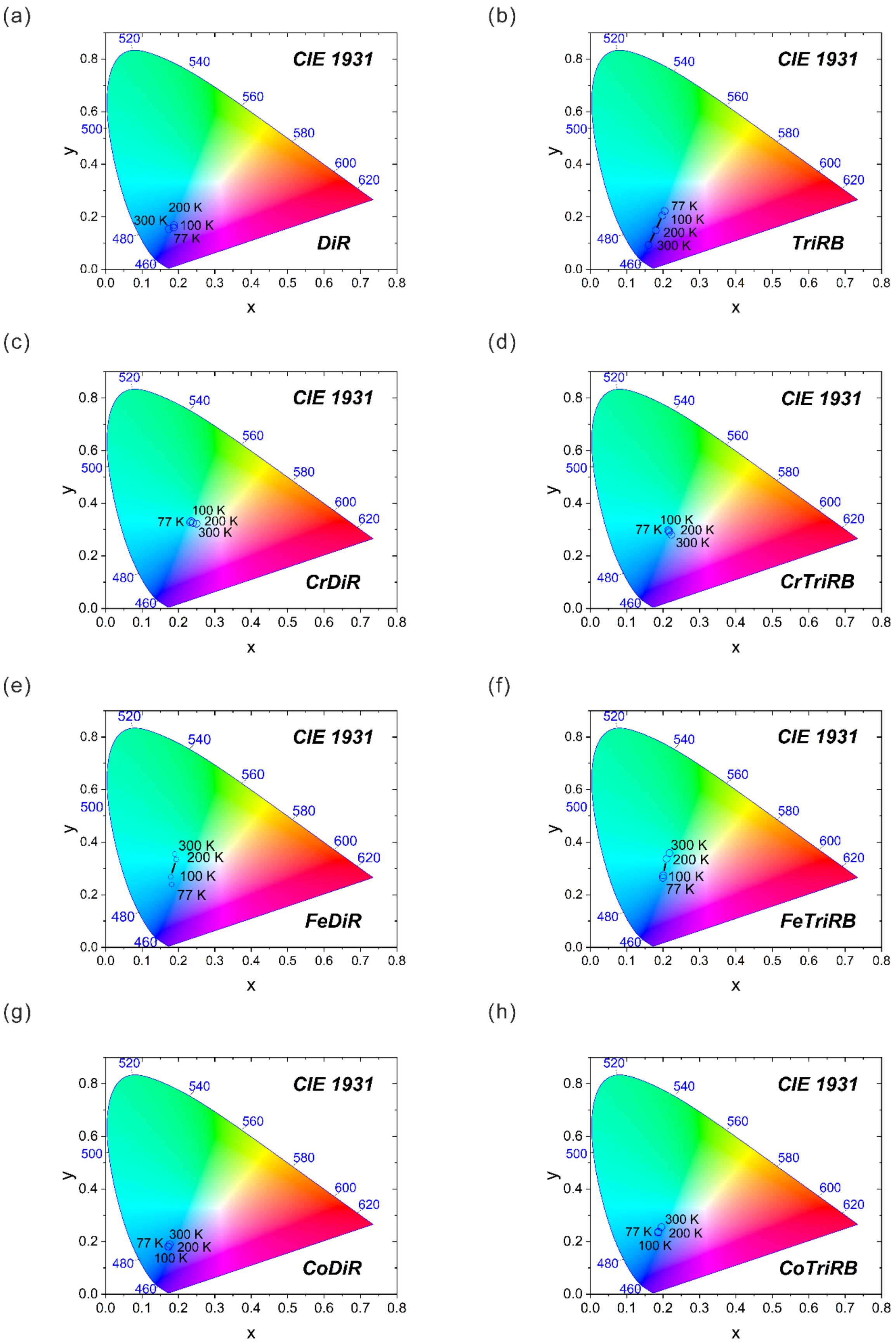

Figure S42. CIE1931 uniform chromaticity scale diagrams for DiR (a) and TriRB (b) ligands, **CrDiR** (c), **CrTriRB** (d), **FeDiR** (e), **FeTriRB** (f)**, CoDiR** (g), and **CoTriRB** (h).

Table S16. Values of the measured quantum yield (QY) for **CrDiR**, **CrTriRB**, **CoDiR**, and **CoTriRB** together with the corresponding data for the coformers DiR and TriRB as the references.

Compound	Quantum Yield, QY	Excitation Wavelength
**CrDiR**	8.9%	375 nm
**CrTriRB**	2.4%	375 nm
**CoDiR**	1.7%	325 nm
**CoTriRB**	1.3%	325 nm
DiR	0.6%	325 nm
TriRB	0.1%	325 nm

4.8. Details of materials and methods

Materials and syntheses

Synthetic procedures for precursors

$(PPh_4)_3[Fe(CN)_6]\cdot 7H_2O$, $(PPh_4)_3[Co(CN)_6]\cdot 6H_2O$, and $(PPh_4)_3[Cr(CN)_6]\cdot 2H_2O$ were prepared by metathesis of the corresponding potassium salts of the complexes with PPh_4Cl. 3,3',5,5'-Tetramethoxy-1,19-biphenyl – DiR^{OMe} and 5'-(3,5-dimethoxyphenyl)-3,3",5,5"-tetramethoxy-1,19:3',1"-terphenyl – TriRB^{OMe} were obtained using the Suzuki–Miyaura reaction, where the coupling partners were a boronic acid and an organohalide, and the catalyst was a palladium(0) complex. DiR^{OMe} and TriRB^{OMe} unlocking resulted in 3,3',5,5'-tetrahydroxy-1,19-biphenyl – DiR and 5'-(3,5-dihydroxyphenyl)-3,3",5,5"-tetrahydroxy-1,19:3',1"-terphenyl – TriRB according to a modified literature procedure.[S34]

*General Synthetic Procedure for **MDiR** and **MTriRB***

Acetonitrile solutions of $(PPh_4)_3[M(CN)_6]\cdot nH_2O$ (M = Cr(III), Fe(III), Co(III); n = 2, 6, 7) (0.026 g, ~ 0.019 – 0.021 mmol in 1 mL of MeCN) and DiR or TriRB (0.018 mmol in 1 mL of MeCN) were sonicated to obtain a clear solution; they were then combined, and the cream-colored solid was obtained, followed by dissolution in MeOH. The clear solution in a vial was exposed to diethyl ether vapors in a closed container. After 2 days, the crystals were filtered and washed with cold MeCN (10 mL, 2 °C) and dried in the air. The crystal structures of all compounds were determined based on single-crystal X-ray diffraction experiments. Fresh crystalline compounds were used for elemental analysis, infrared (IR), Raman, and UV-Vis absorption spectroscopies, SHG measurement, powder X-ray diffraction (PXRD), and thermogravimetric analyses.

*Synthesis of **CrDiR** - $(PPh_4)_3[Cr(CN)_6](DiR)_2\cdot 2MeCN\cdot MeOH$*

Yield: 0.029 g, 80.0%. Elemental analysis: Calc. for $C_{107}H_{90}CrN_8O_9P_3$ (M_w = 1776.77 g·mol^{-1}): C, 72.33%; H, 5.11%; N, 7.18%. Found: C, 72.3%; H, 5.1%; N, 7.1%. IR (KBr, cm^{-1}): 3410 – 2662 ν(O-H), 3082 ν(C-H), 2131 ν(C≡N) ($[Cr(CN)_6]^{3-}$), other: 1601, 1521, 1484, 1437, 1306, 1188, 1151, 1110, and 998 (vibrations of PPh_4^+), 843, 755, and 724 (vibrations of DiR). Solubility: MeOH–good. Stability: composition is stable up to ca. 20 °C, the loss of 1 crystallization MeCN and 0.5 crystallization MeOH molecules occurs above room temperature; then, above 240 °C massive decomposition occurs (Figure S43).

*Synthesis of **FeDiR** - $(PPh_4)_3[Fe(CN)_6](DiR)_2\cdot 3MeCN$*

Yield: 0.029 g, 85.0%. Elemental analysis: Calc. for $C_{108}H_{89}FeN_9O_8P_3$ (M_w = 1789.64 g·mol^{-1}): C, 72.48%; H, 5.01%; N, 7.04%. Found: C, 72.3%; H, 4.9%; N, 7.1%. IR (KBr, cm^{-1}): 3062 – 2661 ν(O-H)/ν(C-H), 2117 ν(C≡N) ($[Fe(CN)_6]^{3-}$), other: 1590, 1483, 1442, 1412, 1352, 1316, 1228, 12144, 1175, 1152, 1109, and 1007 (vibrations of PPh_4^+), 843 and 756 (vibrations of DiR). Solubility: MeOH–good. Stability: composition is stable up to ca. 20 °C, the loss of 1.5 crystallization MeCN molecules occurs slowly above room temperature; then, above 230 °C massive decomposition occurs (Figure S43).

*Synthesis of **CoDiR** - $(PPh_4)_3[Co(CN)_6](DiR)_2\cdot MeCN\cdot 2MeOH$*

Yield: 0.028 g, 85.0%. Elemental analysis: Calc. for $C_{106}H_{91}CoN_7O_{10}P_3$ (M_w = 1774.69 g·mol^{-1}): C, 71.74%; H, 5.17%; N, 5.52%. Found: C, 71.6%; H, 5.1%; N, 5.5%. IR (KBr, cm^{-1}): 3092 – 2664 ν(O-H), 2132 and 2121

v(C≡N) ($[Co(CN)_6]^{3-}$), other: 1601, 1586, 1484, 1466, 1443, 1437, 1304, 1266, 1218, 1168, 1152, 1110, and 1004 (vibrations of PPh_4^+), 840, 756, and 724 (vibrations of DiR). Solubility: MeOH. Stability: composition is stable up to ca. 60 °C, the loss of 1 crystallization MeCN and 2 crystallization MeOH molecules is observed in the range of 60–150 °C; then, above 290 °C massive decomposition occurs (Figure S43).

*Synthesis of **CrTriRB** - $(PPh_4)_3[Cr(CN)_6](TriRB)$·MeCN·solv*

Yield: 0.019 g, 53.0%. Elemental analysis: Calc. for $C_{107}CrH_{93}N_7O_9P_3$ (M_w = 1765.79 g·mol^{-1}): C, 72.33%; H, 5.11%; N, 7.18%. Found: C, 72.3%; H, 5.1%; N, 7.1%. IR (KBr, cm^{-1}): 3410 – 2662 *v*(O-H), 3082 *v*(C-H), 2131 *v*(C≡N) ($[Cr(CN)_6]^{3-}$), other: 1601, 1521, 1484, 1437, 1306, 1188, 1151, 1110, and 998 (vibrations of PPh_4^+), 843, 755, and 724 (vibrations of TriRB). Solubility: MeOH. Stability: composition is stable up to ca. 40 °C, the loss of crystallization solvent molecules is observed in the range of 40–190 °C; then, above 230 °C massive decomposition occurs (Figure S43).

*Synthesis of **FeTriRB** - $(PPh_4)_3[Fe(CN)_6](TriRB)$·2MeCN·MeOH*

Yield: 0.016 g, 46.0%. Elemental analysis: Calc. for $C_{107}H_{88}FeN_8O_7P_3$ (M_w = 1746.61 g·mol^{-1}): C, 73.58%; H, 5.08%; N, 6.42%. Found: C, 73.5%; H, 5.0%; N, 6.4%. IR (KBr, cm^{-1}): 3060 – 2662 *v*(O-H)/*v*(C-H), 2116 *v*(C≡N) ($[Fe(CN)_6]^{3-}$), other: 1590, 1483, 1442, 1352, 1316, 1174, 1151, 1109, 1007, and 996 (vibrations of PPh_4^+), 842, 755, and 723 (vibrations of TriRB). Solubility: MeOH. Stability: composition is stable up to ca. 50 °C, the loss of 1 crystallization MeCN molecule occurs in the range of 50–150 °C; then, above 230 °C massive decomposition occurs (Figure S43).

*Synthesis of **CoTriRB** - $2[(PPh_4)_3[Co(CN)_6](TriRB)]$·4MeCN·1MeOH*

Yield: 0.017 g, 52.0%. Elemental analysis: Calc. for $C_{213}H_{172}Co_2N_{16}O_{13}P_6$ (M_w = 3467.34 g·mol^{-1}): C, 73.78%; H, 5.00%; N, 6.46%. Found: C, 73.6%; H, 5.1%; N, 6.5%. IR (KBr, cm^{-1}): 3061 – 2672 *v*(O-H)/*v*(C-H), 2138 *v*(C≡N) ($[Co(CN)_6]^{3-}$), other: 1588, 1484, 1437, 1341, 1164, 1109, 1009, and 997 (vibrations of PPh_4^+), 847, 753, and 723 (vibrations of TriRB). Solubility: MeOH. Stability: composition is stable up to ca. 30 °C, the loss of 4 crystallization MeCN and 1 crystallization MeOH molecules is observed in the range of 30–100 °C; then, above 310 °C massive decomposition occurs (Figure S43).

4.9. Thermogravimetric analysis

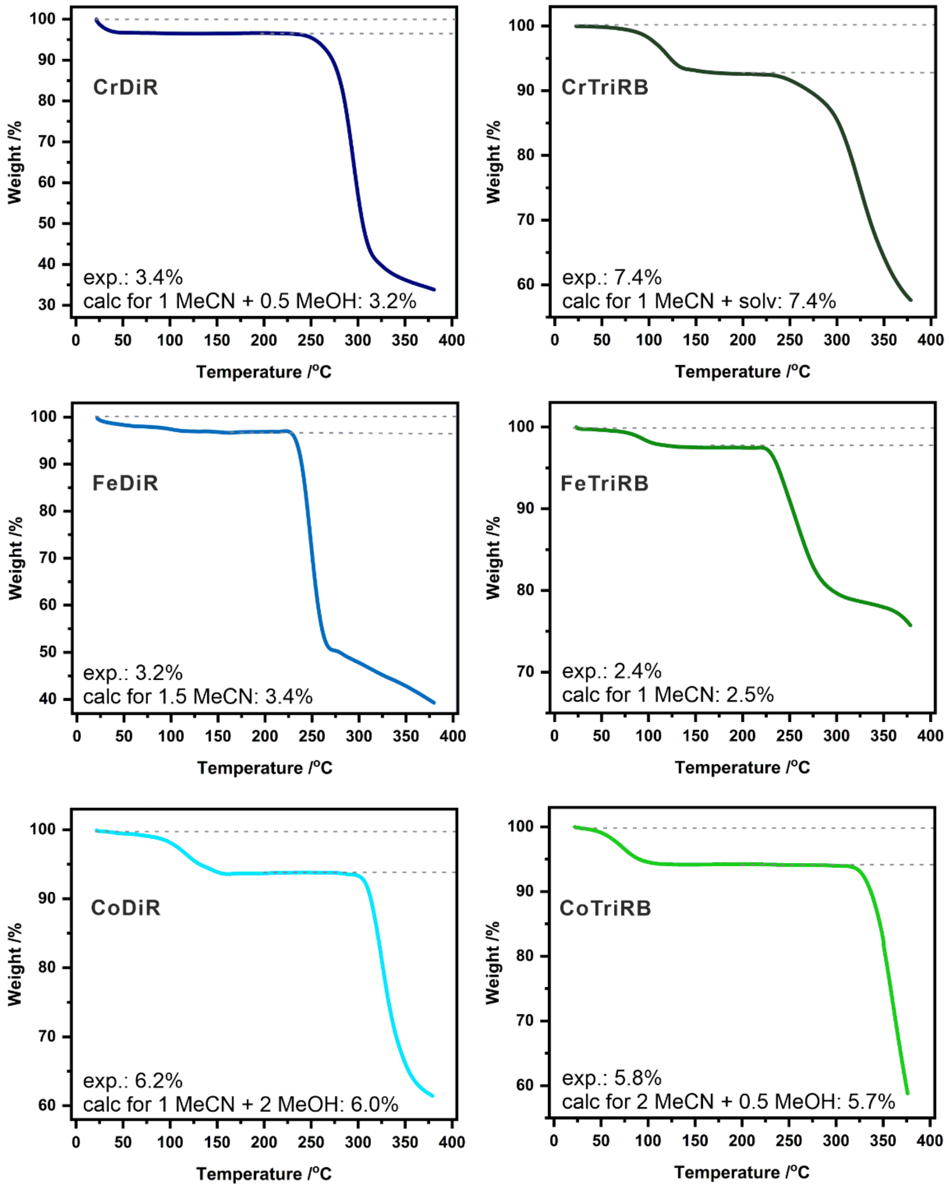

Figure S43. Thermogravimetric curves of **MDiR** and **MTriRB** collected under nitrogen atmosphere with a heating rate of 10 K per minute. The graphs show separate TG curves for each compound (**CrDiR**, **FeDiR**, **CoDiR**, **CrTriRB**, **FeTriRB**, and **CoTriRB**). Upon heating under nitrogen atmosphere, the powder samples are stable to T = 20°C (**CrDiR**, **FeDiR**, **CrTriRB**), 30°C (**CoTriRB**), and 50°C (**CoDiR**, **FeTriRB**), then exhibit a small decrease in mass up to T = 140°C (**CrDiR**, **FeTriRB**, **CoTriRB**), 190°C (**CrTriRB**), 200°C (**FeDiR**), and 250°C (**CoDiR**). These steps related to the loss of acetonitrile and methanol solvent molecules were depicted on the graphs, and they are accompanied by a comparison with the calculated values for the presented amount of solvent molecules per $[M(CN)_6]^{3-}$ units. The related decrease in the sample mass can also be correlated with the number of solvent molecules determined by CHN analyses (see synthetic procedures in the main text). Then, for the desolvated samples above T = 230°C (**FeDiR**, **CrTriRB**, **FeTriRB**), 240°C (**CrDiR**), 290°C (**CoDiR**), and 310°C (**CoTriRB**) massive decomposition occurs.

X-ray diffraction analysis.

Diffraction data for all the compounds were collected using a Bruker D8 Quest Eco diffractometer equipped with a Photon II detector and a Mo Kα (λ = 0.71073 Å) radiation source with a graphite monochromator and Oxford Cryostream cooling system. Crystals for measurement were taken from the mother solution and covered by NVH immersion oil. All measurements were performed at 100 K. Data reduction and cell parameter refinement were carried out using the Apex software, which included the SAINT and SADABS programs. The intensities of reflections for the sample absorption were corrected using the multiscan method. Structures were solved by the intrinsic phasing method and refined anisotropically with weighted full-matrix least-squares on F2 using the SHELXTS[35] and SHELXLS[36] programs with the Olex 2 graphic interface.[S37] Heavy atoms were refined with anisotropic displacement parameters, whereas hydrogen atoms were assigned at calculated positions with thermal displacement parameters fixed to a value of 20% or 50% higher than those of the corresponding carbon atoms. All structural figures were prepared using the Mercury software.[S38] The results of the data collection and refinement have been summarized in Tables S1 and S2, and selected bond lengths and angles are presented in Tables S3 and S4 in the SI. CCDC **CrDiR** – 2271018, **FeDiR** – 2271019, **CoDiR** – 2271020, **CrTriRB** – 2271021, **FeTriRB** – 2271022, and **CoTriRB** – 2271023 contain the supplementary crystallographic data for all the compounds, respectively. These data can be obtained free of charge from the Cambridge Crystallographic Data Centre www.ccdc.cam.ac.uk.data_request/cif. The topology of hydrogen-bonded subnetworks was analyzed using the TOPOS Pro software.[S39]

Physical techniques and computational methods.

Elemental analyses of CHNS were performed using the Elemental Vario Micro Cube CHNS analyzer. Powder X-ray diffraction patterns for **MDiR**, and **MTriRB** in 0.7 mm glass capillaries were collected on a D8 Advance Eco (Bruker) using a Cu−Kα radiation source. Thermogravimetric (TGA) curves for the polycrystalline samples were collected using a TG209 F1 Libra thermogravimetric analyzer with aluminum pans as holders. The data were collected in the temperature range of 21-400 °C under a nitrogen atmosphere with a heating rate of 10 ºC per minute. Infrared (IR) absorption spectra in the range of 4000-675 cm^{-1} were measured on the selected single-crystals using a Nicolet iN10 MX Fourier transform infrared microscope. Diffuse reflectance spectra measurements in the UV-Vis-NIR range were performed for the freshly ground powder samples mixed with $BaSO_4$ (2 mass %) using a Shimadzu UV-3600i Plus spectrophotometer equipped with the 50 mm integrating sphere. The spectra were recalculated according to the Kubelka–Munk equation. Luminescence spectra in the reflectance mode were measured with the Horiba Jobin-Yvon Fluorolog®-3 (FL3-211) spectrofluorometer (model TKN-7), equipped with a 450 W Xe-lamp and the R928P detector working in the photon counting mode, for the samples dispersed in paraffin oil and blocked between two quartz plates placed inside the liquid nitrogen-cooled MicrostatHe (Oxford Instruments) connected to the digital temperature controller MercuryiTC (a sweeping rate of 2 K min^{-1}). Room-temperature Raman scattering spectra in the range of 4000-100 cm^{-1} for the single crystals were accumulated using a JASCO NRS-7500 laser Raman spectrometer (532.12 nm laser with 0.8 mW power, single monochromator, L600/B500 nm grating, d-100 μm slit, d-4000 μm aperture, MPLFLN 20x objective lens, BS 30/70, and 5%(OD1.3) attenuator). A room-temperature absolute photoluminescence quantum yield detection device (C9920-02, Hamamatsu Photonics) with an integrating sphere with a 3.3-inch radius was used to measure emission quantum yields. The excitation source, a Xenon lamp, produced different excitation wavelengths ranging from 250 to 950 nm with the use of a monochromator. A PMA multichannel spectrometer was used to detect the emitted light in

the reflection mode after normal excitation light exposure through the side of the sphere. To prevent seeing the sample's fluorescence and scattered light directly, a baffle was put in between the sample and the detecting port. An empty quartz vessel was inserted into the sphere prior to each measurement to serve as an excitation reference spectrum. The quantum yield of the sample was then determined by dividing the number of photons absorbed by the sample by the number of photons released as photoluminescence. For the same substance, several independent measurements were made to confirm its reproducibility. In SHG measurements, fundamental 1040 nm light, generated by the femtosecond pulsed laser (Spectra Physics Spirit One), has been applied. Green light transmitted through an IR filter, a third harmonic generation filter, and a band-pass filter was observed with a photomultiplier tube (Hamamatsu R329-02). The Mössbauer spectra of **FeDiR** and **FeTriRB** were recorded in a routine transmission geometry at room temperature, using WissEl spectrometer (Wissenschaftliche Electronic GmbH, Ortenberg, Germany). The powder samples were directly placed into copper rings and sealed with Kapton foil. The spectra were collected in 1024 channels, with a ~5 mCi ^{57}Co source in an Rh matrix. The temperature stabilization was up to 0.5 K. The velocity scale was calibrated using the α-Fe foil standard. The reported spectra were numerically evaluated with the use of the *WinNormos-for-Igor package* (Wissenschaftliche Electronic GmbH, Ortenberg, Germany).

References to Supplementary Information

(S1) Loos, M.; Gerber C.; Corona, F.; Hollander, J.; Singer H. Accelerated Isotope Fine Structure Calculation Using Pruned Transition Trees. *Anal. Chem.* **2015**, *87*, 5738–5744.

(S2) Jędrzejowska, K.; Kobylarczyk, J.; Glosz, D.; Kuzniak-Glanowska, E.; Tabor, D.; Srebro-Hooper, M.; Zakrzewski, J. J.; Dziedzic-Kocurek, K.; Muzioł, T. M.; Podgajny R. Supramolecular cis-“Bis(Chelation)” of $[M(CN)_6]^{3-}$ (M = Cr^{III}, Fe^{III}, Co^{III}) by Phloroglucinol (H_3PG). Molecules **2022**, *27*, 4111.

(S3) te Velde, G.; Bickelhaupt, F. M.; Baerends, E. J.; Fonseca Guerra, C.; van Gisbergen, S. J. A.; Snijders, J. G.; Ziegler, T. Chemistry with ADF. *J Comput Chem* **2001**, *22*, 931–967.

(S4) ADF 2019.304, SCM, Theoretical Chemistry, Vrije Universiteit, Amsterdam, The Netherlands, http://www.scm.com.

(S5) Caldeweyher, E.; Ehlert, S.; Hansen, A.; Neugebauer, H.; Spicher, S.; Bannwarth, C.; Grimme, S. A Generally Applicable Atomic-Charge Dependent London Dispersion Correction. *J. Chem. Phys.* **2019**, *150*, 154122.

(S6) Becke, A. D. Density – Functional Thermochemistry. III. The Role of Exact Exchange. *J. Chem. Phys.* ***1993***, *98*, 5648–5652.

(S7) Lee, C.; Yang, W.; Parr, R. G. Development of the Colle-Salvetti Correlation-Energy Formula into a Functional of the Electron Density. *Phys. Rev. B* ***1988***, *37*, 785–789.

(S8) Stephens, P. J.; Devlin, F. J.; Chabalowski, C. F.; Frisch, M. J. Ab Initio Calculation of Vibrational Absorption and Circular Dichroism Spectra Using Density Functional Force Fields. *J. Phys. Chem.* ***1994***, *98*, 11623–11627.

(S9) Becke, A. D. Density-Functional Exchange-Energy Approximation with Correct Asymptotic Behavior. *Phys. Rev. A* **1988**, *38*, 3098–3100.

(S10) van Lenthe, E.; Baerends, E. J.; Snijders, J. G. Relativistic Regular Two-Component Hamiltonians. *J. Chem. Phys.* **1993**, *99*, 4597–4610.

(S11) van Lenthe, E.; Baerends, E. J.; Snijders, J. G. Relativistic Total Energy Using Regular Approximations. *J. Chem. Phys.* **1994**, *101*, 9783–9792.

(S12) Gaussian 16, Revision C.01, Frisch, M. J.; Trucks, G. W.; Schlegel, H. B.; Scuseria, G. E.; Robb, M. A.; Cheeseman, J. R.; Scalmani, G.; Barone, V.; Petersson, G. A.; Nakatsuji, H.; Li, X.; Caricato, M.; Marenich, A. V.; Bloino, J.; Janesko, B. G.; Gomperts, R.; Mennucci, B.; Hratchian, H. P.; Ortiz, J. V.; Izmaylov, A. F.; Sonnenberg, J. L.; Williams-Young, D.; Ding, F.; Lipparini, F.; Egidi, F.; Goings, J.; Peng, B.; Petrone, A.; Henderson, T.; Ranasinghe, D.; Zakrzewski, V. G.; Gao, J.; Rega, N.; Zheng, G.; Liang, W.; Hada, M.; Ehara, M.; Toyota, K.; Fukuda, R.; Hasegawa, J.; Ishida, M.; Nakajima, T.; Honda, Y.; Kitao, O.; Nakai, H.; Vreven, T.; Throssell, K.; Montgomery, J. A., Jr.; Peralta, J. E.; Ogliaro, F.; Bearpark, M. J.; Heyd, J. J.; Brothers, E. N.; Kudin, K. N.; Staroverov, V. N.; Keith, T. A.; Kobayashi, R.; Normand, J.; Raghavachari, K.; Rendell, A. P.; Burant, J. C.; Iyengar, S. S.; Tomasi, J.; Cossi, M.; Millam, J. M.; Klene, M.; Adamo, C.; Cammi, R.; Ochterski, J. W.; Martin, R. L.; Morokuma, K.; Farkas, O.; Foresman, J. B.; Fox, D. J. Gaussian, Inc., Wallingford CT, 2016.

(S13) Grimme, S.; Antony, J.; Ehrlich, S.; Krieg, H. A consistent and accurate ab initio parametrization of density functional dispersion correction (DFT-D) for the 94 elements H-Pu. *J. Chem. Phys*. **2010**, *132*, 154104.

(S14) Grimme, S.; Ehrlich, S.; Goerigk, L. Effect of the Damping Function in Dispersion Corrected Density Functional Theory. *J Comput Chem* **2011**, *32*, 1456–1465.

(S15) Weigend, F.; Ahlrichs, R. Balanced basis sets of split valence, triple zeta valence and quadruple zeta valence quality for H to Rn: Design and assessment of accuracy. *Phys. Chem. Chem. Phys.* **2005**, *7*, 3297–3305.

(S16) Schaefer, A.; Huber, C.; Ahlrichs, R. Fully optimized contracted Gaussian basis sets of triple zeta valence quality for atoms Li to Kr. *J. Chem. Phys.* **1994**, *100*, 5829-5835.

(S17) Mitoraj, M.; Michalak, A. Natural Orbitals for Chemical Valence as Descriptors of Chemical Bonding in Transition Metal Complexes. *J. Mol. Model.* **2007**, *13*, 347–355.

(S18) Mitoraj, M. P.; Michalak, A.; Ziegler, T. A Combined Charge and Energy Decomposition Scheme for Bond Analysis. *J. Chem. Theory Comput.* **2009**, *5*, 962–975.

(S19) Erdogdu, Y; Başköse, Ü. C.; Sağlam, S. Conformational, Structural, Electronic, and Vibrational Investigations on 5-Methyl-4-(2-Thiazolylazo)Resorcinol by FT-IR, FT–Raman, NMR, and DFT. *Chem. Pap.* **2019**, *73*, 1879–1891.

(S20) Drużbicki, K.; Mikuli, E.; Pałka, N.; Zalewski, S.; Ossowska-Chrušciel, M. D. Polymorphism of Resorcinol Explored by Complementary Vibrational Spectroscopy (FT-RS, THz-TDS, INS) and First-Principles Solid-State Computations (Plane-Wave DFT). *J. Phys. Chem. B* **2015**, *119*, 1681–1695.

(S21) Perdew, J. P.; Burke, K.; Ernzerhof, M. Generalized gradient approximation made simple. *Phys. Rev. Lett.* **1996**, *77*, 3865–3868.

(S22) GaussView, Version 6.1.1, Dennington, R.; Keith, T. A.; Millam, J. M. Semichem Inc., Shawnee Mission, KS, **2016**.

(S23) Chemla, D. S.; Zyss, J. Nonlinear optical properties of organic molecules and crystals; Academic Press: London, **1987**.

(S24) Komine, M.; Imoto, K.; Namai, A.; Yoshikiyo, M.; Ohkoshi, S. Photoswitchable Nonlinear-Optical Crystal Based on a Dysprosium–Iron Nitrosyl Metal Assembly. *Inorg. Chem.* **2021**, *60*, 2097–2104.

(S25) Hozumi, T.; Nuida, T.; Hashimoto, K.; Ohkoshi, S. Crystal Structure and Nonlinear Optical Effect of a Pyroelectric Crystal Composed of a Cyano-Bridged Cu−Mo Assembly. *Cryst. Growth Des.* **2006**, *6*, 1736–1737.

(S26) Komine, M.; Chorazy, S.; Imoto, K.; Nakabayashi, K.; Ohkoshi, S. SHG-active Ln^{III}–$[Mo^{I}(CN)_5(NO)]^{3-}$ (Ln = Gd, Eu) magnetic coordination chains: a new route towards non-centrosymmetric molecule-based magnets. *CrystEngComm* **2017**, *19*, 18–22.
(S27) Kawabata, S.; Nakabayashi, K.; Imoto, K.; Klimke, S.; Renz, F.; Ohkoshi, S. Second Harmonic Generation on Chiral Cyanido-Bridged Fe^{II}–Nb^{IV} Spin-Crossover Complexes. *Dalton Trans.* **2021**, *50*, 8524–8532.
(S28) Kumar, K.; Stefanczyk, O.; Chilton, N. F.; Nakabayashi, K.; Imoto, K.; Winpenny, R. E. P.; Ohkoshi, S. Magnetic Properties and Second Harmonic Generation of Noncentrosymmetric Cyanido-Bridged Ln(III)–W(V) Assemblies. *Inorg. Chem.* **2021**, *60*, 12009–12019.
(S29) Ohkoshi, S.; Saito, S.; Matsuda, T.; Nuida, T.; Tokoro, H. Continuous change of second-order nonlinear optical activity in a cyano-bridged coordination polymer. *J. Phys. Chem. C* **2008**, *112*, 13095–13098.
(S30) Kumar, K.; Stefańczyk, O.; Nakabayashi, K.; Imoto, K.; Ohkoshi, S. Studies of Er(III)-W(V) compounds showing nonlinear optical activity and single-molecule magnetic properties. *CrystEngComm* **2019**, *21*, 5882– 5889.
(S31) Kosaka, W.; Nuida, T.; Hashimoto, K.; Ohkoshi, S. Crystal Structure, Magnetic Properties, and Second Harmonic Generation of a Three-Dimensional Pyroelectric Cyano-Bridged Mn–Mo Complex. *Bull. Chem. Soc. Jpn.* **2007**, *80*, 960–962.
(S32) Tsunobuchi, Y.; Kosaka, W.; Nuida, T.; Ohkoshi, S. Magnetization-induced second harmonic generation in a three-dimensional manganese octacyanoniobate-based pyroelectric ferrimagnet. *CrystEngComm* **2009**, *11*, 2051–2053.
(S33) Jankowski, R.; Zakrzewski, J. J.; Zychowicz, M.; Wang, J.; Oki, Y.; Ohkoshi, S.; Chorazy S.; Sieklucka, B. SHG-active NIR-emissive molecular nanomagnets generated in layered neodymium(III)–octacyanidometallate(IV) frameworks. *J. Mater. Chem. C* **2021**, *9*, 10705–10717.
(S34) Chaumont, C.; Mobian, P.; Kyritsakas, N.; Henry, M. Synthesis, Topology and Energy Analysis of Crystalline Resorcinol-Based Oligophenylene Molecules with Various Symmetries. *CrystEngComm* **2013**, *15*, 6854–6862.
(S35) Sheldrick, G. M. A Short History of *SHELX*. *Acta Crystallogr. A* **2008**, *64*, 112–122.
(S36) Sheldrick, G. M. Crystal Structure Refinement with *SHELXL*. *Acta Crystallogr. C Struct. Chem.* **2015**, *71*, 3–8.
(S37) Dolomanov, O. V.; Bourhis, L. J.; Gildea, R. J.; Howard, J. A. K.; Puschmann, H. *OLEX2* : A Complete Structure Solution, Refinement and Analysis Program. *J. Appl. Crystallogr.* **2009**, *42*, 339–341.
(S38) Macrae, C. F.; Sovago, I.; Cottrell, S. J.; Galek, P. T. A.; McCabe, P.; Pidcock, E.; Platings, M.; Shields, G. P.; Stevens, J. S.; Towler, M.; Wood, P. A. *Mercury 4.0* : From Visualization to Analysis, Design and Prediction. *J. Appl. Crystallogr.* **2020**, *53*, 226–235.
(S39) Blatov, V. A.; Shevchenko, A. P.; Proserpio, D. M. Applied Topological Analysis of Crystal Structures with the Program Package ToposPro. *Cryst. Growth Des.* **2014**, *14*, 3576–3586.
